# Safety and immunogenicity of a mosaic vaccine booster against Omicron and other SARS-CoV-2 variants: a randomized phase 2 trial

**DOI:** 10.1038/s41392-022-01295-2

**Published:** 2023-01-03

**Authors:** Nawal Al Kaabi, Yun Kai Yang, Yu Liang, Ke Xu, Xue Feng Zhang, Yun Kang, Yu Qin Jin, Jun Wei Hou, Jing Zhang, Tian Yang, Salah Hussein, Mohamed Saif ElDein, Ze Hua Lei, Hao Zhang, Shuai Shao, Zhao Ming Liu, Ning Liu, Xiang Zheng, Ji Guo Su, Sen Sen Yang, Xiangfeng Cong, Yao Tan, Wenwen Lei, Xue Jun Gao, Zhiwei Jiang, Hui Wang, Meng Li, Hanadi Mekki Mekki, Walid Zaher, Sally Mahmoud, Xue Zhang, Chang Qu, Dan Ying Liu, Jing Zhang, Mengjie Yang, Islam Eltantawy, Peng Xiao, Fu Jie Shen, Jin Juan Wu, Zi Bo Han, Li Fang Du, Fang Tang, Shi Chen, Zhi Jing Ma, Fan Zheng, Ya Nan Hou, Xin Yu Li, Xin Li, Zhao Nian Wang, Jin Liang Yin, Xiao Yan Mao, Jin Zhang, Liang Qu, Yun Tao Zhang, Xiao Ming Yang, Guizhen Wu, Qi Ming Li

**Affiliations:** 1 Sheikh Khalifa Medical City, SEHA, Abu Dhabi, UAE; 2 College of Medicine and Health Sciences, Khalifa University, Abu Dhabi, UAE; 3 China National Biotec Group Company Limited, Beijing, China; 4 The Sixth Laboratory, National Vaccine and Serum Institute (NVSI), Beijing, China; 5 National Engineering Center for New Vaccine Research, Beijing, China; 6 National Institute for Viral Disease Control and Prevention, Chinese Center for Disease Control and Prevention (China CDC), Beijing, China; 7 Clinical Medicine Office, National Vaccine and Serum Institute (NVSI), Beijing, China; 8 Lanzhou Institute of Biological Products Company Limited, Lanzhou, China; 9 Beijing Key Tech Statistical Consulting Co., Ltd, Beijing, China; 10 Beijing Institute of Biological Products Company Limited, Beijing, China; 11 Union 71, Abu Dhabi, UAE; 12 G42 Healthcare, Abu Dhabi, UAE

**Keywords:** Infectious diseases, Vaccines

## Abstract

An ongoing randomized, double-blind, controlled phase 2 trial was conducted to evaluate the safety and immunogenicity of a mosaic-type recombinant vaccine candidate, named NVSI-06-09, as a booster dose in subjects aged 18 years and older from the United Arab Emirates (UAE), who had administered two or three doses of inactivated vaccine BBIBP-CorV at least 6 months prior to enrollment. The participants were randomly assigned with 1:1 to receive a booster dose of NVSI-06-09 or BBIBP-CorV. The primary outcomes were immunogenicity and safety against severe acute respiratory syndrome coronavirus 2 (SARS-CoV-2) Omicron variant, and the exploratory outcome was cross-immunogenicity against other circulating strains. Between May 25 and 30, 2022, 516 adults received booster vaccination with 260 in NVSI-06-09 group and 256 in BBIBP-CorV group. Interim results showed a similar safety profile between two booster groups, with low incidence of adverse reactions of grade 1 or 2. For immunogenicity, by day 14 post-booster, the fold rises in neutralizing antibody geometric mean titers (GMTs) from baseline elicited by NVSI-06-09 were remarkably higher than those by BBIBP-CorV against the prototype strain (19.67 vs 4.47-fold), Omicron BA.1.1 (42.35 vs 3.78-fold), BA.2 (25.09 vs 2.91-fold), BA.4 (22.42 vs 2.69-fold), and BA.5 variants (27.06 vs 4.73-fold). Similarly, the neutralizing GMTs boosted by NVSI-06-09 against Beta and Delta variants were also 6.60-fold and 7.17-fold higher than those by BBIBP-CorV. Our findings indicated that a booster dose of NVSI-06-09 was well-tolerated and elicited broad-spectrum neutralizing responses against divergent SARS-CoV-2 variants, including Omicron and its sub-lineages.

## Introduction

Severe acute respiratory syndrome coronavirus 2 (SARS-CoV-2) continuously evolves to acquire mutations that may change its infectivity and antigenicity. Since the beginning of SARS-CoV-2 pandemic, several variants with increased transmissibility or immune escape capability have emerged, such as Beta, Gamma, Delta, and Omicron.^1–3^ These variants have caused successive waves of SARS-CoV-2 infections and increasing numbers of breakthrough cases.^4,5^ Especially, the recently emerged Omicron is the most antigenically divergent variant, known to date, from the ancestral strain, which consists of several distinct sub-lineages including BA.1, BA.2, BA.3, BA.4, and BA.5.^6–8^ Owing to their much higher transmissibility and greater ability to evade prior immunity, Omicron and its sub-lineages spread rapidly around the world even in the background of high vaccination and previous infection rates in populations.^6–9^ The rapid emergence of new immune-evasive SARS-CoV-2 variants has seriously threatened the efficacy of vaccines currently used, and highlights the urgent need of next-generation vaccines with broad-spectrum protection against divergent SARS-CoV-2 variants.

The receptor-binding domain (RBD) of spike (S) protein directly interacts with the receptor of the host cells, and several studies have indicated that more than 90% of the neutralization in the serum of both convalescent and vaccinated individuals targets the RBD.^10–12^ Given these properties, the RBD is regarded as a major candidate antigen for the development of COVID-19 vaccines. Pre-clinical and clinical studies have demonstrated the non-inferiority of the RBD as an immunogen in comparison to the full-length S protein.^10^ In addition, many isolated broadly neutralizing antibodies to SARS-CoV-2 variants of concern (VOCs) as well as other sarbecoviruses were found to bind to the epitopes on the relatively conserved region of the RBD, which supports the selection of RBD as a potential target for broad-spectrum vaccine design.^10,13–15^

Guided by structural and computational analyses, we have designed a trimeric RBD vaccine, named NVSI-06-07, in which three homologous RBDs derived from the prototype SARS-CoV-2 strain were connected tandemly into a trimeric structure (Supplementary Fig. 1a).^16^ Clinical studies demonstrated that a booster dose of NVSI-06-07 following primary vaccinations of the inactivated vaccine significantly improves the neutralizing antibody (nAb) response against SARS-CoV-2.^17^ NVSI-06-07 has been approved by the United Arab Emirates (UAE) for emergency use. The trimeric RBD that accommodates three RBDs in a single immunogen facilitates us to design a mosaic-type vaccine (NVSI-06-09), which integrates the key mutations from Omicron and other circulating variants into one molecule (Supplementary Fig. 1b). Preclinical studies in animals showed that NVSI-06-09, either used alone or as a booster dose, induced potent and broad immune responses against divergent SARS-CoV-2 variants including Omicron, which may potentially serve as a broad-spectrum vaccine candidate.^18^

Here we reported the interim analysis results of a randomized, double-blind, controlled phase 2 trial conducted in the UAE to evaluate the safety and immunogenicity of NVSI-06-09 as a booster dose in the inactivated vaccine BBIBP-CorV recipients, using the homologous boost with one additional dose of BBIBP-CorV as the control arm. Considering the dominant prevalence of Omicron worldwide currently, immune responses against multiple Omicron sub-lineages were evaluated. Furthermore, as an exploratory study, the cross-neutralizing antibody responses against other VOCs were also detected to assess the broad-spectrum immune responses elicited by NVSI-06-09.

## Results

### Participants and baseline demographic characteristics

**Fig. 1:**
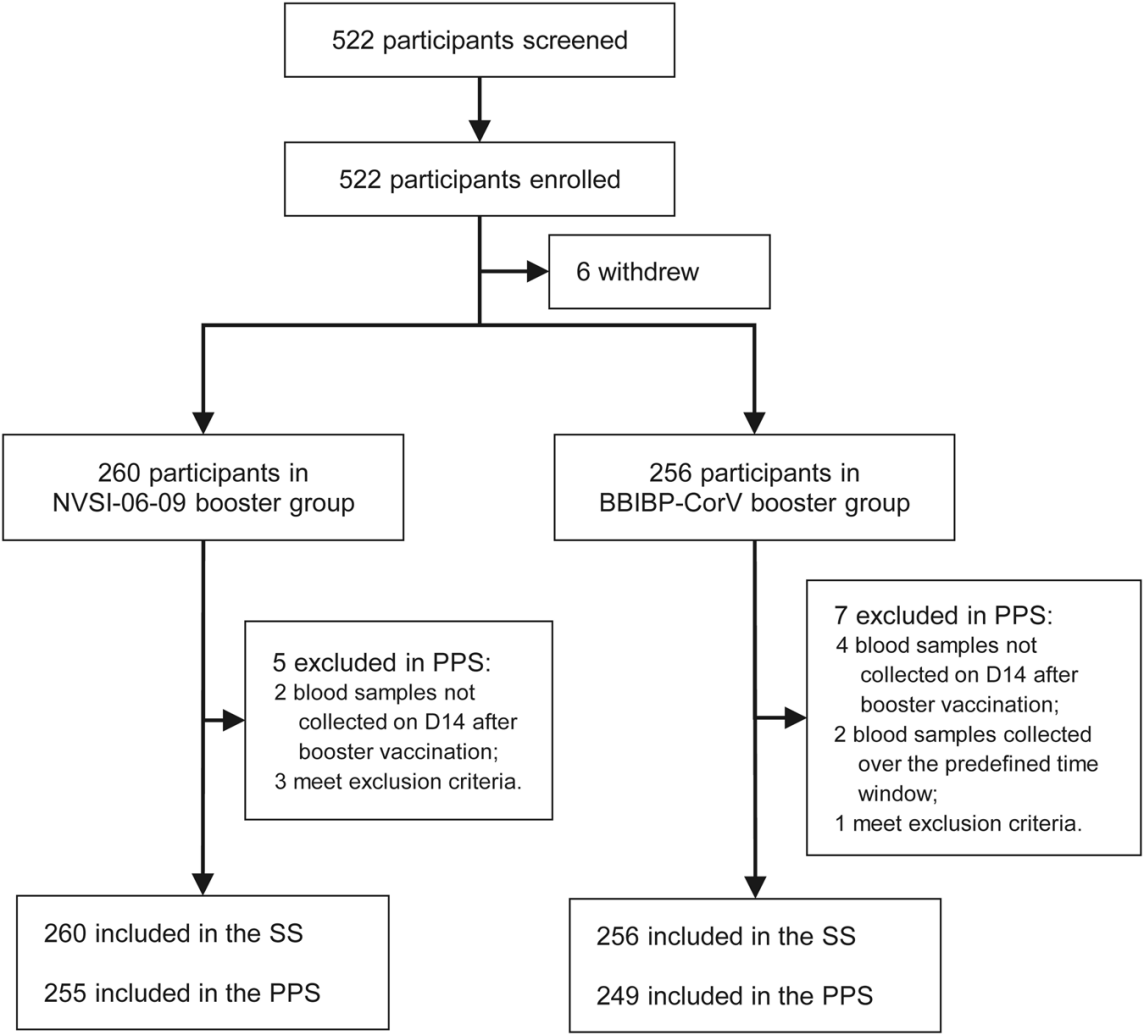
Screening, randomization, and analysis populations. 522 healthy adults were screened and enrolled, of which 6 persons withdrew before vaccination and 516 participated in the study. The participants were randomly assigned to receive a booster injection of either NVSI-06-09 (260 participants) or BBIBP-CorV (256 participants). All these participants that received the booster vaccination were contained in the safety set (SS) for the analysis of vaccine safety. 255 participants in NVSI-06-09 booster group and 249 in BBIBP-CorV booster group who had valid immunogenicity data were included in the per-protocol set (PPS) for the analysis of vaccine immunogenicity

Between May 25 and 30, 2022, a total of 522 healthy adults aged 18 years and older, who had received primary vaccinations of the inactivated vaccine BBIBP-CorV at least 6 months prior, were screened and enrolled. After enrollment, six individuals withdrew from the study and 516 participants received the booster dose of vaccination (Fig. 1). Among these 516 participants, 21 were administered with two doses of BBIBP-CorV and 495 with three doses prior to the study. The participants were randomly assigned to receive a booster dose of NVSI-06-09 (*N* = 260) or a booster vaccination of BBIBP-CorV (*N* = 256). All the 516 participants were contained in the full analysis set (FAS) for baseline characterization as well as in the safety set (SS) for the assessment of vaccine safety. A total of 504 participants (*N* = 255 with NVSI-06-09 and *N* = 249 with BBIBP-CorV) who had valid immunogenicity results without protocol deviation were included in the per-protocol set (PPS) for the assessment of vaccine immunogenicity (Fig. 1).

Male participants accounted for 94.57% of the population, and Asian was the majority (93.99%). Baseline demographic characteristics were well-balanced between the NVSI-06-09 and BBIBP-CorV booster groups. The age composition, male-to-female ratio, race distribution and physiological status were quite similar across groups (all *p* > 0.05) (Table 1). Before booster vaccination, 24 participants from NVSI-06-09 group and 23 from BBIBP-CorV group were tested positive or weak positive for COVID-19 infection by the swab PCR assay (Table 1).

**Table 1:** Demographic characteristics of the participants (FAS)

	NVSI-06-09 (*N* = 260)	BBIBP-CorV (*N* = 256)	*p* value
Age (years)			
Mean (SD)	37.2 (9.1)	37.3 (8.7)	0.8740
Median	36.0	36.5	
Min, Max	19, 62	19, 63	
Sex, *n* (%)			0.4622
Male	244 (93.85)	244 (95.31)	
Female	16 (6.15)	12 (4.69)	
Race, *n* (%)			0.4216
Chinese	0 (0.00)	0 (0.00)	
Asian (except Chinese)	243 (93.46)	242 (94.53)	
Black/African/Caribbean	6 (2.31)	8 (3.13)	
White	11 (4.23)	6 (2.34)	
Multiracial	0 (0.00)	0 (0.00)	
Other	0 (0.00)	0 (0.00)	
Body temperature before immunization (°C)			
Mean (SD)	36.41 (0.19)	36.42 (0.20)	0.5994
Median	36.40	36.40	
Min, Max	36.0, 37.0	36.1, 37.0	
Height (cm)			
Mean (SD)	171.2 (7.2)	170.9 (7.3)	0.5493
Median	171.0	171.0	
Min, Max	151, 195	146, 192	
Weight (kg)			
Mean (SD)	78.69 (14.52)	80.96 (14.34)	0.0745
Median	79.00	79.00	
Min, Max	49.0, 131.0	49.0, 136.0	
Cardiopulmonary auscultation, *n* (%)			1.0000
Normal	260 (100.00)	256 (100.00)	
Abnormal	0 (0.00)	0 (0.00)	
PCR test before immunization, *n* (%)			0.8935
Negative	236 (90.77)	233 (91.02)	
Positive	11 (4.23)	9 (3.52)	
Weak positive	13 (5.00)	14 (5.47)	
Time between last primary vaccination and baseline sample (day)			
Median (IQR)	259 (236–279)	264 (237–284)	0.0926

The *p* value for comparing the NVSI-06-09 and BBIBP-CorV groups was calculated by using two-sided student’s t-test (or rank sum test) for continuous data (after log-transformation of antibodies) and two-sided Chi-square test for non-ordered categorical data

*n* = participant number

*SD* standard deviation, *PCR* polymerase chain reaction, *IQR* interquartile range

### Safety

**Fig. 2:**
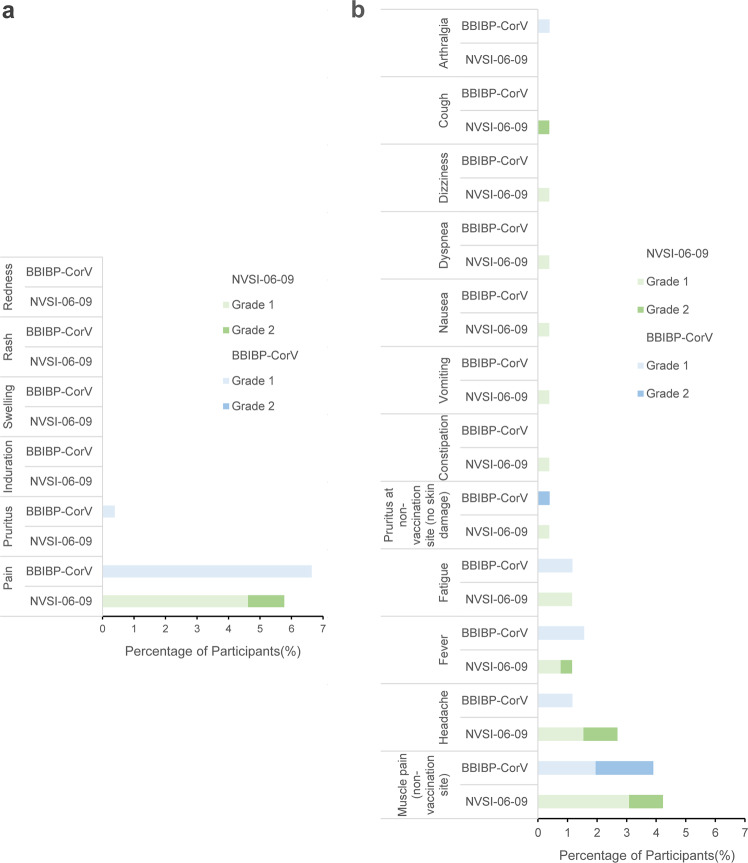
The incidence and severity of adverse reactions in NVSI-06-09 booster group compared with those in BBIBP-CorV booster group. **a** Local adverse reactions reported within 7 days after the administration of booster vaccination. **b** Systemic reactions recorded within 7 days after booster vaccination. The grade of adverse reactions was determined under the guidance of China National Medical Products Administration (NMPA)

At the time of writing this report, no serious adverse event or adverse event of special interest was observed. Within 7 days after booster vaccination, 31 participants (11.92%) in NVSI-06-09 group and 34 (13.28%) in BBIBP-CorV group reported at least one solicited adverse reaction, all of which were ranked as grade 1 or 2. The occurrence of solicited adverse reactions was low and similar between two groups (*p* = 0.6915) (Fig. 2 and Supplementary Table 1). The local adverse reactions reported were injection site pain of grade 1 or 2 by NVSI-06-09 group, and injection site pain (grade 1) and pruritus (grade 1) by BBIBP-CorV group. The systemic adverse reactions reported by both groups were mainly muscle pain (non-vaccination site), headache, fever, and fatigue (Fig. 2 and Supplementary Table 1). Within 30 days post-booster, 8 (3.08%) participants in NVSI-06-09 group and 7 (2.73%) in BBIBP-CorV group reported unsolicited adverse reactions, all of which were grade 1 or 2. The incidence and severity of unsolicited adverse reactions were low and similar between two booster groups (*p* = 1.0000) (Supplementary Table 1). Overall, both the NVSI-06-09 and BBIBP-CorV booster vaccinations showed good tolerance and safety with limited adverse reactions observed, and the safety profile was comparable between two groups.

### Immunogenicity against prototype SARS-CoV-2 virus and omicron BA.1.1 variant

**Fig. 3:**
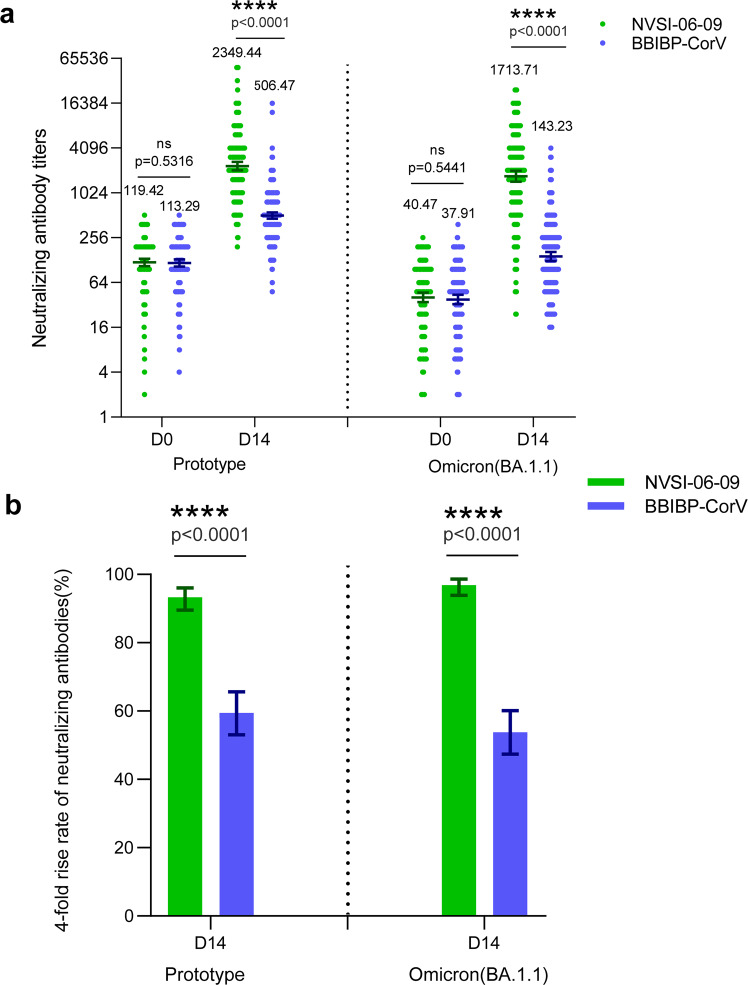
The live-virus neutralizing antibody titers, along with the corresponding fourfold rise rate, against prototype SARS-CoV-2 strain and Omicron BA.1.1 variant induced by the booster vaccination of NVSI-06-09, compared with those boosted with BBIBP-CorV. **a** The anti-prototype and anti-Omicron BA.1.1 neutralizing antibody titers at baseline and on day 14 after booster vaccination. Data are presented as GMTs and 95% CIs. **b** The corresponding fourfold rise rate of neutralizing antibodies from baseline and 95% CIs on day 14 after booster. ****: *p* < 0.0001, ns not significant

The immunogenicity of NVSI-06-09 as a booster dose was evaluated and compared with BBIBP-CorV by using a live-virus microneutralization assay. Before booster vaccination, neutralizing antibodies (nAbs) against the prototype SARS-CoV-2 virus were detectable (nAb titer ≥4) in most participants in NVSI-06-09 (99.61%) and BBIBP-CorV (99.20%) groups, with nAb geometric mean titers (GMTs) of 119.42 (95%CI, 106.47–133.95) and 113.29 (100.50–127.71), respectively (Fig. 3a and Supplementary Table 2). After booster vaccination, nAb response against the prototype virus was significantly improved in both groups, but the post-booster antibody titers induced by NVSI-06-09 booster were distinctly higher than those boosted by BBIBP-CorV. On day 14 post-booster, nAb GMT against the prototype strain in the homologous BBIBP-CorV booster group increased to 506.47 (95% CI, 458.25–559.77), with a 4.47-fold (4.01–4.99) rise compared to the pre-booster baseline. Whereas, neutralizing GMT in the NVSI-06-09 booster group reached 2349.44 (95% CI, 2061.42–2677.70), with a remarkably 19.67-fold (16.79–23.06) increase from baseline. Correspondingly, the fourfold rise rate of nAb titers against the prototype virus induced by NVSI-06-09 was 93.33% (95% CI, 89.54–96.07%), which was much greater than 59.44% (53.06–65.59%) by BBIBP-CorV (*p* < 0.0001) (Fig. 3b and Supplementary Table 2). Some participants exhibited positive (or weak positive) COVID-19 PCR tests before booster vaccination. After excluding these positive participants, similar results were also obtained (Supplementary Table 2). Then, we compared the neutralizing responses between two booster groups stratified by different doses (i.e., two or three doses) of prior primary BBIBP-CorV vaccinations. For the participants receiving two-dose primary vaccinations, the fold rise of nAb GMT induced by the booster of NVSI-06-09 was significantly greater than that of the BBIBP-CorV booster (15.26-fold vs 2.77-fold) (*p* = 0.0042) (Supplementary Table 2). A similar trend was also observed in the participants with three-dose primary vaccinations, where the GMT fold rise was distinctly higher in NVSI-06-09 group than BBIBP-CorV group (19.90-fold vs 4.55-fold) (*p* < 0.0001) (Supplementary Table 2). But within NVSI-06-09 or BBIBP-CorV booster group, no significant difference was found in the fold rise of nAb GMT between the participants who previously received two and three doses of primary vaccinations of BBIBP-CorV (*p* > 0.05) (Supplementary Tables 3 and 4).

At pre-booster baseline, 96.86% participants in NVSI-06-09 group and 97.19% in BBIBP-CorV group exhibited detectable nAbs against the Omicron BA.1.1 variant, with the GMT values of 40.47 (95% CI, 34.86–46.97) and 37.91 (32.64–44.04), respectively (Fig. 3a and Supplementary Table 5). The baseline level of nAb GMT against Omicron BA.1.1 variant was obviously lower than that against the prototype strain, indicating that the Omicron BA.1.1 variant was partially resistant to two- or three-dose BBIBP-CorV vaccinations. Both NVSI-06-09 and BBIBP-CorV booster vaccinations markedly elevated Omicron BA.1.1-specific nAb response, and similar to that of prototype virus, the nAb level against Omicron BA.1.1 variant elicited by NVSI-06-09 was dramatically higher than that induced by BBIBP-CorV. On day 14 post-booster, the increase of Omicron BA.1.1-specific nAbs from pre-booster baseline by NVSI-06-09 arrived at 42.35-fold (95% CI, 35.15–51.03) with GMT of 1713.71 (95% CI, 1447.12–2029.41), and 3.78-fold (3.40–4.20) by BBIBP-CorV with GMT of 143.23 (124.48–164.80). Correspondingly, the fourfold rise rate of nAb titers against the Omicron BA.1.1 induced by NVSI-06-09 booster was also significantly greater than that by BBIBP-CorV booster (96.86% vs 53.82%) (*p* < 0.0001) (Fig. 3b and Supplementary Table 5). After excluding the participants with pre-booster positive COVID-19 infections, similar results were also obtained (Supplementary Table 5). When investigating the participants separately with two or three primary doses, similar superiority was observed in NVSI-06-09 group. The NVSI-06-09 booster elicited 27.48-fold (95% CI, 7.32–103.15) and 43.18-fold (35.78–52.11) rises of Omicron BA.1.1-specific nAb GMT in participants receiving two and three primary doses, respectively. In contrast, the BBIBP-CorV booster only induced a 1.49-fold (95% CI, 0.94–2.37) increase in two-dose regimen and a 3.91-fold (3.51–4.35) in three-dose regimen (Supplementary Table 5). The immune response boosted by NVSI-06-09 was significantly higher than that by BBIBP-CorV in both regimens. Within BBIBP-CorV booster group, the fold rise of nAb GMT in subjects who received three doses of primary vaccination was significantly greater than those who received two doses (*p* = 0.0008). But within NVSI-06-09 booster group, no significant difference in the immune response was observed between participants receiving two and three doses of primary vaccinations (*p* > 0.05) (Supplementary Tables 6 and 7).

As demonstrated by the results presented above, the BBIBP-CorV booster elicited similar or lower nAb GMT fold rise against Omicron BA.1.1 variant than that against prototype strain (3.78-fold vs 4.47-fold). By contrary, the NVSI-06-09 booster induced a much greater GMT fold rise against Omicron BA.1.1 strain than that against prototype virus (42.35-fold vs 19.67-fold). These results indicated that the NVSI-06-09 booster exhibited remarkably better immunogenicity against SARS-CoV-2 virus, especially Omicron BA.1.1 variant.

Then, we further investigated whether the gender and age of participants have influences on the immune response of booster vaccinations. Statistical analyses were performed after stratifying participants into male and female subgroups, as well as into different age subgroups. The analysis results showed that the nAb responses against prototype virus and Omicron BA.1.1 variant boosted by either NVSI-06-09 or BBIBP-CorV were gender-independent and also age-independent. The post-booster nAb GMTs were comparable between male and female participants (all *p* > 0.05) (Supplementary Tables 8–11), and also similar between different age subgroups (all *p* > 0.05) (Supplementary Tables 12–19), except that the adjusted nAb GMT boosted by BBIBP-CorV in ≥60 years subgroup was significantly higher than that of 45–59 years subgroup (*p* = 0.0005). Nevertheless, it should be pointed out that the number of female subjects in the trial was far less than male, and elder subjects far less than younger. The immunogenicity of the NVSI-06-09 booster in female population as well as in elder population should be investigated further in the future study.

### Cross-reactive immunogenicity against other Omicron sub-variants

SARS-CoV-2 Omicron variant evolves rapidly, and many sub-variants have emerged. Owing to its higher transmissibility and immune-evasive capability, Omicron BA.2 replaced BA.1 quickly, and was soon replaced by BA.4 and BA.5 afterwards. Currently, BA.4 and BA.5 have become the dominantly circulating variants worldwide. Besides Omicron BA.1.1, the cross-reactive immunogenicity of NVSI-06-09 against BA.2, BA.4, and BA.5 was also evaluated by using the live-virus microneutralization assay. The nAb responses against these three Omicron sub-variants were tested in all participants in PPS, and compared between the NVSI-06-09 and BBIBP-CorV booster groups.

**Fig. 4:**
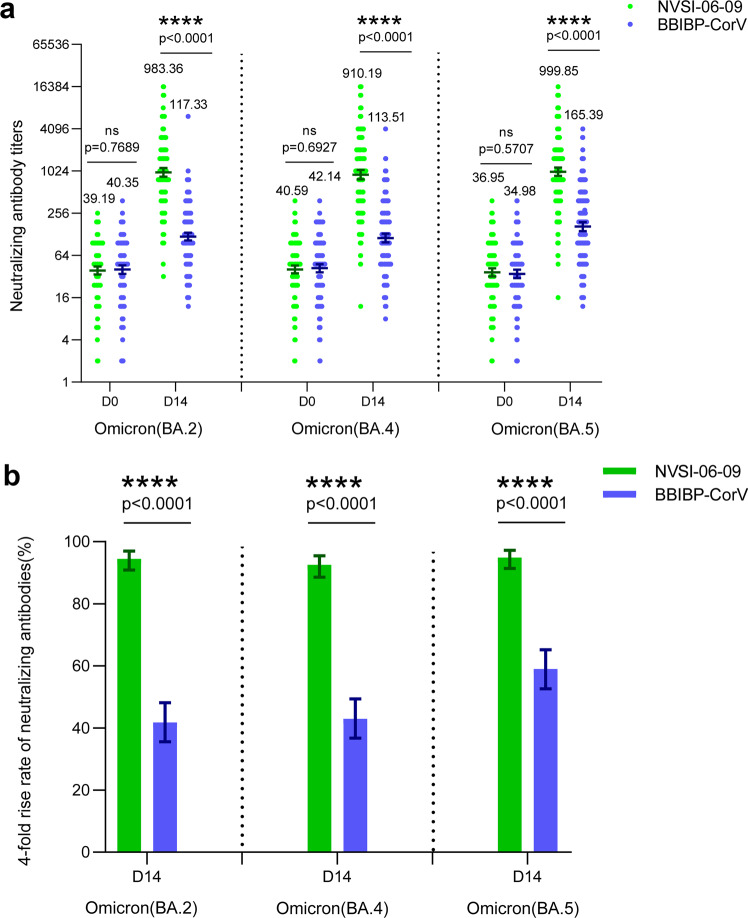
The cross-neutralizing antibody titers, along with the corresponding four-fold rise rate, against Omicron BA.2, BA.4, and BA.5 induced by the booster vaccination of NVSI-06-09, compared with those boosted with BBIBP-CorV. **a** The anti-BA.2, anti-BA.4, and anti-BA.5 neutralizing antibody titers at baseline and on day 14 after booster vaccination. Data are presented as GMTs and 95% CIs. **b** The corresponding four-fold rise rate of neutralizing antibodies from baseline and 95%CIs on day 14 after the booster. ****: *p* < 0.0001, ns not significant

Before booster vaccination, 96.85%-97.64% participants in NVSI-06-09 group, and 95.58–97.99% in BBIBP-CorV group had detectable nAbs against the Omicron BA.2, BA.4, and BA.5 sub-variants. The anti-BA.2, anti-BA.4, and anti-BA.5 neutralizing baseline GMTs were similar between two groups, i.e., 39.19 (95% CI, 34.27–44.82), 40.59 (35.75–46.10) and 36.95 (32.38–42.16) in the NVSI-06-09 booster group, and 40.35 (35.05–46.45), 42.14 (36.80–48.25), and 34.98 (30.53–40.09) in the BBIBP-CorV booster group (Fig. 4a and Supplementary Tables 20–22). The pre-booster GMTs against these three Omicron sub-variants were noticeably less than that against prototype virus, implying less sensitive of these Omicron sub-variants to primary BBIBP-CorV vaccinations. On day 14 after booster vaccination, the BA.2-, BA.4- and BA.5-specific neutralizing GMTs in BBIBP-CorV booster group increased to 117.33 (95% CI, 103.07–133.55), 113.51 (98.50–130.81) and 165.39 (142.99–191.30), with 2.91-fold (95% CI, 2.60–3.25), 2.69-fold (2.38–3.05) and 4.73-fold (4.10–5.45) increase from baselines, respectively. By contrast, a dramatically higher neutralizing response was induced by NVSI-06-09 booster, in which the BA.2-, BA.4-, and BA.5-specific neutralizing GMTs reached 983.36 (95% CI, 852.25–1134.64), 910.19 (779.53–1062.75) and 999.85 (872.41–1145.90) with the GMT fold rises of 25.09-fold (95% CI, 21.17–29.73), 22.42-fold (18.78–26.77) and 27.06-fold (23.04–31.78), respectively (Fig. 4a and Supplementary Tables 20-22). The remarkably better immunogenicity of NVSI-06-09 can also be observed from the four-fold rise rates in nAb titers. In participants from NVSI-06-09 booster group, the fourfold rise rates of anti-BA.2, anti-BA.4 and anti-BA.5 neutralizing titers were 94.49% (95% CI, 90.92–96.95%), 92.52 % (95% CI, 88.56–95.44%) and 94.88% (95% CI, 91.41–97.25%), respectively, which were significantly higher than 41.77% (95% CI, 35.57–48.16%), 42.97% (95% CI, 36.74–49.37%) and 59.04% (95% CI, 52.65–65.20%) in BBIBP-CorV booster group (all *p* < 0.0001) (Fig. 4b and Supplementary Tables 20–22). After excluding the participants with pre-booster positive COVID-19 infections, similar results were obtained (Supplementary Tables 20–22). Similar results were also obtained when participants were stratified by two-dose or three-dose primary vaccination regimens. Whether two or three doses of BBIBP-CorV were administered previously by the participants, a booster dose of NVSI-06-09 induced much higher nAb responses against Omicron BA.2, BA.4, and BA.5 sub-variants than those boosted by BBIBP-CorV (Supplementary Tables 20–22). But within either NVSI-06-09 or BBIBP-CorV booster group, the post-booster nAb GMTs in subjects primed with two doses of BBIBP-CorV were comparable to those primed with three doses (all *p* > 0.05) (Supplementary Tables 23–28). In addition, statistical analyses of the participants with different sexes and ages demonstrated that the post-booster nAb GMTs against Omicron BA.2, BA.4, and BA.5 sub-variants boosted by NVSI-06-09 and BBIBP-CorV were gender-independent (all *p* > 0.05) (Supplementary Tables 29–34) and also age-independent (all *p* > 0.05) (Supplementary Tables 35–46).

### Cross-reactive immunogenicity against Beta and Delta variants

NVSI-06-09 is designed as a potential broad-spectrum COVID-19 vaccine. Besides the Omicron variants, the cross-reactive immunogenicity of NVSI-06-09 booster vaccination was also tested against other immune-evasive variants including Beta and Delta, using BBIBP-CorV as a comparison. A subset of serum samples with sequential numbers collected from 99 participants in NVSI-06-09 group and 100 participants in BBIBP-CorV group were used to evaluate the cross-neutralization against Beta and Delta variants.

**Fig. 5:**
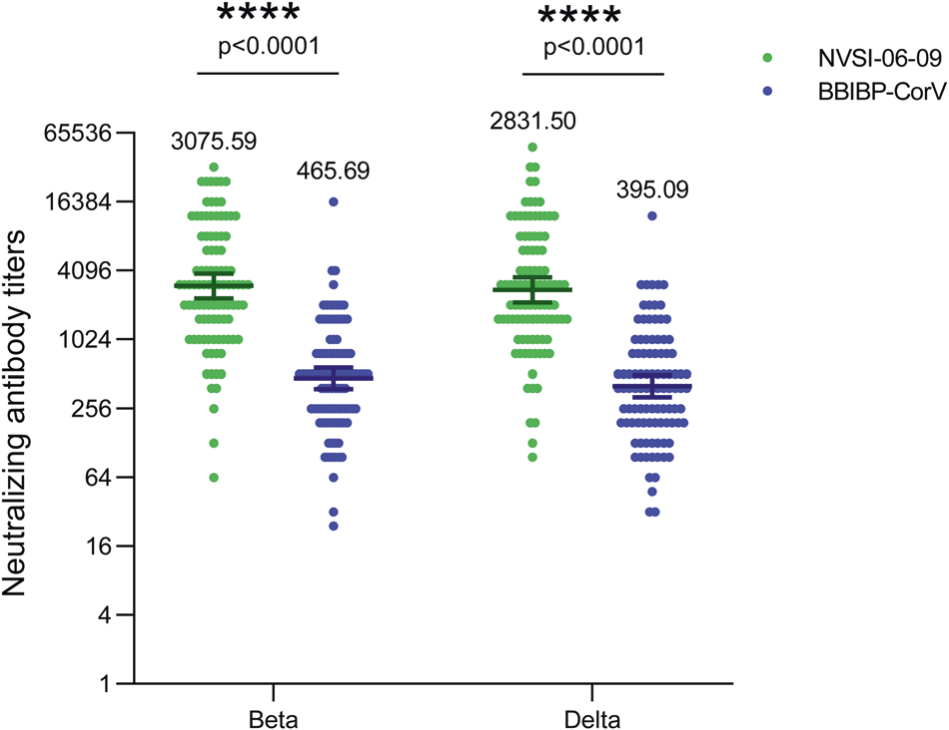
The cross-neutralizing antibody titers against Beta and Delta variants induced by the booster vaccination of NVSI-06-09, compared with those boosted with BBIBP-CorV. On 14 days after booster vaccination, a subset of 99 serum samples in NVSI-06-09 booster group and 100 samples in BBIBP-CorV booter group with sequential numbers were tested by using live-virus microneutralization assay. Data are presented as GMTs and 95% CIs. ****: *p* < 0.0001

The results showed that nAb GMTs against Beta and Delta variants induced by NVSI-06-09 booster were 3075.59 (95% CI, 2393.00–3952.89) and 2831.50 (2200.42–3643.58), respectively, which were 6.60-fold and 7.17-fold higher than those boosted by BBIBP-CorV with the GMT values of 465.69 (95% CI, 376.75–575.63) and 395.05 (316.82–492.58) (Fig. 5 and Supplementary Table 47). In particular, statistical analyses showed that the nAb GMT against Delta variant induced by the booster vaccination of NVSI-06-09 was comparable to that against the prototype virus (*p* = 0.1536), and the nAb GMT against Beta variant was significantly higher than that against the prototype strain (*p* = 0.0408) (Supplementary Table 47). The reason for this phenomenon is that several mutations carried by NVSI-06-09 were taken from the Beta and Delta variants. Therefore, the immunogen of NVSI-06-09 is more like the RBDs of Beta and Delta variants, instead of that of the prototype strain.

## Discussion

NVSI-06-09 is a potentially broad-spectrum vaccine using a mosaic-type trimeric RBD protein as the antigen, which combined key mutations from Omicron and other circulating variants into a single molecule. The interim results of the current study showed that a booster injection of NVSI-06-09 in participants who had completed two- or three-dose vaccinations of BBIBP-CorV was safe and well-tolerant. The safety profile of NVSI-06-09 booster was very similar to that of BBIBP-CorV booster, which was also comparable to the safety of two-dose primary vaccination with BBIBP-CorV as reported by Xia et al.^19^ Although most of participants in this trial had received three doses of BBIBP-CorV prior to enrollment, an additional booster dose of NVSI-06-09 or BBIBP-CorV did not bring additional safety risk of concern. For immunogenicity, NVSI-06-09 booster induced not only much higher but also much broader neutralizing responses than BBIBP-CorV booster against divergent SARS-CoV-2 strains. After booster vaccination, the nAb GMT against prototype virus in NVSI-06-09 group was 4.57-fold (after adjustment) higher than that in BBIBP-CorV group. Whereas, the ratios of neutralizing GMT after adjustment between two groups against Omicron BA.1.1, BA.2, BA.4, and BA.5 were improved to 11.61-fold, 8.50-fold, 8.15-fold, and 5.92-fold, and those against Beta and Delta variants were improved to 6.60-fold and 7.17-fold. It has been widely reported that nAb level is highly correlated with immune protection against SARS-CoV-2.^20–22^ The stronger and broader nAb titers induced by NVSI-06-09 indicate that this vaccine may serve as a promising booster vaccine to better combat divergent SARS-CoV-2 variants, especially the immune-evasive variant of Omicron and its sub-lineages.

WHO has recommended a booster dose or even a second booster dose of COVID-19 vaccine to better combat the pandemic of SARS-CoV-2 (https://www.who.int/publications/). Safety of the vaccine is vital for the large-scale rollout of booster vaccinations. NVSI-06-09 is highly immunogenic and also has a good safety profile, supporting it as a promising booster candidate. Its high safety is attributed to the use of aluminum hydroxide as the adjuvant, whose safety has been extensively validated. Our data indicated that aluminum adjuvant combined with a well-designed antigen is a safe yet effective choice for booster vaccination in large-scale population.

Many previous studies have demonstrated that heterologous booster vaccination may elicit more potent and also broad immune responses than homologous vaccination, especially for inactivated vaccines.^23–27^ Our previous studies also showed that the heterologous booster vaccination of NVSI-06-07, the first-generation trimeric RBD-based vaccine targeting the prototype virus, following two-dose primary vaccinations of the inactivated vaccine BBIBP-CorV induced much higher nAb response than the homologous booster vaccination of BBIBP-CorV.^17^ As an advanced vaccine developed based on NVSI-06-07, NVSI-06-09 exhibited stronger immunogenic activities against the immune-evasive variants, including Omicron. Preclinical animal studies showed that NVSI-06-09 elicited higher cross-neutralization to Omicron and other circulating variants than NVSI-06-07.^18^ Comparing the results of this trial with the data from the trial on NVSI-06-07 reported previously,^17^ it was found that the nAb levels boosted by NVSI-06-09 were further remarkably higher than those by NVSI-06-07 against SARS-CoV-2 variants, especially Omicron. The result indicates that besides the heterologous booster strategy, the design of the mosaic-type immunogen distinctly contributes to the immunogenic superiority of NVSI-06-09 as a booster vaccine against divergent SARS-CoV-2 variants. It should be mentioned that most of the participants in this trial had received three doses of BBIBP-CorV before booster vaccination, and those in the trial for NVSI-06-07 received two doses. A thorough head-to-head comparison between NVSI-06-09 and NVSI-06-07 for the immunogenicity will be conducted in the future.

To date, COVID-19 vaccines authorized by WHO for emergency use are all targeting the prototype SARS-CoV-2 virus. Numerous studies have shown that after the primary-series vaccination of prototype vaccine, the nAb titers against Beta, Delta, and Omicron variants decreased by 1.07–16.32-fold, 1.0–7.2-fold, and 1.15–109.87-fold, respectively, when compared with that against the prototype virus.^28^ Although booster vaccinations could significantly improve the immune responses, the neutralizations against Beta, Delta, and Omicron variants were still 0.86–15.00-fold, 0.76–9.23-fold, and 1.15–26.87-fold, respectively, lower than that against the prototype strain.^28^ Even within the Omicron variant, BA.4/BA.5 sub-variants also largely evaded the neutralization induced by BA.1 infection or vaccination, with a 2.6–8.0-fold drop in the nAb titers.^29–31^ Therefore, there is an urgent need to develop broad-spectrum vaccines to fight against immune-evasive variants. The findings from our study demonstrated that a booster shot of NVSI-06-09 not only significantly improved but also broadened the immune response to SARS-CoV-2 variants, which may serve as a candidate vaccine with potentially broad-spectrum immune activity.

Aiming at the defense against the emergence of immune-evasive variants of SARS-CoV-2, several variant-specific vaccines have been developed. Utilizing the inactivated virus-based, mRNA-based, and recombinant protein-based platforms, multiple updated vaccines specific to Beta, Delta, Omicron BA.1 or Omicron BA.2 have been developed.^30,32–38^ Results of animal studies and clinical trials have shown that these variant-targeting vaccines could induce higher nAb responses than the ancestral vaccine against these specific variants. Furthermore, variant-specific vaccines along with prototype vaccines have been used in combination to achieve cross-neutralizing responses against divergent SARS-CoV-2 strains (ClinicalTrials.gov identifier: NCT05365724, NCT05381350, NCT05382871).^30,33,36,38^ We, alternatively, adopt a new strategy on the principle of mosaic-type antigen for vaccine development to enable broad neutralization against SARS-CoV-2 variants. This strategy has been successfully applied in the development of broad-spectrum vaccines against other highly mutated viruses, such as HIV and influenza,^39–43^ and several studies indicated that the mosaic antigen could elicit broader antibody responses than those induced by the mixture of the corresponding monovalent antigens.^41^ Moreover, compared with the conventional production of multivalent vaccine, the design of mosaic vaccine enables the realization of multivalent immunity with a single immunogen, which facilitates the manufacturing process. In addition, the broad-spectrum immune activity of the mosaic vaccine may alleviate the frequent modifications of the vaccine facing the continuously emerging variants, which may improve the administration adherence. Our study results demonstrated that the development of mosaic-type vaccines may provide a promising approach to better control the pandemic of the existing and future SARS-CoV-2 variants.

The uncertainty of SARS-CoV-2 evolution may further lead to increased transmissibility, immune evasion properties, and virulence of the virus in the future. It is necessary to accumulate effective technologies to better cope with new variants. The construction of mosaic vaccine may serve as one of the effective strategies against divergent SARS-CoV-2 variants.

Our study has some limitations. Firstly, only a small percentage of participants who received two doses of BBIBP-CorV were enrolled in the study. The potential influence of primary dosing frequency of BBIBP-CorV on the immune response boosted by NVSI-06-09 may not be fully revealed. Secondly, very few female volunteers and also very small number of elder individuals participated in the trial, and thus the results obtained may not well reflect the potential immune response in females as well as in elders. Thirdly, NVSI-06-09 is a relatively broad-reactive vaccine, however, it also needs to be updated to maintain high nAb titers when new variants with significant antigenic changes emerge. Fourthly, the clinical data about the long-term immunity are not yet available in this ongoing trial. The results will be reported once the data are obtained.

## Materials and methods

### Trial design

We conducted a randomized, double-blind, controlled phase 2 trial to assess the safety and immunogenicity of the mosaic-type trimeric RBD vaccine, named NVSI-06-09, as a heterologous booster shot following primary vaccination of an inactivated vaccine BBIBP-CorV. The homologous boost of another dose of BBIBP-CorV was used as control in the study. The trial was reviewed and approved by the Abu Dhabi Health Research and Technology Ethics Committee of UAE, and conducted under the principles of the International Conference on Harmonization-Good Clinical Practice (ICH-GCP) and the Declaration of Helsinki (with amendments). This study also met the local legal and regulatory requirements of UAE. The sponsors of the study are China National Biotec Group Co., Ltd (CNBG) of Sinopharm, National Vaccine and Serum Institute (NVSI) of Sinopharm CNBG, Lanzhou Institute of Biological Products Co., Ltd (LIBP) of Sinopharm CNBG, and Beijing Institute of Biological Products Co., Ltd (BIBP) of Sinopharm CNBG. The study protocol is provided in the Supplementary Protocol. The trial is registered with ClinicalTrials.gov (NCT05293548), and the study is still ongoing.

### Participants

Eligible trial subjects were healthy men and nonpregnant women, aged 18 years or above, who had previously vaccinated with two or three doses of BBIBP-CorV at least 6 months prior to enrollment. All participants signed an informed consent form. During screening, healthy status of each volunteer was assessed by inquiry and physical examination. Volunteers who had previously received any other COVID-19 vaccine instead of the BBIBP-CorV were excluded. Individuals whose axillary temperature ≥37.3 °C (or forehead temperature ≥37.8 °C) or allergic to any components of the vaccine were also excluded. Other exclusion criteria included history of thrombocytopenia or other coagulation disorders; immunological impairment or immunocompromise; receipt of any blood products or immunoglobulin therapy within 3 months prior to enrollment; serious chronic disease; receipt of any other inactivated vaccines in the past 14 days, or any live attenuated vaccines post one month; receipt of any investigational drugs within 6 months prior to enrollment; and other vaccination-related contraindications considered by the investigator.

### Studied vaccines

NVSI-06-09 is a mosaic-type trimeric RBD vaccine, recombinantly expressed by Chinese hamster ovary cells, which covalently combined three heterologous RBDs into a single molecule to present potentially broad immunological coverage. Within NVSI-06-09, one RBD is derived from Omicron BA.1 variant, and the other two are artificially designed harboring the key residues from other VOCs and variants of interests (VOIs).^18^ One of the artificially designed RBD carries five mutations of K417N, L452R, T478K, F490S, and N50Y, and the other RBD contains three mutations of K417T, S477N, and E484K (Supplementary Fig. 1b). These mutations have appeared in several VOCs and VOIs, including Alpha, Beta, Gamma, Delta, Omicron, Mu, and Lambda, and have been listed as frequently occurring mutations in divergent SARS-CoV-2 variants.^18^ This vaccine was developed by the NVSI of Sinopharm CNBG, and provided in the liquid form at a single dose of 0.5 ml/vial, containing 20 μg antigen protein, 0.3 mg aluminum hydroxide, 0.39 mg histidine and 4.38 mg sodium chloride for injection. The inactivated vaccine BBIBP-CorV, adopted as a control vaccine in this study, was developed by the BIBP of Sinopharm CNBG using the prototype SARS-CoV-2 virus (19nCoV-CDC-Tan-HB02 strain). BBIBP-CorV is manufactured by culturing virus in Vero cells and inactivated by β-propionolactone,^44^ and provided in a single dose of 0.5 ml per vial containing 6.5 U antigen. BBIBP-CorV is one of the COVID-19 vaccines authorized by the World Health Organization (WHO) and has been widely used in many countries around the world. Both NVSI-06-09 and BBIBP-CorV are transported and stored at 2–8 °C.

### Randomization and blinding

In this study, the participants were randomly assigned in a ratio of 1:1 to receive either a heterologous booster dose of NVSI-06-09 or a homologous booster dose of BBIBP-CorV. For participant allocation, eligible persons were assigned to two booster groups using a stratified blocked randomization method by SAS 9.4 software, in which stratification was based on different doses (two or three doses) of BBIBP-CorV that they received prior to enrollment and the block size was set to 4. The vaccine randomization list was also generated by SAS 9.4 software using the block randomization method with a block size of 4. Both participant and vaccine randomization lists were generated by an unblinded statistician, and then imported into the Interactive Web Response System (IWRS). After enrollment, each participant was assigned a randomization number from IWRS. At the clinical site, a vaccine number was also obtained from IWRS for vaccination accordingly. If the vaccine vial is damaged, a new vaccine number will be assigned to the participant.

Participants and clinical operation team involved in safety data collection and immunogenicity assessments were blind to treatment allocation during the trial. Vaccine preparation was done by independent personnel to ensure identical appearance between the studied and control vaccines. Individuals involved in randomization and blinding did not participate in other trial operations.

### Procedures

At the clinical site, eligible participants were randomly assigned to receive a booster vaccination with NVSI-06-09 or BBIBP-CorV. The vaccine was administered intramuscularly to the participants’ upper arm muscles. After observation for at least 30 min, participants can leave the clinical site. Solicited local adverse events at injection site (including pain, pruritus, induration, swelling, rash, and redness) and solicited systemic adverse events (including non-vaccination site muscle pain, headache, fever, fatigue, pruritus at non-vaccination site, arthralgia, constipation, vomiting, nausea, cough, dyspnea, dizziness, diarrhea, dysphagia, anorexia, abnormal skin mucosa, and acute allergic reaction) were recorded within 7 days post-vaccination. Unsolicited adverse events were collected within 30 days after the receipt of vaccination. Serious adverse events and the adverse events of special interest were monitored for 12 months after vaccination. All the reported adverse events were reviewed and verified by investigators, and the grades of adverse events were determined based on the relevant guidance of China National Medical Products Administration. Blood samples were taken from participants for immunogenicity assessment on day 0, day 14, day 28, and at 3 months, 6 months, 9 months, and 12 months, respectively.

### Study outcomes

The primary outcomes were the immunogenicity and safety of the booster vaccination of NVSI-06-09 against SARS-CoV-2 Omicron variant, in comparison with the boost of BBIBP-CorV, on day 14 after the vaccination. The immunogenicity was evaluated by the geometric mean titers (GMTs) of anti-Omicron neutralizing antibodies (nAbs), and the four-fold rise rate of the neutralizing GMTs from baseline. Anti-Omicron nAb titers were measured by live-virus microneutralization assays. The safety was assessed by occurrence and severity of adverse reactions. NVSI-06-09 was designed as a potential broad-spectrum COVID-19 vaccine. To assess the broad immune responses induced by a booster dose of NVSI-06-09, the cross-neutralizing antibody GMTs against other SARS-CoV-2 strains, including the prototype, Beta and Delta, were also detected as an exploratory study.

### Laboratory tests

NAb titers in the sera of participants were detected by using the live-virus microneutralization assay based on inhibition of cytopathic effects (CPE). A total of six SARS-CoV-2 live viruses, including Omicron BA.1.1 (NPRC 2.192100005), Omicron BA.2 (NPRC 2.192100010), Omicron BA.4 (NPRC 2.192100012), Omicron BA.5 (NPRC 2.192100015), prototype (QD-01), Beta (GD84) and Delta (GD96) strains, were tested. These viruses were provided by the National Institute for Viral Disease Control and Prevention, the Chinese Center for Disease Control and Prevention (China CDC), Beijing, China. The live-virus microneutralization assays were also performed in the Biosafety Level-3 Laboratory (BSL-3) of the National Institute for Viral Disease Control and Prevention, China CDC.

In the CPE-based live-virus microneutralization assay, serum samples were firstly inactivated by heat at 56 °C for 30 min, followed by a series of twofold dilutions starting from 1:4 for pre-booster serum samples and 1:8 for post-booster samples. The diluted serum was mixed with an equal volume of SARS-CoV-2 solution, which contains 100 TCID_50_ of live virus, in the wells on the 96-well plate. Then the plate was incubated at 37 °C with 5% carbon dioxide (CO_2_) for 2 h. Subsequently, Vero cells of (1.0–2.0) × 10^5^ per mL, cultured in the medium 199 containing 5% fetal bovine serum, were added to the wells, and incubated at 37 °C ± 1 °C with 5% CO_2_ for 5–7 days. After incubation, cytopathic changes in Vero cells were examined, and nAb titers were determined as the reciprocal of serum dilution at which 50% of CPE was inhibited. The titer of the serum below the limit of quantification was reported as the half value of the quantification limit. In the assay, both negative and positive reference sera were tested as controls. The reference sera and Vero cells were provided by the National Institute for Food and Drug Control of China. Cell-control was also set and the titer of the virus was also re-titrated in the assay.

### Statistical analysis

The sample size of trial subjects was determined using the Power Analysis and Sample Size (PASS15.0) software. It was assumed that the anti-Omicron nAb GMT in the experimental group was superior to that in the control group on day 14 after vaccination, with a superiority margin after log10 transformation of 0.0212. The other assumption parameters include standard deviation of the antibody GMT after log10 transformation of 0.55, the type I error (one-sided) probability of 0.025, the expected power of 0.9, and a 20% dropout rate. As such, a total of 398 participants were required. It was further assumed that the nAb level in the experimental group was non-inferior to that in the control group on day 14 post-vaccination. When using the non-inferiority threshold after log10 transformation of −0.17609 and the other parameters remained the same as above, a total of 516 participants were required to achieve 0.9 power to detect non-inferiority. Taken together, the sample size was determined to be 516, with 258 in the experimental group and the other 258 in the control group.

Baseline characteristics were assessed based on the FAS, which includes all randomized participants whose baseline data were available and valid. Safety analysis was carried out on the SS that includes all participants who received the booster vaccination. Immunogenicity was analyzed based on the PPS including all participants who received the booster vaccination and had valid pre- and post-vaccination immunogenicity data.

For baseline characterization, continuous and categorical characteristics between groups were compared using two-sided Student’s *t* test and two-sided Chi-square test, respectively. In the safety analysis, both counts and percentage of adverse reactions were presented, and two-sided Fisher’s exact test was used to determine whether there was a significant difference between the safety of two groups. In immunogenicity analysis, the GMT of nAbs, the fold rise of the neutralizing GMT from baseline and the ratio of GMTs between the experimental and control groups, and the associated 95% confidence interval (CI) were calculated. In addition, the four-fold rise rates of nAb titers from baselines and 95% CIs were calculated by the Clopper–Pearson method. The differences in the fourfold rise rates between the experimental and control groups as well as the associated 95% CIs were evaluated by Cochran–Mantel–Haenszel method considering stratification factors. The adjusted nAb GMT and the corresponding *p* value were calculated using covariance analysis with least square method. The nAb GMTs between the two groups were compared using the two-sided grouped t-test after log10 transformation of the titers, and the difference in the neutralizing GMT fold rise between the two groups was also analyzed using the two-sided grouped t-test. All the above analyses were performed using the SAS (version 9.4) software.

## Supplementary information

Supplementary Figure S1 and Tables S1-S47

Supplementary Protocol

## Data Availability

The clinical trial is still ongoing, and the data will be available when the trial is complete upon request to the corresponding author (Q.M.L.). After study proposals are approved, data can be shared through secure online platforms.
