# Supplementary material for: Safety and immunogenicity of a mosaic vaccine booster against Omicron and other SARS-CoV-2 variants: a randomized phase 2 trial
Source: Signal Transduct Target Ther. 2023 Jan 3;8:20. doi: 10.1038/s41392-022-01295-2 (PMC9808735; doi:10.1038/s41392-022-01295-2)
Supplement: Supplementary file 1 — Supplementary Figure S1 and Tables S1-S47 [file 41392_2022_1295_MOESM1_ESM.docx]

Supplementary Materials for

Safety and immunogenicity of a mosaic vaccine booster against Omicron and other SARS-CoV-2 variants: a randomized phase 2 trial

Nawal Al Kaabi, Yun Kai Yang, Yu Liang, Ke Xu, Xue Feng Zhang, Yun Kang, Yu Qin Jin, Jun Wei Hou, Jing Zhang, Tian Yang, Salah Hussein, Mohamed Saif ElDein, Ze Hua Lei, Hao Zhang, Shuai Shao, Zhao Ming Liu, Ning Liu, Xiang Zheng, Ji Guo Su, Sen Sen Yang, Xiangfeng Cong, Yao Tan, Wenwen Lei, Xue Jun Gao, Zhiwei Jiang, Hui Wang, Meng Li, Hanadi Mekki Mekki, Walid Zaher, Sally Mahmoud, Xue Zhang, Chang Qu, Dan Ying Liu, Jing Zhang, Mengjie Yang, Islam Eltantawy, Peng Xiao, Fu Jie Shen, Jin Juan Wu, Zi Bo Han, Li Fang Du, Fang Tang, Shi Chen, Zhi Jing Ma, Fan Zheng, Ya Nan Hou, Xin Yu Li, Xin Li, Zhao Nian Wang, Jin Liang Yin, Xiao Yan Mao, Jin Zhang, Liang Qu, Yun Tao Zhang, Xiao Ming Yang, Guizhen Wu, Qi Ming Li

Correspondence to: Qi Ming Li ([liqiming189@163.com](mailto:liqiming189@163.com)) or Guizhen Wu ([wugz@ivdc.chinacdc.cn](mailto:wugz@ivdc.chinacdc.cn)) or Xiao Ming Yang ([yangxiaoming@sinopharm.com](mailto:yangxiaoming@sinopharm.com)) or Yun Tao Zhang ([zhangyuntao@sinopharm.com](mailto:zhangyuntao@sinopharm.com))

**This PDF file includes:**

Figure. S1

Tables S1 to S47

**Other Supplementary Materials for this manuscript include the following:**

Supplementary Protocol


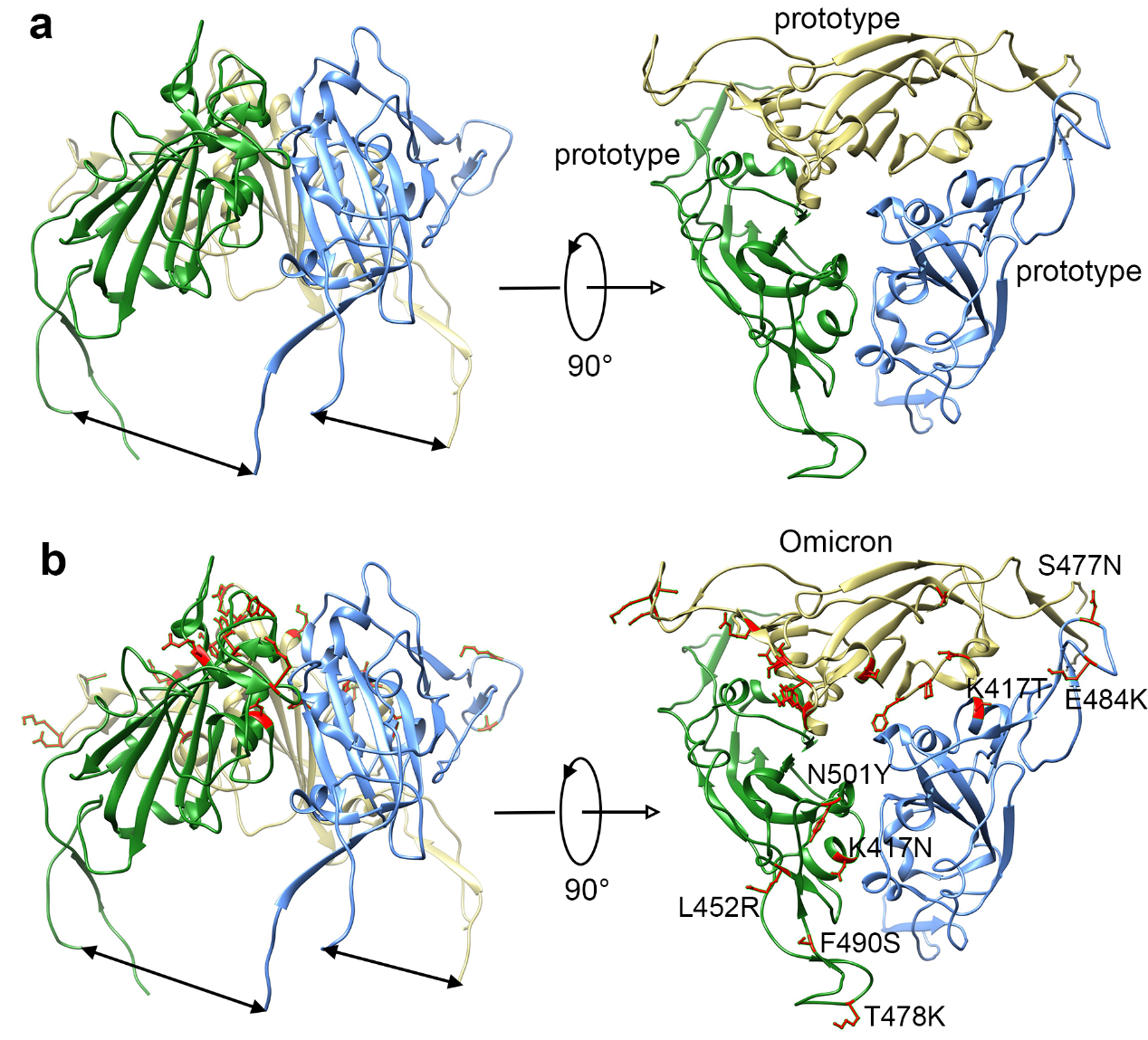


Figure. S1.

Schematic illustration of the designed immunogens for NVSI-06-07 and NVSI-06-09. **a** The trimeric RBD immunogen for NVSI-06-07, in which three homologous RBDs derived from the prototype SARS-CoV-2 strain were connected end-to-end into a single molecule. **b** The mosaic-type trimeric RBD immunogen for NVSI-06-09, which is composed of three heterologous RBDs. One RBD is derived from Omicron BA.1 variant, and the other two are artificially designed harboring the key residues from the immune-evasive SARS-CoV-2 variants. One of the artificially designed RBD carries five mutations of K417N, L452R, T478K, F490S and N50Y, and the other one contains three mutations of K417T, S477N and E484K.

**Table S1: Solicited and unsolicited adverse reactions after booster vaccination**

|  | **NVSI-06-09**  **（N=260）** | **BBIBP-CorV（N=256）** | **Total （N=516）** | ***p* value*** |
| --- | --- | --- | --- | --- |
| **Solicited adverse reactions within 0-7 days** | 31(11.92) | 34(13.28) | 65(12.60) | 0.6915 |
| Grade 1 | 26(10.00) | 30(11.72) | 56(10.85) | 0.5728 |
| Grade 2 | 6(2.31) | 6(2.34) | 12(2.33) | 1.0000 |
| **Injection site adverse reactions** | 15(5.77) | 18(7.03) | 33(6.40) | 0.5932 |
| Grade 1 | 12(4.62) | 18(7.03) | 30(5.81) | 0.2636 |
| Grade 2 | 3(1.15) | 0(0.00) | 3(0.58) | 0.2486 |
| Pain | 15(5.77) | 17(6.64) | 32(6.20) | 0.7181 |
| Grade 1 | 12(4.62) | 17(6.64) | 29(5.62) | 0.3445 |
| Grade 2 | 3(1.15) | 0(0.00) | 3(0.58) | 0.2486 |
| Pruritus | 0(0.00) | 1(0.39) | 1(0.19) | 0.4961 |
| Grade 1 | 0(0.00) | 1(0.39) | 1(0.19) | 0.4961 |
| Induration | 0(0.00) | 0(0.00) | 0(0.00) | 1.0000 |
| Swelling | 0(0.00) | 0(0.00) | 0(0.00) | 1.0000 |
| Rash | 0(0.00) | 0(0.00) | 0(0.00) | 1.0000 |
| Redness | 0(0.00) | 0(0.00) | 0(0.00) | 1.0000 |
| **Systemic adverse reactions** | 20(7.69) | 19(7.42) | 39(7.56) | 1.0000 |
| Grade 1 | 16(6.15) | 14(5.47) | 30(5.81) | 0.8513 |
| Grade 2 | 5(1.92) | 6(2.34) | 11(2.13) | 0.7705 |
| Muscle pain (non-vaccination site) | 11(4.23) | 10(3.91) | 21(4.07) | 1.0000 |
| Grade 1 | 8(3.08) | 5(1.95) | 13(2.52) | 0.5763 |
| Grade 2 | 3(1.15) | 5(1.95) | 8(1.55) | 0.5015 |
| Headache | 7(2.69) | 3(1.17) | 10(1.94) | 0.3391 |
| Grade 1 | 4(1.54) | 3(1.17) | 7(1.36) | 1.0000 |
| Grade 2 | 3(1.15) | 0(0.00) | 3(0.58) | 0.2486 |
| Fever | 3(1.15) | 4(1.56) | 7(1.36) | 0.7229 |
| Grade 1 | 2(0.77) | 4(1.56) | 6(1.16) | 0.4472 |
| Grade 2 | 1(0.38) | 0(0.00) | 1(0.19) | 1.0000 |
| Fatigue | 3(1.15) | 3(1.17) | 6(1.16) | 1.0000 |
| Grade 1 | 3(1.15) | 3(1.17) | 6(1.16) | 1.0000 |
| Pruritus at non-vaccination site (no skin damage) | 1(0.38) | 1(0.39) | 2(0.39) | 1.0000 |
| Grade 1 | 1(0.38) | 0(0.00) | 1(0.19) | 1.0000 |
| Grade 2 | 0(0.00) | 1(0.39) | 1(0.19) | 0.4961 |
| Arthralgia | 0(0.00) | 1(0.39) | 1(0.19) | 0.4961 |
| Grade 1 | 0(0.00) | 1(0.39) | 1(0.19) | 0.4961 |
| Constipation | 1(0.38) | 0(0.00) | 1(0.19) | 1.0000 |
| Grade 1 | 1(0.38) | 0(0.00) | 1(0.19) | 1.0000 |
| Vomiting | 1(0.38) | 0(0.00) | 1(0.19) | 1.0000 |
| Grade 1 | 1(0.38) | 0(0.00) | 1(0.19) | 1.0000 |
| Nausea | 1(0.38) | 0(0.00) | 1(0.19) | 1.0000 |
| Grade 1 | 1(0.38) | 0(0.00) | 1(0.19) | 1.0000 |
| Cough | 1(0.38) | 0(0.00) | 1(0.19) | 1.0000 |
| Grade 1 | 0(0.00) | 0(0.00) | 1(0.19) | 1.0000 |
| Grade 2 | 1(0.38) | 0(0.00) | 1(0.19) | 1.0000 |
| Dyspnea | 1(0.38) | 0(0.00) | 1(0.19) | 1.0000 |
| Grade 1 | 1(0.38) | 0(0.00) | 1(0.19) | 1.0000 |
| Dizziness | 1(0.38) | 0(0.00) | 1(0.19) | 1.0000 |
| Grade 1 | 1(0.38) | 0(0.00) | 1(0.19) | 1.0000 |
| Diarrhea | 0(0.00) | 0(0.00) | 0(0.00) | 1.0000 |
| Dysphagia | 0(0.00) | 0(0.00) | 0(0.00) | 1.0000 |
| Anorexia | 0(0.00) | 0(0.00) | 0(0.00) | 1.0000 |
| Abnormal skin mucosa | 0(0.00) | 0(0.00) | 0(0.00) | 1.0000 |
| Acute allergic reaction | 0(0.00) | 0(0.00) | 0(0.00) | 1.0000 |
| **Unsolicited adverse reactions within 0-30 days** | 8(3.08) | 7(2.73) | 15(2.91) | 1.0000 |
| Grade 1 | 7(2.69) | 3(1.17) | 10(1.94) | 0.3391 |
| Grade 2 | 1(0.38) | 4(1.56) | 5(0.97) | 0.2133 |

Notes: **p*-value was calculated using Fisher's exact test. Data are presented as n (%).

**Table S2: Live-virus neutralizing antibody response against prototype SARS-CoV-2 strain (PPS)**

|  | **14 days after boosting** | | |
| --- | --- | --- | --- |
|  | **NVSI-06-09** | **BBIBP-CorV** | ***p* value** |
| **All subjects** |  |  |  |
| N(missing) | 255(0) | 249(0) |  |
| Pre-booster antibody titer ≥4, n (%)  95%CI (%) | 254(99.61) | 247(99.20) | 0.6198 |
|  | 97.83-99.99 | 97.13-99.90 |  |
| Pre-booster antibody GMT^[1]^ (95%CI) | 119.42(106.47-133.95) | 113.29(100.50-127.71) | 0.5316 |
| Post-booster antibody GMT (95%CI) | 2349.44(2061.42-2677.70) | 506.47(458.25-559.77) | <0.0001 |
| Ratio of GMT between two groups(95%CI)^[2]^ | 4.64(3.93-5.47) |  |  |
| Post-booster adjusted antibody GMT (95%CI)^[3]^ | 1982.55(1601.51-2454.25) | 433.41(349.04-538.18) | <0.0001 |
| Ratio of adjusted GMT between two groups(95%CI)^[3]^ | 4.57(3.91-5.35) |  |  |
| Rate of 4-fold rise^[4]^, n (%) | 238(93.33) | 148(59.44) | <0.0001 |
| 95%CI (%) | 89.54-96.07 | 53.06-65.59 |  |
| Rate difference between two groups (%, 95%CI^[5]^) | 33.95(27.13-40.77) |  |  |
| Post-booster antibody GMT fold rise (95%CI) | 19.67(16.79-23.06) | 4.47(4.01-4.99) | <0.0001 |
| **All subjects (Excluding participants with positive**  **or weak positive COVID-19 PCR test)** |  |  |  |
| N(missing) | 231(0) | 226(0) |  |
| Pre-booster antibody titer ≥4, n (%) | 230(99.57) | 225(99.56) | 1.0000 |
| 95%CI (%) | 97.61-99.99 | 97.56-99.99 |  |
| Pre-booster antibody GMT^[1]^ (95%CI) | 120.32(106.60-135.80) | 115.90(102.63-130.88) | 0.6675 |
| Post-booster antibody GMT (95%CI) | 2321.93(2020.94-2667.74) | 514.35(462.45-572.08) | <0.0001 |
| Ratio of GMT between two groups(95%CI)^[2]^ | 4.51(3.79-5.38) |  |  |
| Post-booster adjusted antibody GMT (95%CI)^[3]^ | 1867.43(1490.04-2340.41) | 415.29(329.12-524.02) | <0.0001 |
| Ratio of adjusted GMT between two groups(95%CI)^[3]^ | 4.50(3.81-5.31) |  |  |
| Rate of 4-fold rise^[4]^, n (%) | 214(92.64) | 134(59.29) | <0.0001 |
| 95%CI (%) | 88.48-95.65 | 52.58-65.76 |  |
| Rate difference between two groups (%, 95%CI^[5]^) | 33.57(26.34-40.80) |  |  |
| Post-booster antibody GMT fold rise (95%CI) | 19.30(16.31-22.83) | 4.44(3.96-4.98) | <0.0001 |
| **Subjects who have received 2 doses of inactivated vaccine previously** |  |  |  |
| N(missing) | 11(0) | 9(0) |  |
| Pre-booster antibody titer ≥4, n (%) | 11(100.00) | 9(100.00) | 1.0000 |
| 95%CI (%) | 71.51-100.00 | 66.37-100.00 |  |
| Pre-booster antibody GMT^[1]^ (95%CI) | 83.73(44.50-157.55) | 183.54(119.77-281.26) | 0.0410 |
| Post-booster antibody GMT (95%CI) | 1277.47(682.73-2390.30) | 509.04(298.48-868.14) | 0.0247 |
| Ratio of GMT between two groups(95%CI)^[2]^ | 2.51(1.14-5.52) |  |  |
| Post-booster adjusted antibody GMT (95%CI)^[3]^ | 1271.04(712.48-2267.47) | 512.19(268.04-978.74) | 0.0521 |
| Ratio of adjusted GMT between two groups(95%CI)^[3]^ | 2.48(0.99-6.22) |  |  |
| Rate of 4-fold rise^[4]^, n (%) | 10(90.91) | 4(44.44) | 0.0279 |
| 95%CI (%) | 58.72-99.77 | 13.70-78.80 |  |
| Rate difference between two groups (%, 95%CI^[5]^) | 46.46(9.82-83.10) |  |  |
| Post-booster antibody GMT fold rise (95%CI) | 15.26(5.84-39.85) | 2.77(1.64-4.70) | 0.0042 |
| **Subjects who have received 3 doses of inactivated vaccine previously** |  |  |  |
| N(missing) | 244(0) | 240(0) |  |
| Pre-booster antibody titer ≥4, n (%) | 243(99.59) | 238(99.17) | 0.6211 |
| 95%CI (%) | 97.74-99.99 | 97.02-99.90 |  |
| Pre-booster antibody GMT^[1]^ (95%CI) | 121.35(107.93-136.44) | 111.26(98.38-125.82) | 0.3145 |
| Post-booster antibody GMT (95%CI) | 2414.87(2112.91-2759.98) | 506.38(457.01-561.08) | <0.0001 |
| Ratio of GMT between two groups(95%CI)^[2]^ | 4.77(4.03-5.64) |  |  |
| Post-booster adjusted antibody GMT (95%CI)^[3]^ | 2382.06(2128.58-2665.72) | 513.47(458.40-575.15) | <0.0001 |
| Ratio of adjusted GMT between two groups(95%CI)^[3]^ | 4.64(3.95-5.44) |  |  |
| Rate of 4-fold rise^[4]^, n (%) | 228(93.44) | 144(60.00) | <0.0001 |
| 95%CI (%) | 89.57-96.21 | 53.50-66.25 |  |
| Rate difference between two groups (%, 95%CI^[5]^) | 33.44(26.51-40.38) |  |  |
| Post-booster antibody GMT fold rise (95%CI) | 19.90(16.93-23.39) | 4.55(4.07-5.09) | <0.0001 |

Notes: [1] GMT represent geometric mean titer.

[2] The ratio of GMT between two groups was calculated by “NVSI-06-09/BBIBP-CorV”.

[3] Covariance analysis with least square method was used to calculate the adjusted GMT and the corresponding *p* value.

[4] Rate of 4-fold rise was defined as percentage of participants with a ≥4-fold rise in neutralizing antibody titer from baseline.

[5] Rate difference=(NVSI-06-09)-(BBIBP-CorV). Rate difference and 95%CI were estimated by CMH method considering stratification factors.

**Table S3:** **Comparison of the neutralizing antibody response against prototype SARS-CoV-2 strain elicited by NVSI-06-09 booster between subjects primed with two and three doses of inactivated vaccine (PPS)**

|  | **NVSI-06-09** | | |
| --- | --- | --- | --- |
|  | **2 doses** | **3 doses** | ***p* value** |
| **All subjects** |  |  |  |
| N(missing) | 11(0) | 244(0) |  |
| Pre-booster antibody titer ≥4, n (%) | 11(100.00) | 243(99.59) | 1.0000 |
| 95%CI (%) | 71.51-100.00 | 97.74-99.99 |  |
| Pre-booster antibody GMT^[1]^ (95%CI) | 83.73(44.50-157.55) | 121.35(107.93-136.44) | 0.1966 |
| Post-booster antibody GMT (95%CI) | 1277.47(682.73-2390.30) | 2414.87(2112.91-2759.98) | 0.0512 |
| Ratio of GMT between two groups(95%CI)^[2]^ | 0.53(0.28-1.00) |  |  |
| Post-booster adjusted antibody GMT (95%CI)^[3]^ | 1363.21(732.46-2537.15) | 2407.81(2111.12-2746.19) | 0.0790 |
| Ratio of adjusted GMT between two groups(95%CI)^[3]^ | 0.57(0.30-1.07) |  |  |
| Rate of 4-fold rise^[4]^, n (%) | 10(90.91) | 228(93.44) | 0.7423 |
| 95%CI (%) | 58.72-99.77 | 89.57-96.21 |  |
| Rate difference between two groups (%, 95%CI^[5]^) | -2.53(-19.80-14.74) |  |  |
| Post-booster antibody GMT fold rise (95%CI) | 15.26(5.84-39.85) | 19.90(16.93-23.39) | 0.5041 |
| **All subjects (Excluding participants with positive**  **or weak positive COVID-19 PCR test)** |  |  |  |
| N(missing) | 11(0) | 220(0) |  |
| Pre-booster antibody titer ≥4, n (%) | 11(100.00) | 219(99.55) | 1.0000 |
| 95%CI (%) | 71.51-100.00 | 97.49-99.99 |  |
| Pre-booster antibody GMT^[1]^ (95%CI) | 83.73(44.50-157.55) | 122.52(108.25-138.67) | 0.1876 |
| Post-booster antibody GMT (95%CI) | 1277.47(682.73-2390.30) | 2392.35(2075.15-2758.03) | 0.0578 |
| Ratio of GMT between two groups(95%CI)^[2]^ | 0.53(0.28-1.02) |  |  |
| Post-booster adjusted antibody GMT (95%CI)^[3]^ | 1364.10(727.76-2556.85) | 2384.51(2072.98-2742.85) | 0.0888 |
| Ratio of adjusted GMT between two groups(95%CI)^[3]^ | 0.57(0.30-1.09) |  |  |
| Rate of 4-fold rise^[4]^, n (%) | 10(90.91) | 204(92.73) | 0.8221 |
| 95%CI (%) | 58.72-99.77 | 88.46-95.79 |  |
| Rate difference between two groups (%, 95%CI^[5]^) | -1.82(-19.15-15.51) |  |  |
| Post-booster antibody GMT fold rise (95%CI) | 15.26(5.84-39.85) | 19.53(16.44-23.19) | 0.5394 |

Notes: [1] GMT represent geometric mean titer.

[2] The ratio of GMT between two groups was calculated by “NVSI-06-09/ BBIBP-CorV”.

[3] Covariance analysis with least square method was used to calculate the adjusted GMT and the corresponding *p* value.

[4] Rate of 4-fold rise was defined as percentage of participants with a ≥4-fold rise in neutralizing antibody titer from baseline.

[5] Rate difference=(NVSI-06-09)-(BBIBP-CorV). Rate difference and 95%CI were estimated by CMH method considering stratification factors.

**Table S4: Comparison of the neutralizing antibody response against prototype SARS-CoV-2 strain elicited by BBIBP-CorV booster between subjects primed with two and three doses of inactivated vaccine (PPS)**

|  | **BBIBP-CorV** | | |
| --- | --- | --- | --- |
|  | **2 doses** | **3 doses** | ***p* value** |
| **All subjects** |  |  |  |
| N(missing) | 9(0) | 240(0) |  |
| Pre-booster antibody titer ≥4, n (%) | 9(100.00) | 238(99.17) | 1.0000 |
| 95%CI (%) | 66.37-100.00 | 97.02-99.90 |  |
| Pre-booster antibody GMT^[1]^ (95%CI) | 183.54(119.77-281.26) | 111.26(98.38-125.82) | 0.1247 |
| Post-booster antibody GMT (95%CI) | 509.04(298.48-868.14) | 506.38(457.01-561.08) | 0.9846 |
| Ratio of GMT between two groups(95%CI)^[2]^ | 1.01(0.59-1.72) |  |  |
| Post-booster adjusted antibody GMT (95%CI)^[3]^ | 413.37(262.16-651.78) | 510.35(467.45-557.18) | 0.3718 |
| Ratio of adjusted GMT between two groups(95%CI)^[3]^ | 0.81(0.51-1.29) |  |  |
| Rate of 4-fold rise^[4]^, n (%) | 4(44.44) | 144(60.00) | 0.3517 |
| 95%CI (%) | 13.70-78.80 | 53.50-66.25 |  |
| Rate difference between two groups (%, 95%CI^[5]^) | -15.56(-48.61-17.49) |  |  |
| Post-booster antibody GMT fold rise (95%CI) | 2.77(1.64-4.70) | 4.55(4.07-5.09) | 0.0978 |
| **All subjects (Excluding participants with positive**  **or weak positive COVID-19 PCR test)** |  |  |  |
| N(missing) | 7(0) | 219(0) |  |
| Pre-booster antibody titer ≥4, n (%) | 7(100.00) | 218(99.54) | 1.0000 |
| 95%CI (%) | 59.04-100.00 | 97.48-99.99 |  |
| Pre-booster antibody GMT^[1]^ (95%CI) | 173.90(97.68-309.59) | 114.40(101.03-129.55) | 0.2404 |
| Post-booster antibody GMT (95%CI) | 393.43(240.98-642.33) | 518.78(465.22-578.51) | 0.3759 |
| Ratio of GMT between two groups(95%CI)^[2]^ | 0.76(0.41-1.40) |  |  |
| Post-booster adjusted antibody GMT (95%CI)^[3]^ | 328.18(194.55-553.58) | 521.80(475.36-572.77) | 0.0868 |
| Ratio of adjusted GMT between two groups(95%CI)^[3]^ | 0.63(0.37-1.07) |  |  |
| Rate of 4-fold rise^[4]^, n (%) | 2(28.57) | 132(60.27) | 0.0936 |
| 95%CI (%) | 3.67-70.96 | 53.46-66.80 |  |
| Rate difference between two groups (%, 95%CI^[5]^) | -31.70(-65.79-2.38) |  |  |
| Post-booster antibody GMT fold rise (95%CI) | 2.26(1.29-3.97) | 4.53(4.04-5.09) | 0.0377 |

Notes: [1] GMT represent geometric mean titer.

[2] The ratio of GMT between two groups was calculated by “NVSI-06-09/ BBIBP-CorV”.

[3] Covariance analysis with least square method was used to calculate the adjusted GMT and the corresponding *p* value.

[4] Rate of 4-fold rise was defined as percentage of participants with a ≥4-fold rise in neutralizing antibody titer from baseline.

[5] Rate difference=(NVSI-06-09)-(BBIBP-CorV). Rate difference and 95%CI were estimated by CMH method considering stratification factors.

**Table S5: Live-virus neutralizing antibody response against Omicron (BA.1.1) variant (PPS)**

|  | **14 days after boosting** | | |
| --- | --- | --- | --- |
|  | **NVSI-06-09** | **BBIBP-CorV** | ***p* value** |
| **All subjects** |  |  |  |
| N(missing) | 255(0) | 249(0) |  |
| Pre-booster antibody titer ≥4, n (%) | 247(96.86) | 242(97.19) | 0.8295 |
| 95%CI (%) | 93.91-98.64 | 94.29-98.86 |  |
| Pre-booster antibody GMT^[1]^ (95%CI) | 40.47(34.86-46.97) | 37.91(32.64-44.04) | 0.5441 |
| Post-booster antibody GMT (95%CI) | 1713.71(1447.12-2029.41) | 143.23(124.48-164.80) | <0.0001 |
| Ratio of GMT between two groups(95%CI)^[2]^ | 11.96(9.60-14.90) |  |  |
| Post-booster adjusted antibody GMT (95%CI)^[3]^ | 1321.99(1019.80-1713.73) | 113.91(87.56-148.18) | <0.0001 |
| Ratio of adjusted GMT between two groups(95%CI)^[3]^ | 11.61(9.60-14.03) |  |  |
| Rate of 4-fold rise^[4]^, n (%) | 247(96.86) | 134(53.82) | <0.0001 |
| 95%CI (%) | 93.91-98.64 | 47.41-60.13 |  |
| Rate difference between two groups (%, 95%CI^[5]^) | 43.21(36.69-49.73) |  |  |
| Post-booster antibody GMT fold rise (95%CI) | 42.35(35.15-51.03) | 3.78(3.40-4.20) | <0.0001 |
| **All subjects (Excluding participants with positive**  **or weak positive COVID-19 PCR test)** |  |  |  |
| N(missing) | 231(0) | 226(0) |  |
| Pre-booster antibody titer ≥4, n (%) | 224(96.97) | 220(97.35) | 0.8093 |
| 95%CI (%) | 93.86-98.77 | 94.31-99.02 |  |
| Pre-booster antibody GMT^[1]^ (95%CI) | 41.67(35.69-48.65) | 38.41(32.81-44.97) | 0.4685 |
| Post-booster antibody GMT (95%CI) | 1732.14(1449.09-2070.47) | 149.81(129.37-173.48) | <0.0001 |
| Ratio of GMT between two groups(95%CI)^[2]^ | 11.56(9.18-14.57) |  |  |
| Post-booster adjusted antibody GMT (95%CI)^[3]^ | 1319.09(1003.59-1733.77) | 117.89(88.98-156.18) | <0.0001 |
| Ratio of adjusted GMT between two groups(95%CI)^[3]^ | 11.19(9.15-13.68) |  |  |
| Rate of 4-fold rise^[4]^, n (%) | 223(96.54) | 124(54.87) | <0.0001 |
| 95%CI (%) | 93.29-98.49 | 48.13-61.47 |  |
| Rate difference between two groups (%, 95%CI^[5]^) | 42.10(35.25-48.95) |  |  |
| Post-booster antibody GMT fold rise (95%CI) | 41.57(34.10-50.67) | 3.90(3.49-4.36) | <0.0001 |
| **Subjects who have received 2 doses of inactivated vaccine previously** |  |  |  |
| N(missing) | 11(0) | 9(0) |  |
| Pre-booster antibody titer ≥4, n (%) | 11(100.00) | 9(100.00) | 1.0000 |
| 95%CI (%) | 71.51-100.00 | 66.37-100.00 |  |
| Pre-booster antibody GMT^[1]^ (95%CI) | 41.42(18.48-92.84) | 73.26(43.71-122.78) | 0.2212 |
| Post-booster antibody GMT (95%CI) | 1138.33(502.74-2577.48) | 109.09(53.40-222.87) | 0.0002 |
| Ratio of GMT between two groups(95%CI)^[2]^ | 10.43(3.70-29.43) |  |  |
| Post-booster adjusted antibody GMT (95%CI)^[3]^ | 1122.91(539.91-2335.46) | 110.93(49.20-250.11) | 0.0004 |
| Ratio of adjusted GMT between two groups(95%CI)^[3]^ | 10.12(3.31-30.93) |  |  |
| Rate of 4-fold rise^[4]^, n (%) | 10(90.91) | 1(11.11) | 0.0005 |
| 95%CI (%) | 58.72-99.77 | 0.28-48.25 |  |
| Rate difference between two groups (%, 95%CI^[5]^) | 79.80(53.15-100.00) |  |  |
| Post-booster antibody GMT fold rise (95%CI) | 27.48(7.32-103.15) | 1.49(0.94-2.37) | 0.0005 |
| **Subjects who have received 3 doses of inactivated vaccine previously** |  |  |  |
| N(missing) | 244(0) | 240(0) |  |
| Pre-booster antibody titer ≥4, n (%) | 236(96.72) | 233(97.08) | 0.8183 |
| 95%CI (%) | 93.64-98.57 | 94.08-98.82 |  |
| Pre-booster antibody GMT^[1]^ (95%CI) | 40.42(34.69-47.10) | 36.99(31.72-43.14) | 0.4202 |
| Post-booster antibody GMT (95%CI) | 1745.61(1467.36-2076.62) | 144.70(125.32-167.08) | <0.0001 |
| Ratio of GMT between two groups(95%CI)^[2]^ | 12.06(9.63-15.11) |  |  |
| Post-booster adjusted antibody GMT (95%CI)^[3]^ | 1704.55(1488.14-1952.43) | 148.24(129.28-169.99) | <0.0001 |
| Ratio of adjusted GMT between two groups(95%CI)^[3]^ | 11.50(9.48-13.94) |  |  |
| Rate of 4-fold rise^[4]^, n (%) | 237(97.13) | 133(55.42) | <0.0001 |
| 95%CI (%) | 94.18-98.84 | 48.89-61.81 |  |
| Rate difference between two groups (%, 95%CI^[5]^) | 41.71(35.09-48.34) |  |  |
| Post-booster antibody GMT fold rise (95%CI) | 43.18(35.78-52.11) | 3.91(3.51-4.35) | <0.0001 |

Notes: [1] GMT represent geometric mean titer.

[2] The ratio of GMT between two groups was calculated by “NVSI-06-09/ BBIBP-CorV”.

[3] Covariance analysis with least square method was used to calculate the adjusted GMT and the corresponding *p* value.

[4] Rate of 4-fold rise was defined as percentage of participants with a ≥4-fold rise in neutralizing antibody titer from baseline.

[5] Rate difference=(NVSI-06-09)-(BBIBP-CorV). Rate difference and 95%CI were estimated by CMH method considering stratification factors.

**Table S6:** **Comparison of the neutralizing antibody response against Omicron (BA.1.1) variant elicited by NVSI-06-09 booster between subjects primed with two and three doses of inactivated vaccine (PPS)**

|  | **NVSI-06-09** | | |
| --- | --- | --- | --- |
|  | **2 doses** | **3 doses** | ***p* value** |
| **All subjects** |  |  |  |
| N(missing) | 11(0) | 244(0) |  |
| Pre-booster antibody titer ≥4, n (%) | 11(100.00) | 236(96.72) | 1.0000 |
| 95%CI (%) | 71.51-100.00 | 93.64-98.57 |  |
| Pre-booster antibody GMT^[1]^ (95%CI) | 41.42(18.48-92.84) | 40.42(34.69-47.10) | 0.9481 |
| Post-booster antibody GMT (95%CI) | 1138.33(502.74-2577.48) | 1745.61(1467.36-2076.62) | 0.3126 |
| Ratio of GMT between two groups(95%CI)^[2]^ | 0.65(0.28-1.50) |  |  |
| Post-booster adjusted antibody GMT (95%CI)^[3]^ | 1128.78(521.07-2445.23) | 1746.27(1481.94-2057.74) | 0.2779 |
| Ratio of adjusted GMT between two groups(95%CI)^[3]^ | 0.65(0.29-1.42) |  |  |
| Rate of 4-fold rise^[4]^, n (%) | 10(90.91) | 237(97.13) | 0.2478 |
| 95%CI (%) | 58.72-99.77 | 94.18-98.84 |  |
| Rate difference between two groups (%, 95%CI^[5]^) | -6.22(-23.34-10.90) |  |  |
| Post-booster antibody GMT fold rise (95%CI) | 27.48(7.32-103.15) | 43.18(35.78-52.11) | 0.3332 |
| **All subjects (Excluding participants with positive**  **or weak positive COVID-19 PCR test)** |  |  |  |
| N(missing) | 11(0) | 220(0) |  |
| Pre-booster antibody titer ≥4, n (%) | 11(100.00) | 213(96.82) | 1.0000 |
| 95%CI (%) | 71.51-100.00 | 93.55-98.71 |  |
| Pre-booster antibody GMT^[1]^ (95%CI) | 41.42(18.48-92.84) | 41.68(35.55-48.86) | 0.9864 |
| Post-booster antibody GMT (95%CI) | 1138.33(502.74-2577.48) | 1768.88(1471.96-2125.69) | 0.3009 |
| Ratio of GMT between two groups(95%CI)^[2]^ | 0.64(0.28-1.49) |  |  |
| Post-booster adjusted antibody GMT (95%CI)^[3]^ | 1140.70(522.23-2491.60) | 1768.69(1485.19-2106.32) | 0.2815 |
| Ratio of adjusted GMT between two groups(95%CI)^[3]^ | 0.64(0.29-1.44) |  |  |
| Rate of 4-fold rise^[4]^, n (%) | 10(90.91) | 213(96.82) | 0.2966 |
| 95%CI (%) | 58.72-99.77 | 93.55-98.71 |  |
| Rate difference between two groups (%, 95%CI^[5]^) | -5.91(-23.06-11.24) |  |  |
| Post-booster antibody GMT fold rise (95%CI) | 27.48(7.32-103.15) | 42.44(34.75-51.83) | 0.3583 |

Notes: [1] GMT represent geometric mean titer.

[2] The ratio of GMT between two groups was calculated by “NVSI-06-09/ BBIBP-CorV”.

[3] Covariance analysis with least square method was used to calculate the adjusted GMT and the corresponding *p* value.

[4] Rate of 4-fold rise was defined as percentage of participants with a ≥4-fold rise in neutralizing antibody titer from baseline.

[5] Rate difference=(NVSI-06-09)-(BBIBP-CorV). Rate difference and 95%CI were estimated by CMH method considering stratification factors.

**Table S7:** **Comparison of the neutralizing antibody response against Omicron (BA.1.1) variant elicited by BBIBP-CorV booster between subjects primed with two and three doses of inactivated vaccine (PPS)**

|  | **BBIBP-CorV** | | |
| --- | --- | --- | --- |
|  | **2 doses** | **3 doses** | ***p* value** |
| **All subjects** |  |  |  |
| N(missing) | 9(0) | 240(0) |  |
| Pre-booster antibody titer ≥4, n (%) | 9(100.00) | 233(97.08) | 1.0000 |
| 95%CI (%) | 66.37-100.00 | 94.08-98.82 |  |
| Pre-booster antibody GMT^[1]^ (95%CI) | 73.26(43.71-122.78) | 36.99(31.72-43.14) | 0.0935 |
| Post-booster antibody GMT (95%CI) | 109.09(53.40-222.87) | 144.70(125.32-167.08) | 0.4603 |
| Ratio of GMT between two groups(95%CI)^[2]^ | 0.75(0.36-1.60) |  |  |
| Post-booster adjusted antibody GMT (95%CI)^[3]^ | 68.85(41.82-113.32) | 147.22(133.74-162.05) | 0.0035 |
| Ratio of adjusted GMT between two groups(95%CI)^[3]^ | 0.47(0.28-0.78) |  |  |
| Rate of 4-fold rise^[4]^, n (%) | 1(11.11) | 133(55.42) | 0.0090 |
| 95%CI (%) | 0.28-48.25 | 48.89-61.81 |  |
| Rate difference between two groups (%, 95%CI^[5]^) | -44.31(-65.78--22.83) |  |  |
| Post-booster antibody GMT fold rise (95%CI) | 1.49(0.94-2.37) | 3.91(3.51-4.35) | 0.0008 |
| **All subjects (Excluding participants with positive**  **or weak positive COVID-19 PCR test)** |  |  |  |
| N(missing) | 7(0) | 219(0) |  |
| Pre-booster antibody titer ≥4, n (%) | 7(100.00) | 213(97.26) | 1.0000 |
| 95%CI (%) | 59.04-100.00 | 94.13-98.99 |  |
| Pre-booster antibody GMT^[1]^ (95%CI) | 76.15(37.33-155.30) | 37.58(31.99-44.15) | 0.1266 |
| Post-booster antibody GMT (95%CI) | 104.22(41.71-260.44) | 151.55(130.50-176.00) | 0.3848 |
| Ratio of GMT between two groups(95%CI)^[2]^ | 0.69(0.29-1.60) |  |  |
| Post-booster adjusted antibody GMT (95%CI)^[3]^ | 64.84(36.95-113.78) | 153.87(139.22-170.06) | 0.0032 |
| Ratio of adjusted GMT between two groups(95%CI)^[3]^ | 0.42(0.24-0.75) |  |  |
| Rate of 4-fold rise^[4]^, n (%) | 0(0.00) | 124(56.62) | 0.0031 |
| 95%CI (%) | 59.04-100.00 | 36.72-50.22 |  |
| Rate difference between two groups (%, 95%CI^[5]^) | 56.62(50.06-63.18) |  |  |
| Post-booster antibody GMT fold rise (95%CI) | 1.37(0.83-2.25) | 4.03(3.61-4.51) | 0.0009 |

Notes: [1] GMT represent geometric mean titer.

[2] The ratio of GMT between two groups was calculated by “NVSI-06-09/ BBIBP-CorV”.

[3] Covariance analysis with least square method was used to calculate the adjusted GMT and the corresponding *p* value.

[4] Rate of 4-fold rise was defined as percentage of participants with a ≥4-fold rise in neutralizing antibody titer from baseline.

[5] Rate difference=(NVSI-06-09)-(BBIBP-CorV). Rate difference and 95%CI were estimated by CMH method considering stratification factors.

**Table S8:** **Comparison of the neutralizing antibody response against prototype SARS-CoV-2 strain elicited by NVSI-06-09 booster between male and female subjects (PPS)**

|  | **NVSI-06-09** | | |
| --- | --- | --- | --- |
|  | **Male** | **Female** | ***p* value** |
| **All subjects** |  |  |  |
| N(missing) | 239(0) | 16(0) |  |
| Pre-booster antibody titer ≥4, n (%) | 238(99.58) | 16(100.00) | 1.0000 |
| 95%CI (%) | 97.69-99.99 | 79.41-100.00 |  |
| Pre-booster antibody GMT^[1]^ (95%CI) | 120.95(107.39-136.22) | 98.79(60.94-160.14) | 0.4009 |
| Post-booster antibody GMT (95%CI) | 2360.78(2059.96-2705.52) | 2186.43(1325.14-3607.51) | 0.7800 |
| Ratio of GMT between two groups(95%CI)^[2]^ | 1.08(0.63-1.85) |  |  |
| Post-booster adjusted antibody GMT (95%CI)^[3]^ | 1809.57(1308.52-2502.48) | 1828.00(1034.71-3229.51) | 0.9703 |
| Ratio of adjusted GMT between two groups(95%CI)^[3]^ | 0.99(0.58-1.69) |  |  |
| Rate of 4-fold rise^[4]^, n (%) | 223(93.31) | 15(93.75) | 0.9189 |
| 95%CI (%) | 89.36-96.13 | 69.77-99.84 |  |
| Rate difference between two groups (%, 95%CI^[5]^) | -0.67(-13.16-11.82) |  |  |
| Post-booster antibody GMT fold rise (95%CI) | 19.52(16.57-23.00) | 22.13(10.90-44.96) | 0.7061 |
| **All subjects (Excluding participants with positive**  **or weak positive COVID-19 PCR test)** |  |  |  |
| N(missing) | 216(0) | 15(0) |  |
| Pre-booster antibody titer ≥4, n (%) | 215(99.54) | 15(100.00) | 1.0000 |
| 95%CI (%) | 97.45-99.99 | 78.20-100.00 |  |
| Pre-booster antibody GMT^[1]^ (95%CI) | 121.18(106.82-137.47) | 108.56(67.68-174.13) | 0.6601 |
| Post-booster antibody GMT (95%CI) | 2308.61(1994.69-2671.92) | 2522.52(1645.18-3867.73) | 0.7574 |
| Ratio of GMT between two groups(95%CI)^[2]^ | 0.92(0.52-1.61) |  |  |
| Post-booster adjusted antibody GMT (95%CI)^[3]^ | 1769.02(1272.98-2458.35) | 2079.29(1155.05-3743.11) | 0.5691 |
| Ratio of adjusted GMT between two groups(95%CI)^[3]^ | 0.85(0.49-1.49) |  |  |
| Rate of 4-fold rise^[4]^, n (%) | 200(92.59) | 14(93.33) | 0.8976 |
| 95%CI (%) | 88.25-95.71 | 68.05-99.83 |  |
| Rate difference between two groups (%, 95%CI^[5]^) | -0.92(-14.25-12.41) |  |  |
| Post-booster antibody GMT fold rise (95%CI) | 19.05(16.01-22.67) | 23.24(10.93-49.39) | 0.5676 |

Notes: [1] GMT represent geometric mean titer.

[2] The ratio of GMT between two groups was calculated by “NVSI-06-09/ BBIBP-CorV”.

[3] Covariance analysis with least square method was used to calculate the adjusted GMT and the corresponding *p* value.

[4] Rate of 4-fold rise was defined as percentage of participants with a ≥4-fold rise in neutralizing antibody titer from baseline.

[5] Rate difference=(NVSI-06-09)-(BBIBP-CorV). Rate difference and 95%CI were estimated by CMH method considering stratification factors.

**Table S9: Comparison of the neutralizing antibody response against prototype SARS-CoV-2 strain elicited by BBIBP-CorV booster between male and female subjects (PPS)**

|  | **BBIBP-CorV** | | |
| --- | --- | --- | --- |
|  | **Male** | **Female** | ***p* value** |
| **All subjects** |  |  |  |
| N(missing) | 237(0) | 12(0) |  |
| Pre-booster antibody titer ≥4, n (%) | 236(99.58) | 11(91.67) | 0.0942 |
| 95%CI (%) | 97.67-99.99 | 61.52-99.79 |  |
| Pre-booster antibody GMT^[1]^ (95%CI) | 114.89(102.06-129.32) | 85.90(32.60-226.33) | 0.3068 |
| Post-booster antibody GMT (95%CI) | 508.91(458.88-564.40) | 460.64(301.21-704.46) | 0.6753 |
| Ratio of GMT between two groups(95%CI)^[2]^ | 1.10(0.69-1.76) |  |  |
| Post-booster adjusted antibody GMT (95%CI)^[3]^ | 459.07(363.82-579.27) | 467.37(296.09-737.73) | 0.9305 |
| Ratio of adjusted GMT between two groups(95%CI)^[3]^ | 0.98(0.66-1.47) |  |  |
| Rate of 4-fold rise^[4]^, n (%) | 141(59.49) | 7(58.33) | 0.9040 |
| 95%CI (%) | 52.95-65.80 | 27.67-84.83 |  |
| Rate difference between two groups (%, 95%CI^[5]^) | 1.75(-26.85-30.36) |  |  |
| Post-booster antibody GMT fold rise (95%CI) | 4.43(3.97-4.94) | 5.36(2.37-12.15) | 0.4646 |
| **All subjects (Excluding participants with positive**  **or weak positive COVID-19 PCR test)** |  |  |  |
| N(missing) | 216(0) | 10(0) |  |
| Pre-booster antibody titer ≥4, n (%) | 215(99.54) | 10(100.00) | 1.0000 |
| 95%CI (%) | 97.45-99.99 | 69.15-100.00 |  |
| Pre-booster antibody GMT^[1]^ (95%CI) | 115.18(101.73-130.39) | 132.60(64.30-273.46) | 0.6396 |
| Post-booster antibody GMT (95%CI) | 512.54(458.99-572.32) | 555.25(378.47-814.60) | 0.7611 |
| Ratio of GMT between two groups(95%CI)^[2]^ | 0.92(0.55-1.55) |  |  |
| Post-booster adjusted antibody GMT (95%CI)^[3]^ | 413.80(317.06-540.04) | 414.65(248.33-692.35) | 0.9928 |
| Ratio of adjusted GMT between two groups(95%CI)^[3]^ | 1.00(0.64-1.56) |  |  |
| Rate of 4-fold rise^[4]^, n (%) | 128(59.26) | 6(60.00) | 0.9856 |
| 95%CI (%) | 52.39-65.88 | 26.24-87.84 |  |
| Rate difference between two groups (%, 95%CI^[5]^) | 0.29(-30.79-31.37) |  |  |
| Post-booster antibody GMT fold rise (95%CI) | 4.45(3.96-5.00) | 4.19(2.22-7.89) | 0.8299 |

Notes: [1] GMT represent geometric mean titer.

[2] The ratio of GMT between two groups was calculated by “NVSI-06-09/ BBIBP-CorV”.

[3] Covariance analysis with least square method was used to calculate the adjusted GMT and the corresponding *p* value.

[4] Rate of 4-fold rise was defined as percentage of participants with a ≥4-fold rise in neutralizing antibody titer from baseline.

[5] Rate difference=(NVSI-06-09)-(BBIBP-CorV). Rate difference and 95%CI were estimated by CMH method considering stratification factors.

**Table S10:** **Comparison of the neutralizing antibody response against Omicron (BA.1.1) variant elicited by NVSI-06-09 booster between male and female subjects (PPS)**

|  | **NVSI-06-09** | | |
| --- | --- | --- | --- |
|  | **Male** | **Female** | ***p* value** |
| **All subjects** |  |  |  |
| N(missing) | 239(0) | 16(0) |  |
| Pre-booster antibody titer ≥4, n (%) | 231(96.65) | 16(100.00) | 1.0000 |
| 95%CI (%) | 93.51-98.54 | 79.41-100.00 |  |
| Pre-booster antibody GMT^[1]^ (95%CI) | 41.75(35.82-48.67) | 25.33(12.98-49.41) | 0.1097 |
| Post-booster antibody GMT (95%CI) | 1707.71(1431.54-2037.16) | 1805.80(956.94-3407.64) | 0.8750 |
| Ratio of GMT between two groups(95%CI)^[2]^ | 0.95(0.47-1.90) |  |  |
| Post-booster adjusted antibody GMT (95%CI)^[3]^ | 1357.58(906.90-2032.24) | 1797.06(883.81-3653.99) | 0.4102 |
| Ratio of adjusted GMT between two groups(95%CI)^[3]^ | 0.76(0.39-1.48) |  |  |
| Rate of 4-fold rise^[4]^, n (%) | 232(97.07) | 15(93.75) | 0.5628 |
| 95%CI (%) | 94.06-98.81 | 69.77-99.84 |  |
| Rate difference between two groups (%, 95%CI^[5]^) | 2.81(-9.48-15.10) |  |  |
| Post-booster antibody GMT fold rise (95%CI) | 40.90(33.72-49.60) | 71.29(33.41-152.10) | 0.1551 |
| **All subjects (Excluding participants with positive**  **or weak positive COVID-19 PCR test)** |  |  |  |
| N(missing) | 216(0) | 15(0) |  |
| Pre-booster antibody titer ≥4, n (%) | 209(96.76) | 15(100.00) | 1.0000 |
| 95%CI (%) | 93.44-98.69 | 78.20-100.00 |  |
| Pre-booster antibody GMT^[1]^ (95%CI) | 42.77(36.45-50.19) | 28.65(14.79-55.49) | 0.2095 |
| Post-booster antibody GMT (95%CI) | 1703.83(1411.77-2056.31) | 2195.98(1311.83-3676.06) | 0.4911 |
| Ratio of GMT between two groups(95%CI)^[2]^ | 0.78(0.38-1.60) |  |  |
| Post-booster adjusted antibody GMT (95%CI)^[3]^ | 1347.00(895.24-2026.72) | 2092.91(1006.34-4352.68) | 0.2144 |
| Ratio of adjusted GMT between two groups(95%CI)^[3]^ | 0.64(0.32-1.29) |  |  |
| Rate of 4-fold rise^[4]^, n (%) | 209(96.76) | 14(93.33) | 0.5775 |
| 95%CI (%) | 93.44-98.69 | 68.05-99.83 |  |
| Rate difference between two groups (%, 95%CI^[5]^) | 2.92(-10.19-16.02) |  |  |
| Post-booster antibody GMT fold rise (95%CI) | 39.84(32.46-48.89) | 76.66(34.51-170.27) | 0.1087 |

Notes: [1] GMT represent geometric mean titer.

[2] The ratio of GMT between two groups was calculated by “NVSI-06-09/ BBIBP-CorV”.

[3] Covariance analysis with least square method was used to calculate the adjusted GMT and the corresponding *p* value.

[4] Rate of 4-fold rise was defined as percentage of participants with a ≥4-fold rise in neutralizing antibody titer from baseline.

[5] Rate difference=(NVSI-06-09)-(BBIBP-CorV). Rate difference and 95%CI were estimated by CMH method considering stratification factors.

**Table S11:** **Comparison of the neutralizing antibody response against Omicron (BA.1.1) variant elicited by BBIBP-CorV booster between male and female subjects (PPS)**

|  | **BBIBP-CorV** | | |
| --- | --- | --- | --- |
|  | **Male** | **Female** | ***p* value** |
| **All subjects** |  |  |  |
| N(missing) | 237(0) | 12(0) |  |
| Pre-booster antibody titer ≥4, n (%) | 232(97.89) | 10(83.33) | 0.0392 |
| 95%CI (%) | 95.15-99.31 | 51.59-97.91 |  |
| Pre-booster antibody GMT^[1]^ (95%CI) | 37.85(32.60-43.94) | 39.19(12.47-123.19) | 0.9221 |
| Post-booster antibody GMT (95%CI) | 142.16(123.24-163.98) | 166.09(71.83-384.04) | 0.6409 |
| Ratio of GMT between two groups(95%CI)^[2]^ | 0.86(0.44-1.65) |  |  |
| Post-booster adjusted antibody GMT (95%CI)^[3]^ | 100.42(77.87-129.50) | 111.27(67.50-183.41) | 0.6474 |
| Ratio of adjusted GMT between two groups(95%CI)^[3]^ | 0.90(0.58-1.40) |  |  |
| Rate of 4-fold rise^[4]^, n (%) | 127(53.59) | 7(58.33) | 0.8351 |
| 95%CI (%) | 47.02-60.07 | 27.67-84.83 |  |
| Rate difference between two groups (%, 95%CI^[5]^) | -3.07(-31.70-25.56) |  |  |
| Post-booster antibody GMT fold rise (95%CI) | 3.76(3.37-4.19) | 4.24(2.27-7.92) | 0.6332 |
| **All subjects (Excluding participants with positive**  **or weak positive COVID-19 PCR test)** |  |  |  |
| N(missing) | 216(0) | 10(0) |  |
| Pre-booster antibody titer ≥4, n (%) | 211(97.69) | 9(90.00) | 0.2402 |
| 95%CI (%) | 94.68-99.24 | 55.50-99.75 |  |
| Pre-booster antibody GMT^[1]^ (95%CI) | 37.89(32.35-44.38) | 51.71(15.30-174.76) | 0.4253 |
| Post-booster antibody GMT (95%CI) | 147.58(127.19-171.26) | 206.86(79.19-540.32) | 0.3521 |
| Ratio of GMT between two groups(95%CI)^[2]^ | 0.71(0.35-1.46) |  |  |
| Post-booster adjusted antibody GMT (95%CI)^[3]^ | 99.70(74.89-132.74) | 109.56(63.12-190.18) | 0.6995 |
| Ratio of adjusted GMT between two groups(95%CI)^[3]^ | 0.91(0.56-1.47) |  |  |
| Rate of 4-fold rise^[4]^, n (%) | 118(54.63) | 6(60.00) | 0.8257 |
| 95%CI (%) | 47.73-61.40 | 26.24-87.84 |  |
| Rate difference between two groups (%, 95%CI^[5]^) | -3.54(-34.64-27.56) |  |  |
| Post-booster antibody GMT fold rise (95%CI) | 3.90(3.47-4.37) | 4.00(2.20-7.26) | 0.9237 |

Notes: [1] GMT represent geometric mean titer.

[2] The ratio of GMT between two groups was calculated by “NVSI-06-09/ BBIBP-CorV”.

[3] Covariance analysis with least square method was used to calculate the adjusted GMT and the corresponding *p* value.

[4] Rate of 4-fold rise was defined as percentage of participants with a ≥4-fold rise in neutralizing antibody titer from baseline.

[5] Rate difference=(NVSI-06-09)-(BBIBP-CorV). Rate difference and 95%CI were estimated by CMH method considering stratification factors.

**Table S12:** **Comparison of the neutralizing antibody response against prototype SARS-CoV-2 strain elicited by NVSI-06-09 booster between different age subgroups (PPS)**

|  | **NVSI-06-09** | | |
| --- | --- | --- | --- |
|  | **<45 year** | **≥45 year** | ***p* value** |
| **All subjects** |  |  |  |
| N(missing) | 199(0) | 56(0) |  |
| Pre-booster antibody titer ≥4, n (%) | 198(99.50) | 56(100.00) | 1.0000 |
| 95%CI (%) | 97.23-99.99 | 93.62-100.00 |  |
| Pre-booster antibody GMT^[1]^ (95%CI) | 122.22(107.64-138.79) | 109.98(83.92-144.13) | 0.4545 |
| Post-booster antibody GMT (95%CI) | 2233.49(1927.52-2588.03) | 2812.37(2109.65-3749.17) | 0.1512 |
| Ratio of GMT between two groups(95%CI)^[2]^ | 0.79(0.58-1.09) |  |  |
| Post-booster adjusted antibody GMT (95%CI)^[3]^ | 1725.98(1250.30-2382.62) | 2205.25(1474.36-3298.49) | 0.1211 |
| Ratio of adjusted GMT between two groups(95%CI)^[3]^ | 0.78(0.57-1.07) |  |  |
| Rate of 4-fold rise^[4]^, n (%) | 185(92.96) | 53(94.64) | 0.6621 |
| 95%CI (%) | 88.48-96.10 | 85.13-98.88 |  |
| Rate difference between two groups (%, 95%CI^[5]^) | -1.65(-8.54-5.23) |  |  |
| Post-booster antibody GMT fold rise (95%CI) | 18.27(15.43-21.65) | 25.57(17.08-38.29) | 0.0843 |
| **All subjects (Excluding participants with positive**  **or weak positive COVID-19 PCR test)** |  |  |  |
| N(missing) | 180(0) | 51(0) |  |
| Pre-booster antibody titer ≥4, n (%) | 179(99.44) | 51(100.00) | 1.0000 |
| 95%CI (%) | 96.94-99.99 | 93.02-100.00 |  |
| Pre-booster antibody GMT^[1]^ (95%CI) | 122.21(106.83-139.79) | 113.88(85.78-151.18) | 0.6347 |
| Post-booster antibody GMT (95%CI) | 2189.77(1872.90-2560.26) | 2855.43(2104.69-3873.96) | 0.1184 |
| Ratio of GMT between two groups(95%CI)^[2]^ | 0.77(0.55-1.07) |  |  |
| Post-booster adjusted antibody GMT (95%CI)^[3]^ | 1707.97(1231.33-2369.10) | 2243.20(1481.96-3395.47) | 0.1033 |
| Ratio of adjusted GMT between two groups(95%CI)^[3]^ | 0.76(0.55-1.06) |  |  |
| Rate of 4-fold rise^[4]^, n (%) | 166(92.22) | 48(94.12) | 0.6518 |
| 95%CI (%) | 87.29-95.68 | 83.76-98.77 |  |
| Rate difference between two groups (%, 95%CI^[5]^) | -1.88(-9.43-5.67) |  |  |
| Post-booster antibody GMT fold rise (95%CI) | 17.92(14.98-21.43) | 25.07(16.30-38.58) | 0.1027 |

Notes: [1] GMT represent geometric mean titer.

[2] The ratio of GMT between two groups was calculated by “NVSI-06-09/BBIBP-CorV”.

[3] Covariance analysis with least square method was used to calculate the adjusted GMT and the corresponding *p* value.

[4] Rate of 4-fold rise was defined as percentage of participants with a ≥4-fold rise in neutralizing antibody titer from baseline.

[5] Rate difference=(NVSI-06-09)-(BBIBP-CorV). Rate difference and 95%CI were estimated by CMH method considering stratification factors.

**Table S13: Comparison of the neutralizing antibody response against prototype SARS-CoV-2 strain elicited by BBIBP-CorV booster between different age subgroups (PPS)**

|  | **BBIBP-CorV** | | |
| --- | --- | --- | --- |
|  | **<45 year** | **≥45 year** | ***p* value** |
| **All subjects** |  |  |  |
| N(missing) | 198(0) | 51(0) |  |
| Pre-booster antibody titer ≥4, n (%) | 197(99.49) | 50(98.04) | 0.3683 |
| 95%CI (%) | 97.22-99.99 | 89.55-99.95 |  |
| Pre-booster antibody GMT^[1]^ (95%CI) | 112.68(99.06-128.17) | 115.70(84.61-158.22) | 0.8608 |
| Post-booster antibody GMT (95%CI) | 487.49(438.33-542.16) | 587.45(450.32-766.33) | 0.1387 |
| Ratio of GMT between two groups(95%CI)^[2]^ | 0.83(0.65-1.06) |  |  |
| Post-booster adjusted antibody GMT (95%CI)^[3]^ | 437.61(344.99-555.10) | 524.85(397.09-693.71) | 0.0943 |
| Ratio of adjusted GMT between two groups(95%CI)^[3]^ | 0.83(0.67-1.03) |  |  |
| Rate of 4-fold rise^[4]^, n (%) | 119(60.10) | 29(56.86) | 0.7175 |
| 95%CI (%) | 52.92-66.98 | 42.25-70.65 |  |
| Rate difference between two groups (%, 95%CI^[5]^) | 2.81(-12.39-18.00) |  |  |
| Post-booster antibody GMT fold rise (95%CI) | 4.33(3.86-4.85) | 5.08(3.74-6.89) | 0.2482 |
| **All subjects (Excluding participants with positive**  **or weak positive COVID-19 PCR test)** |  |  |  |
| N(missing) | 180(0) | 46(0) |  |
| Pre-booster antibody titer ≥4, n (%) | 179(99.44) | 46(100.00) | 1.0000 |
| 95%CI (%) | 96.94-99.99 | 92.29-100.00 |  |
| Pre-booster antibody GMT^[1]^ (95%CI) | 113.51(99.23-129.85) | 125.73(93.92-168.33) | 0.5057 |
| Post-booster antibody GMT (95%CI) | 496.40(443.06-556.17) | 591.06(446.55-782.34) | 0.1936 |
| Ratio of GMT between two groups(95%CI)^[2]^ | 0.84(0.65-1.09) |  |  |
| Post-booster adjusted antibody GMT (95%CI)^[3]^ | 400.49(305.47-525.07) | 459.07(334.39-630.24) | 0.2393 |
| Ratio of adjusted GMT between two groups(95%CI)^[3]^ | 0.87(0.69-1.10) |  |  |
| Rate of 4-fold rise^[4]^, n (%) | 109(60.56) | 25(54.35) | 0.4806 |
| 95%CI (%) | 53.01-67.75 | 39.01-69.10 |  |
| Rate difference between two groups (%, 95%CI^[5]^) | 5.72(-10.21-21.65) |  |  |
| Post-booster antibody GMT fold rise (95%CI) | 4.37(3.87-4.94) | 4.70(3.47-6.36) | 0.6173 |

Notes: [1] GMT represent geometric mean titer.

[2] The ratio of GMT between two groups was calculated by “NVSI-06-09/BBIBP-CorV”.

[3] Covariance analysis with least square method was used to calculate the adjusted GMT and the corresponding *p* value.

[4] Rate of 4-fold rise was defined as percentage of participants with a ≥4-fold rise in neutralizing antibody titer from baseline.

[5] Rate difference=(NVSI-06-09)-(BBIBP-CorV). Rate difference and 95%CI were estimated by CMH method considering stratification factors.

**Table S14: Comparison of the neutralizing antibody response against prototype SARS-CoV-2 strain elicited by NVSI-06-09 booster between different age subgroups (PPS)**

|  | **NVSI-06-09** | | |
| --- | --- | --- | --- |
|  | **45-59 year** | **≥60 year** | ***p* value** |
| **All subjects** |  |  |  |
| N(missing) | 52(0) | 4(0) |  |
| Pre-booster antibody titer ≥4, n (%) | 52(100.00) | 4(100.00) | 1.0000 |
| 95%CI (%) | 93.15-100.00 | 39.76-100.00 |  |
| Pre-booster antibody GMT^[1]^ (95%CI) | 109.66(82.81-145.21) | 114.16(17.36-750.91) | 0.9396 |
| Post-booster antibody GMT (95%CI) | 2784.22(2062.96-3757.66) | 3205.29(491.45-20905.30) | 0.8030 |
| Ratio of GMT between two groups(95%CI)^[2]^ | 0.87(0.28-2.68) |  |  |
| Post-booster adjusted antibody GMT (95%CI)^[3]^ | 3403.22(1527.21-7583.69) | 3990.26(1021.18-15591.90) | 0.7816 |
| Ratio of adjusted GMT between two groups(95%CI)^[3]^ | 0.85(0.27-2.68) |  |  |
| Rate of 4-fold rise^[4]^, n (%) | 49(94.23) | 4(100.0) | 0.6175 |
| 95%CI (%) | 84.05-98.79 | 39.76-100.00 |  |
| Rate difference between two groups (%, 95%CI^[5]^) | -6.00(-12.58-0.58) |  |  |
| Post-booster antibody GMT fold rise (95%CI) | 25.39(16.57-38.91) | 28.08(3.61-218.32) | 0.8991 |
| **All subjects (Excluding participants with positive**  **or weak positive COVID-19 PCR test)** |  |  |  |
| N(missing) | 47(0) | 4(0) |  |
| Pre-booster antibody titer ≥4, n (%) | 47(100.00) | 4(100.00) | 1.0000 |
| 95%CI (%) | 92.45-100.00 | 39.76-100.00 |  |
| Pre-booster antibody GMT^[1]^ (95%CI) | 113.85(84.74-152.97) | 114.16(17.36-750.91) | 0.9959 |
| Post-booster antibody GMT (95%CI) | 2827.48(2053.44-3893.29) | 3205.29(491.45-20905.30) | 0.8269 |
| Ratio of GMT between two groups(95%CI)^[2]^ | 0.88(0.28-2.78) |  |  |
| Post-booster adjusted antibody GMT (95%CI)^[3]^ | 3393.93(1500.84-7674.88) | 3914.05(979.16-15645.83) | 0.8070 |
| Ratio of adjusted GMT between two groups(95%CI)^[3]^ | 0.87(0.27-2.79) |  |  |
| Rate of 4-fold rise^[4]^, n (%) | 44(93.62) | 4(100.0) | 0.5978 |
| 95%CI (%) | 82.46-98.66 | 39.76-100.00 |  |
| Rate difference between two groups (%, 95%CI^[5]^) | -6.67(-13.95-0.62) |  |  |
| Post-booster antibody GMT fold rise (95%CI) | 24.83(15.70-39.29) | 28.08(3.61-218.32) | 0.8796 |

Notes: [1] GMT represent geometric mean titer.

[2] The ratio of GMT between two groups was calculated by “NVSI-06-09/BBIBP-CorV”.

[3] Covariance analysis with least square method was used to calculate the adjusted GMT and the corresponding *p* value.

[4] Rate of 4-fold rise was defined as percentage of participants with a ≥4-fold rise in neutralizing antibody titer from baseline.

[5] Rate difference=(NVSI-06-09)-(BBIBP-CorV). Rate difference and 95%CI were estimated by CMH method considering stratification factors.

**Table S15: Comparison of the neutralizing antibody response against prototype SARS-CoV-2 strain elicited by BBIBP-CorV booster between different age subgroups (PPS)**

|  | **BBIBP-CorV** | | |
| --- | --- | --- | --- |
|  | **45-59 year** | **≥60 year** | ***p* value** |
| **All subjects** |  |  |  |
| N(missing) | 50(0) | 1(0) |  |
| Pre-booster antibody titer ≥4, n (%) | 49(98.00) | 1(100.00) | 1.0000 |
| 95%CI (%) | 89.35-99.95 | 2.50-100.00 |  |
| Pre-booster antibody GMT^[1]^ (95%CI) | 112.96(82.38-154.89) | 384.00(NA) | 0.2807 |
| Post-booster antibody GMT (95%CI) | 568.33(437.02-739.09) | 3072.00(NA) | 0.0769 |
| Ratio of GMT between two groups(95%CI)^[2]^ | 0.19(0.03-1.21) |  |  |
| Post-booster adjusted antibody GMT (95%CI)^[3]^ | 559.75(334.19-937.55) | 1929.22(312.22-11920.76) | 0.1616 |
| Ratio of adjusted GMT between two groups(95%CI)^[3]^ | 0.29(0.05-1.67) |  |  |
| Rate of 4-fold rise^[4]^, n (%) | 28(56.00) | 1(100.0) | 0.3980 |
| 95%CI (%) | 41.25-70.01 | 2.50-100.00 |  |
| Rate difference between two groups (%, 95%CI^[5]^) | -42.55(-56.69--28.42) |  |  |
| Post-booster antibody GMT fold rise (95%CI) | 5.03(3.69-6.86) | 8.00(NA) | 0.6760 |
| **All subjects (Excluding participants with positive**  **or weak positive COVID-19 PCR test)** |  |  |  |
| N(missing) | 45(0) | 1(0) |  |
| Pre-booster antibody titer ≥4, n (%) | 45(100.00) | 1(100.00) | 1.0000 |
| 95%CI (%) | 92.13-100.00 | 2.50-100.00 |  |
| Pre-booster antibody GMT^[1]^ (95%CI) | 122.65(91.40-164.59) | 384.00(NA) | 0.2551 |
| Post-booster antibody GMT (95%CI) | 569.80(432.05-751.47) | 3072.00(NA) | 0.0773 |
| Ratio of GMT between two groups(95%CI)^[2]^ | 0.19(0.03-1.21) |  |  |
| Post-booster adjusted antibody GMT (95%CI)^[3]^ | 517.58(275.11-973.75) | 1748.60(266.06-11492.04) | 0.1739 |
| Ratio of adjusted GMT between two groups(95%CI)^[3]^ | 0.30(0.05-1.75) |  |  |
| Rate of 4-fold rise^[4]^, n (%) | 24(53.33) | 1(100.0) | 0.3833 |
| 95%CI (%) | 37.87-68.34 | 2.50-100.00 |  |
| Rate difference between two groups (%, 95%CI^[5]^) | -44.19(-59.03--29.34) |  |  |
| Post-booster antibody GMT fold rise (95%CI) | 4.65(3.41-6.33) | 8.00(NA) | 0.6035 |

Notes: [1] GMT represent geometric mean titer.

[2] The ratio of GMT between two groups was calculated by “NVSI-06-09/BBIBP-CorV”.

[3] Covariance analysis with least square method was used to calculate the adjusted GMT and the corresponding *p* value.

[4] Rate of 4-fold rise was defined as percentage of participants with a ≥4-fold rise in neutralizing antibody titer from baseline.

[5] Rate difference=(NVSI-06-09)-(BBIBP-CorV). Rate difference and 95%CI were estimated by CMH method considering stratification factors.

**Table S16:** **Comparison of the neutralizing antibody response against Omicron (BA.1.1) variant elicited by NVSI-06-09 booster between different age subgroups (PPS)**

|  | **NVSI-06-09** | | |
| --- | --- | --- | --- |
|  | **<45 year** | **≥45 year** | ***p* value** |
| **All subjects** |  |  |  |
| N(missing) | 199(0) | 56(0) |  |
| Pre-booster antibody titer ≥4, n (%) | 193(96.98) | 54(96.43) | 0.6890 |
| 95%CI (%) | 93.55-98.89 | 87.69-99.56 |  |
| Pre-booster antibody GMT^[1]^ (95%CI) | 41.36(35.03-48.82) | 37.46(26.47-52.99) | 0.5893 |
| Post-booster antibody GMT (95%CI) | 1669.89(1375.13-2027.83) | 1878.93(1322.21-2670.06) | 0.5706 |
| Ratio of GMT between two groups(95%CI)^[2]^ | 0.89(0.59-1.34) |  |  |
| Post-booster adjusted antibody GMT (95%CI)^[3]^ | 1362.19(910.24-2038.55) | 1582.34(957.30-2615.46) | 0.4482 |
| Ratio of adjusted GMT between two groups(95%CI)^[3]^ | 0.86(0.58-1.27) |  |  |
| Rate of 4-fold rise^[4]^, n (%) | 192(96.48) | 55(98.21) | 0.5254 |
| 95%CI (%) | 92.89-98.57 | 90.45-99.95 |  |
| Rate difference between two groups (%, 95%CI^[5]^) | -1.67(-5.98-2.63) |  |  |
| Post-booster antibody GMT fold rise (95%CI) | 40.38(32.90-49.55) | 50.16(32.05-78.52) | 0.3438 |
| **All subjects (Excluding participants with positive**  **or weak positive COVID-19 PCR test)** |  |  |  |
| N(missing) | 180(0) | 51(0) |  |
| Pre-booster antibody titer ≥4, n (%) | 174(96.67) | 50(98.04) | 1.0000 |
| 95%CI (%) | 92.89-98.77 | 89.55-99.95 |  |
| Pre-booster antibody GMT^[1]^ (95%CI) | 42.13(35.41-50.13) | 40.07(28.19-56.96) | 0.7917 |
| Post-booster antibody GMT (95%CI) | 1651.60(1342.41-2032.01) | 2049.09(1441.15-2913.48) | 0.3243 |
| Ratio of GMT between two groups(95%CI)^[2]^ | 0.81(0.52-1.24) |  |  |
| Post-booster adjusted antibody GMT (95%CI)^[3]^ | 1356.26(901.28-2040.92) | 1704.37(1016.01-2859.12) | 0.2746 |
| Ratio of adjusted GMT between two groups(95%CI)^[3]^ | 0.80(0.53-1.20) |  |  |
| Rate of 4-fold rise^[4]^, n (%) | 173(96.11) | 50(98.04) | 0.5202 |
| 95%CI (%) | 92.15-98.42 | 89.55-99.95 |  |
| Rate difference between two groups (%, 95%CI^[5]^) | -1.87(-6.59-2.86) |  |  |
| Post-booster antibody GMT fold rise (95%CI) | 39.20(31.53-48.74) | 51.14(31.89-82.00) | 0.2735 |

Notes: [1] GMT represent geometric mean titer.

[2] The ratio of GMT between two groups was calculated by “NVSI-06-09/BBIBP-CorV”.

[3] Covariance analysis with least square method was used to calculate the adjusted GMT and the corresponding *p* value.

[4] Rate of 4-fold rise was defined as percentage of participants with a ≥4-fold rise in neutralizing antibody titer from baseline.

[5] Rate difference=(NVSI-06-09)-(BBIBP-CorV). Rate difference and 95%CI were estimated by CMH method considering stratification factors.

**Table S17: Comparison of the neutralizing antibody response against Omicron (BA.1.1) variant elicited by BBIBP-CorV booster between different age subgroups (PPS)**

|  | **BBIBP-CorV** | | |
| --- | --- | --- | --- |
|  | **<45 year** | **≥45 year** | ***p* value** |
| **All subjects** |  |  |  |
| N(missing) | 198(0) | 51(0) |  |
| Pre-booster antibody titer ≥4, n (%) | 194(97.98) | 48(94.12) | 0.1536 |
| 95%CI (%) | 94.91-99.45 | 83.76-98.77 |  |
| Pre-booster antibody GMT^[1]^ (95%CI) | 37.04(31.41-43.68) | 41.50(28.83-59.75) | 0.5469 |
| Post-booster antibody GMT (95%CI) | 138.65(118.33-162.46) | 162.48(119.28-221.32) | 0.3700 |
| Ratio of GMT between two groups(95%CI)^[2]^ | 0.85(0.60-1.21) |  |  |
| Post-booster adjusted antibody GMT (95%CI)^[3]^ | 98.03(75.49-127.29) | 108.47(79.80-147.43) | 0.3955 |
| Ratio of adjusted GMT between two groups(95%CI)^[3]^ | 0.90(0.72-1.14) |  |  |
| Rate of 4-fold rise^[4]^, n (%) | 110(55.56) | 24(47.06) | 0.3475 |
| 95%CI (%) | 48.34-62.60 | 32.93-61.54 |  |
| Rate difference between two groups (%, 95%CI^[5]^) | 7.26(-8.01-22.54) |  |  |
| Post-booster antibody GMT fold rise (95%CI) | 3.74(3.33-4.21) | 3.91(3.03-5.06) | 0.7388 |
| **All subjects (Excluding participants with positive**  **or weak positive COVID-19 PCR test)** |  |  |  |
| N(missing) | 180(0) | 46(0) |  |
| Pre-booster antibody titer ≥4, n (%) | 176(97.78) | 44(95.65) | 0.6043 |
| 95%CI (%) | 94.41-99.39 | 85.16-99.47 |  |
| Pre-booster antibody GMT^[1]^ (95%CI) | 37.64(31.59-44.85) | 41.58(28.60-60.45) | 0.6179 |
| Post-booster antibody GMT (95%CI) | 146.42(124.18-172.63) | 163.83(117.33-228.75) | 0.5445 |
| Ratio of GMT between two groups(95%CI)^[2]^ | 0.89(0.62-1.29) |  |  |
| Post-booster adjusted antibody GMT (95%CI)^[3]^ | 98.53(73.58-131.94) | 104.31(74.14-146.76) | 0.6470 |
| Ratio of adjusted GMT between two groups(95%CI)^[3]^ | 0.94(0.74-1.21) |  |  |
| Rate of 4-fold rise^[4]^, n (%) | 102(56.67) | 22(47.83) | 0.3226 |
| 95%CI (%) | 49.09-64.02 | 32.89-63.05 |  |
| Rate difference between two groups (%, 95%CI^[5]^) | 7.96(-7.86-23.78) |  |  |
| Post-booster antibody GMT fold rise (95%CI) | 3.89(3.44-4.40) | 3.94(3.01-5.15) | 0.9273 |

Notes: [1] GMT represent geometric mean titer.

[2] The ratio of GMT between two groups was calculated by “NVSI-06-09/BBIBP-CorV”.

[3] Covariance analysis with least square method was used to calculate the adjusted GMT and the corresponding *p* value.

[4] Rate of 4-fold rise was defined as percentage of participants with a ≥4-fold rise in neutralizing antibody titer from baseline.

[5] Rate difference=(NVSI-06-09)-(BBIBP-CorV). Rate difference and 95%CI were estimated by CMH method considering stratification factors.

**Table S18: Comparison of the neutralizing antibody response against Omicron (BA.1.1) variant elicited by NVSI-06-09 booster between different age subgroups (PPS)**

|  | **NVSI-06-09** | | |
| --- | --- | --- | --- |
|  | **45-59 year** | **≥60 year** | ***p* value** |
| **All subjects** |  |  |  |
| N(missing) | 52(0) | 4(0) |  |
| Pre-booster antibody titer ≥4, n (%) | 50(96.15) | 4(100.00) | 1.0000 |
| 95%CI (%) | 86.79-99.53 | 39.76-100.00 |  |
| Pre-booster antibody GMT^[1]^ (95%CI) | 38.04(26.41-54.79) | 30.67(4.31-218.36) | 0.7520 |
| Post-booster antibody GMT (95%CI) | 1852.02(1300.45-2637.54) | 2266.48(90.51-56752.90) | 0.7698 |
| Ratio of GMT between two groups(95%CI)^[2]^ | 0.82(0.21-3.24) |  |  |
| Post-booster adjusted antibody GMT (95%CI)^[3]^ | 3199.38(1221.93-8376.93) | 4294.27(835.47-22072.23) | 0.6671 |
| Ratio of adjusted GMT between two groups(95%CI)^[3]^ | 0.75(0.19-2.92) |  |  |
| Rate of 4-fold rise^[4]^, n (%) | 51(98.08) | 4(100.0) | 0.7773 |
| 95%CI (%) | 89.74-99.95 | 39.76-100.00 |  |
| Rate difference between two groups (%, 95%CI^[5]^) | -2.00(-5.88-1.88) |  |  |
| Post-booster antibody GMT fold rise (95%CI) | 48.69(30.45-77.86) | 73.90(5.21-1047.9) | 0.6352 |
| **All subjects (Excluding participants with positive**  **or weak positive COVID-19 PCR test)** |  |  |  |
| N(missing) | 47(0) | 4(0) |  |
| Pre-booster antibody titer ≥4, n (%) | 46(97.87) | 4(100.00) | 1.0000 |
| 95%CI (%) | 88.71-99.95 | 39.76-100.00 |  |
| Pre-booster antibody GMT^[1]^ (95%CI) | 40.99(28.30-59.39) | 30.67(4.31-218.36) | 0.6605 |
| Post-booster antibody GMT (95%CI) | 2031.58(1429.34-2887.56) | 2266.48(90.51-56752.90) | 0.8687 |
| Ratio of GMT between two groups(95%CI)^[2]^ | 0.90(0.24-3.36) |  |  |
| Post-booster adjusted antibody GMT (95%CI)^[3]^ | 3200.02(1246.38-8215.86) | 3877.36(780.39-19264.60) | 0.7737 |
| Ratio of adjusted GMT between two groups(95%CI)^[3]^ | 0.83(0.22-3.14) |  |  |
| Rate of 4-fold rise^[4]^, n (%) | 46(97.87) | 4(100.0) | 0.7656 |
| 95%CI (%) | 88.71-99.95 | 39.76-100.00 |  |
| Rate difference between two groups (%, 95%CI^[5]^) | -2.22(-6.53-2.08) |  |  |
| Post-booster antibody GMT fold rise (95%CI) | 49.56(30.14-81.50) | 73.90(5.21-1047.9) | 0.6524 |

Notes: [1] GMT represent geometric mean titer.

[2] The ratio of GMT between two groups was calculated by “NVSI-06-09/BBIBP-CorV”.

[3] Covariance analysis with least square method was used to calculate the adjusted GMT and the corresponding *p* value.

[4] Rate of 4-fold rise was defined as percentage of participants with a ≥4-fold rise in neutralizing antibody titer from baseline.

[5] Rate difference=(NVSI-06-09)-(BBIBP-CorV). Rate difference and 95%CI were estimated by CMH method considering stratification factors.

**Table S19: Comparison of the neutralizing antibody response against Omicron (BA.1.1) variant elicited by BBIBP-CorV booster between different age subgroups (PPS)**

|  | **BBIBP-CorV** | | |
| --- | --- | --- | --- |
|  | **45-59 year** | **≥60 year** | ***p* value** |
| **All subjects** |  |  |  |
| N(missing) | 50(0) | 1(0) |  |
| Pre-booster antibody titer ≥4, n (%) | 47(94.00) | 1(100.00) | 1.0000 |
| 95%CI (%) | 83.45-98.75 | 2.50-100.00 |  |
| Pre-booster antibody GMT^[1]^ (95%CI) | 40.58(28.06-58.69) | 128.00(NA) | 0.3853 |
| Post-booster antibody GMT (95%CI) | 152.32(114.39-202.83) | 4096.00(NA) | 0.0022 |
| Ratio of GMT between two groups(95%CI)^[2]^ | 0.04(0.00-0.29) |  |  |
| Post-booster adjusted antibody GMT (95%CI)^[3]^ | 138.55(91.54-209.70) | 1880.49(436.35-8104.15) | 0.0005 |
| Ratio of adjusted GMT between two groups(95%CI)^[3]^ | 0.07(0.02-0.30) |  |  |
| Rate of 4-fold rise^[4]^, n (%) | 23(46.00) | 1(100.0) | 0.2971 |
| 95%CI (%) | 31.81-60.68 | 2.50-100.00 |  |
| Rate difference between two groups (%, 95%CI^[5]^) | -53.19(-67.46--38.93) |  |  |
| Post-booster antibody GMT fold rise (95%CI) | 3.75(2.93-4.81) | 32.00(NA) | 0.0186 |
| **All subjects (Excluding participants with positive**  **or weak positive COVID-19 PCR test)** |  |  |  |
| N(missing) | 45(0) | 1(0) |  |
| Pre-booster antibody titer ≥4, n (%) | 43(95.56) | 1(100.00) | 1.0000 |
| 95%CI (%) | 84.85-99.46 | 2.50-100.00 |  |
| Pre-booster antibody GMT^[1]^ (95%CI) | 40.55(27.75-59.26) | 128.00(NA) | 0.3728 |
| Post-booster antibody GMT (95%CI) | 152.52(112.08-207.55) | 4096.00(NA) | 0.0027 |
| Ratio of GMT between two groups(95%CI)^[2]^ | 0.04(0.00-0.30) |  |  |
| Post-booster adjusted antibody GMT (95%CI)^[3]^ | 121.97(72.97-203.88) | 1581.28(345.19-7243.70) | 0.0009 |
| Ratio of adjusted GMT between two groups(95%CI)^[3]^ | 0.08(0.02-0.33) |  |  |
| Rate of 4-fold rise^[4]^, n (%) | 21(46.67) | 1(100.0) | 0.3173 |
| 95%CI (%) | 31.66-62.13 | 2.50-100.00 |  |
| Rate difference between two groups (%, 95%CI^[5]^) | -51.16(-66.10--36.22) |  |  |
| Post-booster antibody GMT fold rise (95%CI) | 3.76(2.91-4.86) | 32.00(NA) | 0.0172 |

Notes: [1] GMT represent geometric mean titer.

[2] The ratio of GMT between two groups was calculated by “NVSI-06-09/BBIBP-CorV”.

[3] Covariance analysis with least square method was used to calculate the adjusted GMT and the corresponding *p* value.

[4] Rate of 4-fold rise was defined as percentage of participants with a ≥4-fold rise in neutralizing antibody titer from baseline.

[5] Rate difference=(NVSI-06-09)-(BBIBP-CorV). Rate difference and 95%CI were estimated by CMH method considering stratification factors.

**Table S20: Live-virus neutralizing antibody response against Omicron (BA.2) variant (PPS)**

|  | **14 days after boosting** | | |
| --- | --- | --- | --- |
|  | **NVSI-06-09** | **BBIBP-CorV** | ***p* value** |
| **All subjects** |  |  |  |
| N(missing) | 255(1)^[6]^ | 249(0) |  |
| Pre-booster antibody titer ≥4, n (%) | 246(96.85) | 241(96.79) | 0.9678 |
| 95%CI (%) | 93.89-98.63 | 93.77-98.60 |  |
| Pre-booster antibody GMT^[1]^ (95%CI) | 39.19(34.27-44.82) | 40.35(35.05-46.45) | 0.7689 |
| Post-booster antibody GMT (95%CI) | 983.36(852.25-1134.64) | 117.33(103.07-133.55) | <0.0001 |
| Ratio of GMT between two groups(95%CI)^[2]^ | 8.38(6.91-10.16) |  |  |
| Post-booster adjusted antibody GMT (95%CI)^[3]^ | 939.60(742.60-1188.86) | 110.57(87.09-140.38) | <0.0001 |
| Ratio of adjusted GMT between two groups(95%CI)^[3]^ | 8.50(7.15-10.10) |  |  |
| Rate of 4-fold rise^[4]^, n (%) | 240(94.49) | 104(41.77) | <0.0001 |
| 95%CI (%) | 90.92-96.95 | 35.57-48.16 |  |
| Rate difference between two groups (%, 95%CI^[5]^) | 52.76(46.02-59.51) |  |  |
| Post-booster antibody GMT fold rise (95%CI) | 25.09(21.17-29.73) | 2.91(2.60-3.25) | <0.0001 |
| **All subjects (Excluding participants with positive**  **or weak positive COVID-19 PCR test)** |  |  |  |
| N(missing) | 231(1) ^[6]^ | 226(0) |  |
| Pre-booster antibody titer ≥4, n (%) | 224(97.39) | 218(96.46) | 0.5644 |
| 95%CI (%) | 94.41-99.04 | 93.14-98.46 |  |
| Pre-booster antibody GMT^[1]^ (95%CI) | 40.35(35.15-46.33) | 40.53(34.98-46.97) | 0.9649 |
| Post-booster antibody GMT (95%CI) | 985.80(847.56-1146.58) | 118.60(103.61-135.75) | <0.0001 |
| Ratio of GMT between two groups(95%CI)^[2]^ | 8.31(6.79-10.18) |  |  |
| Post-booster adjusted antibody GMT (95%CI)^[3]^ | 914.93(713.58-1173.11) | 109.55(84.81-141.50) | <0.0001 |
| Ratio of adjusted GMT between two groups(95%CI)^[3]^ | 8.35(6.95-10.03) |  |  |
| Rate of 4-fold rise^[4]^, n (%) | 217(94.35) | 98(43.36) | <0.0001 |
| 95%CI (%) | 90.53-96.96 | 36.81-50.10 |  |
| Rate difference between two groups (%, 95%CI^[5]^) | 51.03(43.89-58.16) |  |  |
| Post-booster antibody GMT fold rise (95%CI) | 24.43(20.44-29.20) | 2.93(2.59-3.30) | <0.0001 |
| **Subjects who have received 2 doses of inactivated vaccine previously** |  |  |  |
| N(missing) | 11(0) | 9(0) |  |
| Pre-booster antibody titer ≥4, n (%) | 11(100.00) | 9(100.00) | 1.0000 |
| 95%CI (%) | 71.51-100.00 | 66.37-100.00 |  |
| Pre-booster antibody GMT^[1]^ (95%CI) | 28.69(16.55-49.72) | 80.17(53.95-119.13) | 0.0043 |
| Post-booster antibody GMT (95%CI) | 724.52(416.59-1260.06) | 153.28(79.97-293.80) | 0.0006 |
| Ratio of GMT between two groups(95%CI)^[2]^ | 4.73(2.15-10.39) |  |  |
| Post-booster adjusted antibody GMT (95%CI)^[3]^ | 747.89(405.03-1380.97) | 147.44(73.68-295.04) | 0.0039 |
| Ratio of adjusted GMT between two groups(95%CI)^[3]^ | 5.07(1.82-14.14) |  |  |
| Rate of 4-fold rise^[4]^, n (%) | 11(100.0) | 2(22.22) | 0.0004 |
| 95%CI (%) | 71.51-100.00 | 2.81-60.01 |  |
| Rate difference between two groups (%, 95%CI^[5]^) | 77.78(50.62-100.00) |  |  |
| Post-booster antibody GMT fold rise (95%CI) | 25.26(11.26-56.65) | 1.91(1.00-3.65) | <0.0001 |
| **Subjects who have received 3 doses of inactivated vaccine previously** |  |  |  |
| N(missing) | 244(1) ^[6]^ | 240(0) |  |
| Pre-booster antibody titer ≥4, n (%) | 235(96.71) | 232(96.67) | 0.9798 |
| 95%CI (%) | 93.62-98.57 | 93.54-98.55 |  |
| Pre-booster antibody GMT^[1]^ (95%CI) | 39.75(34.61-45.66) | 39.32(34.03-45.44) | 0.9149 |
| Post-booster antibody GMT (95%CI) | 997.05(859.99-1155.95) | 116.16(101.71-132.66) | <0.0001 |
| Ratio of GMT between two groups(95%CI)^[2]^ | 8.58(7.04-10.47) |  |  |
| Post-booster adjusted antibody GMT (95%CI)^[3]^ | 994.62(878.04-1126.68) | 116.44(102.72-132.01) | <0.0001 |
| Ratio of adjusted GMT between two groups(95%CI)^[3]^ | 8.54(7.16-10.19) |  |  |
| Rate of 4-fold rise^[4]^, n (%) | 229(94.24) | 102(42.50) | <0.0001 |
| 95%CI (%) | 90.52-96.81 | 36.16-49.02 |  |
| Rate difference between two groups (%, 95%CI^[5]^) | 51.74(44.83-58.65) |  |  |
| Post-booster antibody GMT fold rise (95%CI) | 25.08(21.06-29.88) | 2.95(2.64-3.30) | <0.0001 |

Notes: [1] GMT represent geometric mean titer.

[2] The ratio of GMT between two groups was calculated by “NVSI-06-09/ BBIBP-CorV”.

[3] Covariance analysis with least square method was used to calculate the adjusted GMT and the corresponding *p* value.

[4] Rate of 4-fold rise was defined as percentage of participants with a ≥4-fold rise in neutralizing antibody titer from baseline.

[5] Rate difference=(NVSI-06-09)-(BBIBP-CorV). Rate difference and 95%CI were estimated by CMH method considering stratification factors.

[6] The serum sample of one subject was not tested due to contamination.

**Table S21: Live-virus neutralizing antibody response against Omicron (BA.4) variant (PPS)**

|  | **14 days after boosting** | | |
| --- | --- | --- | --- |
|  | **NVSI-06-09** | **BBIBP-CorV** | ***p* value** |
| **All subjects** |  |  |  |
| N(missing) | 255(1) ^[6]^ | 249 |  |
| Pre-booster antibody titer ≥4, n (%)  95%CI (%) | 248(97.64) | 244(97.99) | 0.7860 |
|  | 94.93-99.13 | 95.38-99.34 |  |
| Pre-booster antibody GMT^[1]^ (95%CI) | 40.59(35.75-46.10) | 42.14(36.80-48.25) | 0.6927 |
| Post-booster antibody GMT (95%CI) | 910.19(779.53-1062.75) | 113.51(98.50-130.81) | <0.0001 |
| Ratio of GMT between two groups(95%CI)^[2]^ | 8.02(6.50-9.89) |  |  |
| Post-booster adjusted antibody GMT (95%CI)^[3]^ | 989.13(760.96-1285.72) | 121.42(93.06-158.42) | <0.0001 |
| Ratio of adjusted GMT between two groups(95%CI)^[3]^ | 8.15(6.72-9.87) |  |  |
| Rate of 4-fold rise^[4]^, n (%) | 235(92.52) | 107(42.97) | <0.0001 |
| 95%CI (%) | 88.56-95.44 | 36.74-49.37 |  |
| Rate difference between two groups (%, 95%CI^[5]^) | 49.55(42.60-56.50) |  |  |
| Post-booster antibody GMT fold rise (95%CI) | 22.42(18.78-26.77) | 2.69(2.38-3.05) | <0.0001 |
| **All subjects (Excluding participants with positive**  **or weak positive COVID-19 PCR test)** |  |  |  |
| N(missing) | 231(1) ^[6]^ | 226(0) |  |
| Pre-booster antibody titer ≥4, n (%) | 227(98.70) | 221(97.79) | 0.5006 |
| 95%CI (%) | 96.24-99.73 | 94.91-99.28 |  |
| Pre-booster antibody GMT^[1]^ (95%CI) | 40.94(36.12-46.41) | 41.85(36.31-48.23) | 0.8187 |
| Post-booster antibody GMT (95%CI) | 919.13(780.10-1082.93) | 115.62(99.46-134.40) | <0.0001 |
| Ratio of GMT between two groups(95%CI)^[2]^ | 7.95(6.37-9.93) |  |  |
| Post-booster adjusted antibody GMT (95%CI)^[3]^ | 971.77(737.07-1281.21) | 121.19(91.17-161.10) | <0.0001 |
| Ratio of adjusted GMT between two groups(95%CI)^[3]^ | 8.02(6.54-9.83) |  |  |
| Rate of 4-fold rise^[4]^, n (%) | 215(93.48) | 97(42.92) | <0.0001 |
| 95%CI (%) | 89.47-96.30 | 36.38-49.65 |  |
| Rate difference between two groups (%, 95%CI^[5]^) | 50.59(43.39-57.79) |  |  |
| Post-booster antibody GMT fold rise (95%CI) | 22.45(18.69-26.97) | 2.76(2.42-3.15) | <0.0001 |
| **Subjects who have received 2 doses of inactivated vaccine previously** |  |  |  |
| N(missing) | 11(0) | 9(0) |  |
| Pre-booster antibody titer ≥4, n (%) | 11(100.00) | 9(100.00) | 1.0000 |
| 95%CI (%) | 71.51-100.00 | 66.37-100.00 |  |
| Pre-booster antibody GMT^[1]^ (95%CI) | 33.92(20.31-56.64) | 65.70(39.89-108.20) | 0.0544 |
| Post-booster antibody GMT (95%CI) | 1019.16(417.60-2487.27) | 155.29(75.79-318.21) | 0.0021 |
| Ratio of GMT between two groups(95%CI)^[2]^ | 6.56(2.18-19.80) |  |  |
| Post-booster adjusted antibody GMT (95%CI)^[3]^ | 1011.71(452.45-2262.24) | 156.69(63.77-385.04) | 0.0065 |
| Ratio of adjusted GMT between two groups(95%CI)^[3]^ | 6.46(1.82-22.94) |  |  |
| Rate of 4-fold rise^[4]^, n (%) | 10(90.91) | 4(44.44) | 0.0279 |
| 95%CI (%) | 58.72-99.77 | 13.70-78.80 |  |
| Rate difference between two groups (%, 95%CI^[5]^) | 46.46(9.82-83.10) |  |  |
| Post-booster antibody GMT fold rise (95%CI) | 30.05(10.05-89.82) | 2.36(1.11-5.02) | 0.0007 |
| **Subjects who have received 3 doses of inactivated vaccine previously** |  |  |  |
| N(missing) | 244(1) ^[6]^ | 240(0) |  |
| Pre-booster antibody titer ≥4, n (%) | 237(97.53) | 235(97.92) | 0.7763 |
| 95%CI (%) | 94.70-99.09 | 95.21-99.32 |  |
| Pre-booster antibody GMT^[1]^ (95%CI) | 40.93(35.88-46.68) | 41.44(36.05-47.64) | 0.8977 |
| Post-booster antibody GMT (95%CI) | 905.54(772.92-1060.92) | 112.19(97.01-129.74) | <0.0001 |
| Ratio of GMT between two groups(95%CI)^[2]^ | 8.07(6.51-10.00) |  |  |
| Post-booster adjusted antibody GMT (95%CI)^[3]^ | 908.16(791.25-1042.36) | 111.86(97.37-128.50) | <0.0001 |
| Ratio of adjusted GMT between two groups(95%CI)^[3]^ | 8.12(6.68-9.87) |  |  |
| Rate of 4-fold rise^[4]^, n (%) | 225(92.59) | 103(42.92) | <0.0001 |
| 95%CI (%) | 88.55-95.55 | 36.57-49.44 |  |
| Rate difference between two groups (%, 95%CI^[5]^) | 49.68(42.60-56.75) |  |  |
| Post-booster antibody GMT fold rise (95%CI) | 22.13(18.48-26.50) | 2.71(2.38-3.07) | <0.0001 |

Notes: [1] GMT represent geometric mean titer.

[2] The ratio of GMT between two groups was calculated by “NVSI-06-09/ BBIBP-CorV”.

[3] Covariance analysis with least square method was used to calculate the adjusted GMT and the corresponding *p* value.

[4] Rate of 4-fold rise was defined as percentage of participants with a ≥4-fold rise in neutralizing antibody titer from baseline.

[5] Rate difference=(NVSI-06-09)-(BBIBP-CorV). Rate difference and 95%CI were estimated by CMH method considering stratification factors.

[6] The serum sample of one subject was not tested due to contamination.

**Table S22: Live-virus neutralizing antibody response against Omicron (BA.5) variant (PPS)**

|  | **14 days after boosting** | | |
| --- | --- | --- | --- |
|  | **NVSI-06-09** | **BBIBP-CorV** | ***p* value** |
| **All subjects** |  |  |  |
| N(missing) | 255(1) ^[6]^ | 249(0) |  |
| Pre-booster antibody titer ≥4, n (%) | 248(97.64) | 238(95.58) | 0.2021 |
| 95%CI (%) | 94.93-99.13 | 92.23-97.77 |  |
| Pre-booster antibody GMT^[1]^ (95%CI) | 36.95(32.38-42.16) | 34.98(30.53-40.09) | 0.5707 |
| Post-booster antibody GMT (95%CI) | 999.85(872.41-1145.90) | 165.39(142.99-191.30) | <0.0001 |
| Ratio of GMT between two groups(95%CI)^[2]^ | 6.05(4.96-7.38) |  |  |
| Post-booster adjusted antibody GMT (95%CI)^[3]^ | 910.53(708.86-1169.57) | 153.82(119.34-198.27) | <0.0001 |
| Ratio of adjusted GMT between two groups(95%CI)^[3]^ | 5.92(4.93-7.11) |  |  |
| Rate of 4-fold rise^[4]^, n (%) | 241(94.88) | 147(59.04) | <0.0001 |
| 95%CI (%) | 91.41-97.25 | 52.65-65.20 |  |
| Rate difference between two groups (%, 95%CI^[5]^) | 35.95(29.28-42.62) |  |  |
| Post-booster antibody GMT fold rise (95%CI) | 27.06(23.04-31.78) | 4.73(4.10-5.45) | <0.0001 |
| **All subjects (Excluding participants with positive**  **or weak positive COVID-19 PCR test)** |  |  |  |
| N(missing) | 231(1) ^[6]^ | 226(0) |  |
| Pre-booster antibody titer ≥4, n (%) | 226(98.26) | 215(95.13) | 0.0611 |
| 95%CI (%) | 95.61-99.52 | 91.46-97.55 |  |
| Pre-booster antibody GMT^[1]^ (95%CI) | 37.60(32.85-43.03) | 34.73(30.03-40.15) | 0.4300 |
| Post-booster antibody GMT (95%CI) | 999.29(864.82-1154.67) | 166.48(142.50-194.49) | <0.0001 |
| Ratio of GMT between two groups(95%CI)^[2]^ | 6.00(4.86-7.42) |  |  |
| Post-booster adjusted antibody GMT (95%CI)^[3]^ | 902.05(692.51-1174.98) | 154.95(118.03-203.42) | <0.0001 |
| Ratio of adjusted GMT between two groups(95%CI)^[3]^ | 5.82(4.79-7.07) |  |  |
| Rate of 4-fold rise^[4]^, n (%) | 218(94.78) | 136(60.18) | <0.0001 |
| 95%CI (%) | 91.06-97.28 | 53.47-66.61 |  |
| Rate difference between two groups (%, 95%CI^[5]^) | 34.77(27.78-41.76) |  |  |
| Post-booster antibody GMT fold rise (95%CI) | 26.58(22.48-31.42) | 4.79(4.12-5.57) | <0.0001 |
| **Subjects who have received 2 doses of inactivated vaccine previously** |  |  |  |
| N(missing) | 11(0) | 9(0) |  |
| Pre-booster antibody titer ≥4, n (%) | 11(100.00) | 9(100.00) | 1.0000 |
| 95%CI (%) | 71.51-100.00 | 66.37-100.00 |  |
| Pre-booster antibody GMT^[1]^ (95%CI) | 28.08(17.30-45.56) | 61.63(30.32-125.26) | 0.0461 |
| Post-booster antibody GMT (95%CI) | 908.15(415.39-1985.47) | 139.04(72.29-267.42) | 0.0008 |
| Ratio of GMT between two groups(95%CI)^[2]^ | 6.53(2.45-17.39) |  |  |
| Post-booster adjusted antibody GMT (95%CI)^[3]^ | 950.99(466.28-1939.58) | 131.43(59.24-291.59) | 0.0018 |
| Ratio of adjusted GMT between two groups(95%CI)^[3]^ | 7.24(2.34-22.34) |  |  |
| Rate of 4-fold rise^[4]^, n (%) | 10(90.91) | 3(33.33) | 0.0089 |
| 95%CI (%) | 58.72-99.77 | 7.49-70.07 |  |
| Rate difference between two groups (%, 95%CI^[5]^) | 57.58(22.40-92.75) |  |  |
| Post-booster antibody GMT fold rise (95%CI) | 32.34(11.97-87.37) | 2.26(1.14-4.47) | 0.0002 |
| **Subjects who have received 3 doses of inactivated vaccine previously** |  |  |  |
| N(missing) | 244(1) ^[6]^ | 240(0) |  |
| Pre-booster antibody titer ≥4, n (%) | 237(97.53) | 229(95.42) | 0.2074 |
| 95%CI (%) | 94.70-99.09 | 91.95-97.69 |  |
| Pre-booster antibody GMT^[1]^ (95%CI) | 37.41(32.64-42.88) | 34.25(29.81-39.35) | 0.3724 |
| Post-booster antibody GMT (95%CI) | 1004.21(873.58-1154.38) | 166.47(143.33-193.34) | <0.0001 |
| Ratio of GMT between two groups(95%CI)^[2]^ | 6.03(4.92-7.40) |  |  |
| Post-booster adjusted antibody GMT (95%CI)^[3]^ | 985.96(864.03-1125.11) | 169.59(148.49-193.68) | <0.0001 |
| Ratio of adjusted GMT between two groups(95%CI)^[3]^ | 5.81(4.82-7.01) |  |  |
| Rate of 4-fold rise^[4]^, n (%) | 231(95.06) | 144(60.00) | <0.0001 |
| 95%CI (%) | 91.53-97.42 | 53.50-66.25 |  |
| Rate difference between two groups (%, 95%CI^[5]^) | 35.06(28.29-41.83) |  |  |
| Post-booster antibody GMT fold rise (95%CI) | 26.84(22.79-31.61) | 4.86(4.20-5.62) | <0.0001 |

Notes: [1] GMT represent geometric mean titer.

[2] The ratio of GMT between two groups was calculated by “NVSI-06-09/ BBIBP-CorV”.

[3] Covariance analysis with least square method was used to calculate the adjusted GMT and the corresponding *p* value.

[4] Rate of 4-fold rise was defined as percentage of participants with a ≥4-fold rise in neutralizing antibody titer from baseline.

[5] Rate difference=(NVSI-06-09)-(BBIBP-CorV). Rate difference and 95%CI were estimated by CMH method considering stratification factors.

[6] The serum sample of one subject was not tested due to contamination.

**Table S23:** **Comparison of the neutralizing antibody response against Omicron (BA.2) variant elicited by NVSI-06-09 booster between subjects primed with two and three doses of inactivated vaccine (PPS)**

|  | **NVSI-06-09** | | |
| --- | --- | --- | --- |
|  | **2 doses** | **3 doses** | ***p* value** |
| **All subjects** |  |  |  |
| N(missing) | 11(0) | 244(1) ^[6]^ |  |
| Pre-booster antibody titer ≥4, n (%) | 11(100.00) | 235(96.71) | 1.0000 |
| 95%CI (%) | 71.51-100.00 | 93.62-98.57 |  |
| Pre-booster antibody GMT^[1]^ (95%CI) | 28.69(16.55-49.72) | 39.75(34.61-45.66) | 0.3308 |
| Post-booster antibody GMT (95%CI) | 724.52(416.59-1260.06) | 997.05(859.99-1155.95) | 0.3721 |
| Ratio of GMT between two groups(95%CI)^[2]^ | 0.73(0.36-1.47) |  |  |
| Post-booster adjusted antibody GMT (95%CI)^[3]^ | 786.99(403.16-1536.24) | 993.32(861.77-1144.96) | 0.5031 |
| Ratio of adjusted GMT between two groups(95%CI)^[3]^ | 0.79(0.40-1.57) |  |  |
| Rate of 4-fold rise^[4]^, n (%) | 11(100.0) | 229(94.24) | 0.4137 |
| 95%CI (%) | 71.51-100.00 | 90.52-96.81 |  |
| Rate difference between two groups (%, 95%CI^[5]^) | 5.76(2.83-8.69) |  |  |
| Post-booster antibody GMT fold rise (95%CI) | 25.26(11.26-56.65) | 25.08(21.06-29.88) | 0.9869 |
| **All subjects (Excluding participants with positive**  **or weak positive COVID-19 PCR test)** |  |  |  |
| N(missing) | 11(0) | 220(1) ^[6]^ |  |
| Pre-booster antibody titer ≥4, n (%) | 11(100.00) | 213(97.26) | 1.0000 |
| 95%CI (%) | 71.51-100.00 | 94.13-98.99 |  |
| Pre-booster antibody GMT^[1]^ (95%CI) | 28.69(16.55-49.72) | 41.05(35.59-47.35) | 0.2759 |
| Post-booster antibody GMT (95%CI) | 724.52(416.59-1260.06) | 1001.17(855.96-1171.01) | 0.3692 |
| Ratio of GMT between two groups(95%CI)^[2]^ | 0.72(0.36-1.47) |  |  |
| Post-booster adjusted antibody GMT (95%CI)^[3]^ | 791.93(403.43-1554.57) | 996.70(857.18-1158.94) | 0.5128 |
| Ratio of adjusted GMT between two groups(95%CI)^[3]^ | 0.79(0.40-1.59) |  |  |
| Rate of 4-fold rise^[4]^, n (%) | 11(100.0) | 206(94.06) | 0.4065 |
| 95%CI (%) | 71.51-100.00 | 90.06-96.80 |  |
| Rate difference between two groups (%, 95%CI^[5]^) | 5.94(2.81-9.07) |  |  |
| Post-booster antibody GMT fold rise (95%CI) | 25.26(11.26-56.65) | 24.39(20.29-29.32) | 0.9345 |

Notes: [1] GMT represent geometric mean titer.

[2] The ratio of GMT between two groups was calculated by “NVSI-06-09/ BBIBP-CorV”.

[3] Covariance analysis with least square method was used to calculate the adjusted GMT and the corresponding *p* value.

[4] Rate of 4-fold rise was defined as percentage of participants with a ≥4-fold rise in neutralizing antibody titer from baseline.

[5] Rate difference=(NVSI-06-09)-(BBIBP-CorV). Rate difference and 95%CI were estimated by CMH method considering stratification factors.

[6] The serum sample of one subject was not tested due to contamination.

**Table S24: Comparison of the neutralizing antibody response against Omicron (BA.2) variant elicited by BBIBP-CorV booster between subjects primed with two and three doses of inactivated vaccine (PPS)**

|  | **BBIBP-CorV** | | |
| --- | --- | --- | --- |
|  | **2 doses** | **3 doses** | ***p* value** |
| **All subjects** |  |  |  |
| N(missing) | 9(0) | 240(0) |  |
| Pre-booster antibody titer ≥4, n (%) | 9(100.00) | 232(96.67) | 1.0000 |
| 95%CI (%) | 66.37-100.00 | 93.54-98.55 |  |
| Pre-booster antibody GMT^[1]^ (95%CI) | 80.17(53.95-119.13) | 39.32(34.03-45.44) | 0.0628 |
| Post-booster antibody GMT (95%CI) | 153.28(79.97-293.80) | 116.16(101.71-132.66) | 0.4324 |
| Ratio of GMT between two groups(95%CI)^[2]^ | 1.32(0.66-2.64) |  |  |
| Post-booster adjusted antibody GMT (95%CI)^[3]^ | 100.12(60.09-166.84) | 118.03(106.98-130.21) | 0.5340 |
| Ratio of adjusted GMT between two groups(95%CI)^[3]^ | 0.85(0.50-1.43) |  |  |
| Rate of 4-fold rise^[4]^, n (%) | 2(22.22) | 102(42.50) | 0.2268 |
| 95%CI (%) | 2.81-60.01 | 36.16-49.02 |  |
| Rate difference between two groups (%, 95%CI^[5]^) | -20.28(-48.15-7.59) |  |  |
| Post-booster antibody GMT fold rise (95%CI) | 1.91(1.00-3.65) | 2.95(2.64-3.30) | 0.1471 |
| **All subjects (Excluding participants with positive**  **or weak positive COVID-19 PCR test)** |  |  |  |
| N(missing) | 7(0) | 219(0) |  |
| Pre-booster antibody titer ≥4, n (%) | 7(100.00) | 211(96.35) | 1.0000 |
| 95%CI (%) | 59.04-100.00 | 92.93-98.41 |  |
| Pre-booster antibody GMT^[1]^ (95%CI) | 66.19(46.40-94.42) | 39.90(34.30-46.43) | 0.2421 |
| Post-booster antibody GMT (95%CI) | 124.93(56.93-274.15) | 118.40(103.12-135.95) | 0.8924 |
| Ratio of GMT between two groups(95%CI)^[2]^ | 1.06(0.48-2.31) |  |  |
| Post-booster adjusted antibody GMT (95%CI)^[3]^ | 93.57(51.68-169.40) | 119.50(107.50-132.84) | 0.4250 |
| Ratio of adjusted GMT between two groups(95%CI)^[3]^ | 0.78(0.43-1.43) |  |  |
| Rate of 4-fold rise^[4]^, n (%) | 2(28.57) | 96(43.84) | 0.4235 |
| 95%CI (%) | 3.67-70.96 | 37.16-50.68 |  |
| Rate difference between two groups (%, 95%CI^[5]^) | -15.26(-49.37-18.84) |  |  |
| Post-booster antibody GMT fold rise (95%CI) | 1.89(0.77-4.64) | 2.97(2.63-3.35) | 0.2003 |

Notes: [1] GMT represent geometric mean titer.

[2] The ratio of GMT between two groups was calculated by “NVSI-06-09/ BBIBP-CorV”.

[3] Covariance analysis with least square method was used to calculate the adjusted GMT and the corresponding *p* value.

[4] Rate of 4-fold rise was defined as percentage of participants with a ≥4-fold rise in neutralizing antibody titer from baseline.

[5] Rate difference=(NVSI-06-09)-(BBIBP-CorV). Rate difference and 95%CI were estimated by CMH method considering stratification factors.

**Table S25:** **Comparison of the neutralizing antibody response against Omicron (BA.4) variant elicited by NVSI-06-09 booster between subjects primed with two and three doses of inactivated vaccine (PPS)**

|  | **NVSI-06-09** | | |
| --- | --- | --- | --- |
|  | **2 doses** | **3 doses** | ***p* value** |
| **All subjects** |  |  |  |
| N(missing) | 11(0) | 244(1) ^[6]^ |  |
| Pre-booster antibody titer ≥4, n (%) | 11(100.00) | 237(97.53) | 1.0000 |
| 95%CI (%) | 71.51-100.00 | 94.70-99.09 |  |
| Pre-booster antibody GMT^[1]^ (95%CI) | 33.92(20.31-56.64) | 40.93(35.88-46.68) | 0.5551 |
| Post-booster antibody GMT (95%CI) | 1019.16(417.60-2487.27) | 905.54(772.92-1060.92) | 0.7605 |
| Ratio of GMT between two groups(95%CI)^[2]^ | 1.13(0.52-2.41) |  |  |
| Post-booster adjusted antibody GMT (95%CI)^[3]^ | 1070.31(516.30-2218.79) | 903.54(773.80-1055.03) | 0.6549 |
| Ratio of adjusted GMT between two groups(95%CI)^[3]^ | 1.18(0.56-2.50) |  |  |
| Rate of 4-fold rise^[4]^, n (%) | 10(90.91) | 225(92.59) | 0.8359 |
| 95%CI (%) | 58.72-99.77 | 88.55-95.55 |  |
| Rate difference between two groups (%, 95%CI^[5]^) | -1.68(-18.99-15.62) |  |  |
| Post-booster antibody GMT fold rise (95%CI) | 30.05(10.05-89.82) | 22.13(18.48-26.50) | 0.4900 |
| **All subjects (Excluding participants with positive**  **or weak positive COVID-19 PCR test)** |  |  |  |
| N(missing) | 11(0) | 220(1) ^[6]^ |  |
| Pre-booster antibody titer ≥4, n (%) | 11(100.00) | 216(98.63) | 1.0000 |
| 95%CI (%) | 71.51-100.00 | 96.05-99.72 |  |
| Pre-booster antibody GMT^[1]^ (95%CI) | 33.92(20.31-56.64) | 41.33(36.30-47.05) | 0.5088 |
| Post-booster antibody GMT (95%CI) | 1019.16(417.60-2487.27) | 914.37(772.92-1081.70) | 0.7816 |
| Ratio of GMT between two groups(95%CI)^[2]^ | 1.11(0.52-2.41) |  |  |
| Post-booster adjusted antibody GMT (95%CI)^[3]^ | 1075.69(515.56-2244.40) | 911.89(773.43-1075.14) | 0.6662 |
| Ratio of adjusted GMT between two groups(95%CI)^[3]^ | 1.18(0.56-2.51) |  |  |
| Rate of 4-fold rise^[4]^, n (%) | 10(90.91) | 205(93.61) | 0.7242 |
| 95%CI (%) | 58.72-99.77 | 89.51-96.46 |  |
| Rate difference between two groups (%, 95%CI^[5]^) | -2.70(-19.99-14.60) |  |  |
| Post-booster antibody GMT fold rise (95%CI) | 30.05(10.05-89.82) | 22.12(18.36-26.67) | 0.4842 |

Notes: [1] GMT represent geometric mean titer.

[2] The ratio of GMT between two groups was calculated by “NVSI-06-09/ BBIBP-CorV”.

[3] Covariance analysis with least square method was used to calculate the adjusted GMT and the corresponding *p* value.

[4] Rate of 4-fold rise was defined as percentage of participants with a ≥4-fold rise in neutralizing antibody titer from baseline.

[5] Rate difference=(NVSI-06-09)-(BBIBP-CorV). Rate difference and 95%CI were estimated by CMH method considering stratification factors.

[6] The serum sample of one subject was not tested due to contamination.

**Table S26: Comparison of the neutralizing antibody response against Omicron (BA.4) variant elicited by BBIBP-CorV booster between subjects primed with two and three doses of inactivated vaccine (PPS)**

|  | **BBIBP-CorV** | | |
| --- | --- | --- | --- |
|  | **2 doses** | **3 doses** | ***p* value** |
| **All subjects** |  |  |  |
| N(missing) | 9(0) | 240(0) |  |
| Pre-booster antibody titer ≥4, n (%) | 9(100.00) | 235(97.92) | 1.0000 |
| 95%CI (%) | 66.37-100.00 | 95.21-99.32 |  |
| Pre-booster antibody GMT^[1]^ (95%CI) | 65.70(39.89-108.20) | 41.44(36.05-47.64) | 0.2118 |
| Post-booster antibody GMT (95%CI) | 155.29(75.79-318.21) | 112.19(97.01-129.74) | 0.4004 |
| Ratio of GMT between two groups(95%CI)^[2]^ | 1.38(0.65-2.96) |  |  |
| Post-booster adjusted antibody GMT (95%CI)^[3]^ | 117.56(64.42-214.55) | 113.36(100.93-127.33) | 0.9070 |
| Ratio of adjusted GMT between two groups(95%CI)^[3]^ | 1.04(0.56-1.91) |  |  |
| Rate of 4-fold rise^[4]^, n (%) | 4(44.44) | 103(42.92) | 0.9277 |
| 95%CI (%) | 13.70-78.80 | 36.57-49.44 |  |
| Rate difference between two groups (%, 95%CI^[5]^) | 1.53(-31.53-34.59) |  |  |
| Post-booster antibody GMT fold rise (95%CI) | 2.36(1.11-5.02) | 2.71(2.38-3.07) | 0.6892 |
| **All subjects (Excluding participants with positive**  **or weak positive COVID-19 PCR test)** |  |  |  |
| N(missing) | 7(0) | 219(0) |  |
| Pre-booster antibody titer ≥4, n (%) | 7(100.00) | 214(97.72) | 1.0000 |
| 95%CI (%) | 59.04-100.00 | 94.75-99.25 |  |
| Pre-booster antibody GMT^[1]^ (95%CI) | 58.95(30.65-113.38) | 41.39(35.79-47.87) | 0.3962 |
| Post-booster antibody GMT (95%CI) | 132.38(52.81-331.87) | 115.12(98.71-134.26) | 0.7521 |
| Ratio of GMT between two groups(95%CI)^[2]^ | 1.15(0.48-2.75) |  |  |
| Post-booster adjusted antibody GMT (95%CI)^[3]^ | 106.48(53.44-212.17) | 115.92(102.50-131.11) | 0.8112 |
| Ratio of adjusted GMT between two groups(95%CI)^[3]^ | 0.92(0.46-1.85) |  |  |
| Rate of 4-fold rise^[4]^, n (%) | 3(42.86) | 94(42.92) | 0.9973 |
| 95%CI (%) | 9.90-81.59 | 36.27-49.76 |  |
| Rate difference between two groups (%, 95%CI^[5]^) | -0.07(-37.31-37.18) |  |  |
| Post-booster antibody GMT fold rise (95%CI) | 2.25(0.81-6.26) | 2.78(2.43-3.18) | 0.5793 |

Notes: [1] GMT represent geometric mean titer.

[2] The ratio of GMT between two groups was calculated by “NVSI-06-09/ BBIBP-CorV”.

[3] Covariance analysis with least square method was used to calculate the adjusted GMT and the corresponding *p* value.

[4] Rate of 4-fold rise was defined as percentage of participants with a ≥4-fold rise in neutralizing antibody titer from baseline.

[5] Rate difference=(NVSI-06-09)-(BBIBP-CorV). Rate difference and 95%CI were estimated by CMH method considering stratification factors.

**Table S27: Comparison of the neutralizing antibody response against Omicron (BA.5) variant elicited by NVSI-06-09 booster between subjects primed with two and three doses of inactivated vaccine (PPS)**

|  | **NVSI-06-09** | | |
| --- | --- | --- | --- |
|  | **2 doses** | **3 doses** | ***p* value** |
| **All subjects** |  |  |  |
| N(missing) | 11(0) | 244(1) ^[6]^ |  |
| Pre-booster antibody titer ≥4, n (%) | 11(100.00) | 237(97.53) | 1.0000 |
| 95%CI (%) | 71.51-100.00 | 94.70-99.09 |  |
| Pre-booster antibody GMT^[1]^ (95%CI) | 28.08(17.30-45.56) | 37.41(32.64-42.88) | 0.3845 |
| Post-booster antibody GMT (95%CI) | 908.15(415.39-1985.47) | 1004.21(873.58-1154.38) | 0.7682 |
| Ratio of GMT between two groups(95%CI)^[2]^ | 0.90(0.46-1.77) |  |  |
| Post-booster adjusted antibody GMT (95%CI)^[3]^ | 984.14(523.24-1851.00) | 1000.57(874.89-1144.29) | 0.9598 |
| Ratio of adjusted GMT between two groups(95%CI)^[3]^ | 0.98(0.52-1.88) |  |  |
| Rate of 4-fold rise^[4]^, n (%) | 10(90.91) | 231(95.06) | 0.5418 |
| 95%CI (%) | 58.72-99.77 | 91.53-97.42 |  |
| Rate difference between two groups (%, 95%CI^[5]^) | -4.15(-21.36-13.05) |  |  |
| Post-booster antibody GMT fold rise (95%CI) | 32.34(11.97-87.37) | 26.84(22.79-31.61) | 0.6427 |
| **All subjects (Excluding participants with positive**  **or weak positive COVID-19 PCR test)** |  |  |  |
| N(missing) | 11(0) | 220(1) ^[6]^ |  |
| Pre-booster antibody titer ≥4, n (%) | 11(100.00) | 215(98.17) | 1.0000 |
| 95%CI (%) | 71.51-100.00 | 95.39-99.50 |  |
| Pre-booster antibody GMT^[1]^ (95%CI) | 28.08(17.30-45.56) | 38.15(33.17-43.89) | 0.3408 |
| Post-booster antibody GMT (95%CI) | 908.15(415.39-1985.47) | 1004.10(865.85-1164.43) | 0.7708 |
| Ratio of GMT between two groups(95%CI)^[2]^ | 0.90(0.46-1.78) |  |  |
| Post-booster adjusted antibody GMT (95%CI)^[3]^ | 992.60(524.67-1877.86) | 999.63(866.75-1152.87) | 0.9831 |
| Ratio of adjusted GMT between two groups(95%CI)^[3]^ | 0.99(0.52-1.91) |  |  |
| Rate of 4-fold rise^[4]^, n (%) | 10(90.91) | 208(94.98) | 0.5547 |
| 95%CI (%) | 58.72-99.77 | 91.19-97.47 |  |
| Rate difference between two groups (%, 95%CI^[5]^) | -4.07(-21.30-13.17) |  |  |
| Post-booster antibody GMT fold rise (95%CI) | 32.34(11.97-87.37) | 26.32(22.19-31.21) | 0.6055 |

Notes: [1] GMT represent geometric mean titer.

[2] The ratio of GMT between two groups was calculated by “NVSI-06-09/ BBIBP-CorV”.

[3] Covariance analysis with least square method was used to calculate the adjusted GMT and the corresponding *p* value.

[4] Rate of 4-fold rise was defined as percentage of participants with a ≥4-fold rise in neutralizing antibody titer from baseline.

[5] Rate difference=(NVSI-06-09)-(BBIBP-CorV). Rate difference and 95%CI were estimated by CMH method considering stratification factors.

[6] The serum sample of one subject was not tested due to contamination.

**Table S28: Comparison of the neutralizing antibody response against Omicron (BA.5) variant elicited by BBIBP-CorV booster between subjects primed with two and three doses of inactivated vaccine (PPS)**

|  | **BBIBP-CorV** | | |
| --- | --- | --- | --- |
|  | **2 doses** | **3 doses** | ***p* value** |
| **All subjects** |  |  |  |
| N(missing) | 9(0) | 240(0) |  |
| Pre-booster antibody titer ≥4, n (%) | 9(100.00) | 229(95.42) | 1.0000 |
| 95%CI (%) | 66.37-100.00 | 91.95-97.69 |  |
| Pre-booster antibody GMT^[1]^ (95%CI) | 61.63(30.32-125.26) | 34.25(29.81-39.35) | 0.1129 |
| Post-booster antibody GMT (95%CI) | 139.04(72.29-267.42) | 166.47(143.33-193.34) | 0.6502 |
| Ratio of GMT between two groups(95%CI)^[2]^ | 0.84(0.38-1.82) |  |  |
| Post-booster adjusted antibody GMT (95%CI)^[3]^ | 103.01(52.63-201.60) | 168.35(147.92-191.61) | 0.1585 |
| Ratio of adjusted GMT between two groups(95%CI)^[3]^ | 0.61(0.31-1.21) |  |  |
| Rate of 4-fold rise^[4]^, n (%) | 3(33.33) | 144(60.00) | 0.1110 |
| 95%CI (%) | 7.49-70.07 | 53.50-66.25 |  |
| Rate difference between two groups (%, 95%CI^[5]^) | -26.67(-58.08-4.75) |  |  |
| Post-booster antibody GMT fold rise (95%CI) | 2.26(1.14-4.47) | 4.86(4.20-5.62) | 0.0479 |
| **All subjects (Excluding participants with positive**  **or weak positive COVID-19 PCR test)** |  |  |  |
| N(missing) | 7(0) | 219(0) |  |
| Pre-booster antibody titer ≥4, n (%) | 7(100.00) | 208(94.98) | 1.0000 |
| 95%CI (%) | 59.04-100.00 | 91.19-97.47 |  |
| Pre-booster antibody GMT^[1]^ (95%CI) | 60.97(22.76-163.28) | 34.11(29.43-39.52) | 0.1726 |
| Post-booster antibody GMT (95%CI) | 126.80(52.21-307.97) | 167.94(143.25-196.88) | 0.5385 |
| Ratio of GMT between two groups(95%CI)^[2]^ | 0.76(0.31-1.86) |  |  |
| Post-booster adjusted antibody GMT (95%CI)^[3]^ | 93.46(43.32-201.62) | 169.58(147.88-194.47) | 0.1342 |
| Ratio of adjusted GMT between two groups(95%CI)^[3]^ | 0.55(0.25-1.20) |  |  |
| Rate of 4-fold rise^[4]^, n (%) | 3(42.86) | 133(60.73) | 0.3427 |
| 95%CI (%) | 9.90-81.59 | 53.92-67.24 |  |
| Rate difference between two groups (%, 95%CI^[5]^) | -17.87(-55.10-19.35) |  |  |
| Post-booster antibody GMT fold rise (95%CI) | 2.08(0.82-5.30) | 4.92(4.23-5.74) | 0.0510 |

Notes: [1] GMT represent geometric mean titer.

[2] The ratio of GMT between two groups was calculated by “NVSI-06-09/ BBIBP-CorV”.

[3] Covariance analysis with least square method was used to calculate the adjusted GMT and the corresponding *p* value.

[4] Rate of 4-fold rise was defined as percentage of participants with a ≥4-fold rise in neutralizing antibody titer from baseline.

[5] Rate difference=(NVSI-06-09)-(BBIBP-CorV). Rate difference and 95%CI were estimated by CMH method considering stratification factors.

**Table S29: Comparison of the neutralizing antibody response against Omicron (BA.2) variant elicited by NVSI-06-09 booster between male and female subjects (PPS)**

|  | **NVSI-06-09** | | |
| --- | --- | --- | --- |
|  | **Male** | **Female** | ***p* value** |
| **All subjects** |  |  |  |
| N(missing) | 239(1) ^[6]^ | 16(0) |  |
| Pre-booster antibody titer ≥4, n (%) | 232(97.48) | 14(87.50) | 0.0835 |
| 95%CI (%) | 94.59-99.07 | 61.65-98.45 |  |
| Pre-booster antibody GMT^[1]^ (95%CI) | 40.72(35.57-46.60) | 22.24(10.71-46.18) | 0.0309 |
| Post-booster antibody GMT (95%CI) | 990.92(853.62-1150.30) | 877.44(508.27-1514.77) | 0.6851 |
| Ratio of GMT between two groups(95%CI)^[2]^ | 1.13(0.63-2.04) |  |  |
| Post-booster adjusted antibody GMT (95%CI)^[3]^ | 878.00(619.33-1244.71) | 932.79(502.95-1729.98) | 0.8376 |
| Ratio of adjusted GMT between two groups(95%CI)^[3]^ | 0.94(0.53-1.68) |  |  |
| Rate of 4-fold rise^[4]^, n (%) | 224(94.12) | 16(100.0) | 0.3416 |
| 95%CI (%) | 90.33-96.75 | 79.41-100.00 |  |
| Rate difference between two groups (%, 95%CI^[5]^) | -5.44(-8.54--2.34) |  |  |
| Post-booster antibody GMT fold rise (95%CI) | 24.34(20.46-28.95) | 39.45(17.07-91.15) | 0.1742 |
| **All subjects (Excluding participants with positive**  **or weak positive COVID-19 PCR test)** |  |  |  |
| N(missing) | 216(1) ^[6]^ | 15(0) |  |
| Pre-booster antibody titer ≥4, n (%) | 210(97.67) | 14(93.33) | 0.3359 |
| 95%CI (%) | 94.66-99.24 | 68.05-99.83 |  |
| Pre-booster antibody GMT^[1]^ (95%CI) | 41.60(36.15-47.87) | 26.12(13.04-52.30) | 0.1011 |
| Post-booster antibody GMT (95%CI) | 983.67(839.12-1153.12) | 1016.91(629.12-1643.74) | 0.9150 |
| Ratio of GMT between two groups(95%CI)^[2]^ | 0.97(0.52-1.79) |  |  |
| Post-booster adjusted antibody GMT (95%CI)^[3]^ | 870.02(611.20-1238.44) | 1041.01(551.49-1965.03) | 0.5584 |
| Ratio of adjusted GMT between two groups(95%CI)^[3]^ | 0.84(0.46-1.53) |  |  |
| Rate of 4-fold rise^[4]^, n (%) | 202(93.95) | 15(100.0) | 0.3515 |
| 95%CI (%) | 89.88-96.74 | 78.20-100.00 |  |
| Rate difference between two groups (%, 95%CI^[5]^) | -5.57(-8.86--2.27) |  |  |
| Post-booster antibody GMT fold rise (95%CI) | 23.65(19.72-28.36) | 38.94(15.82-95.81) | 0.1744 |

Notes: [1] GMT represent geometric mean titer.

[2] The ratio of GMT between two groups was calculated by “NVSI-06-09/ BBIBP-CorV”.

[3] Covariance analysis with least square method was used to calculate the adjusted GMT and the corresponding *p* value.

[4] Rate of 4-fold rise was defined as percentage of participants with a ≥4-fold rise in neutralizing antibody titer from baseline.

[5] Rate difference=(NVSI-06-09)-(BBIBP-CorV). Rate difference and 95%CI were estimated by CMH method considering stratification factors.

[6] The serum sample of one subject was not tested due to contamination.

**Table S30: Comparison of the neutralizing antibody response against Omicron (BA.2) variant elicited by BBIBP-CorV booster between male and female subjects (PPS)**

|  | **BBIBP-CorV** | | |
| --- | --- | --- | --- |
|  | **Male** | **Female** | ***p* value** |
| **All subjects** |  |  |  |
| N(missing) | 237(0) | 12(0) |  |
| Pre-booster antibody titer ≥4, n (%) | 230(97.05) | 11(91.67) | 0.3303 |
| 95%CI (%) | 94.01-98.80 | 61.52-99.79 |  |
| Pre-booster antibody GMT^[1]^ (95%CI) | 40.51(35.06-46.79) | 37.36(17.60-79.31) | 0.8090 |
| Post-booster antibody GMT (95%CI) | 115.08(100.80-131.39) | 171.80(88.17-334.73) | 0.1926 |
| Ratio of GMT between two groups(95%CI)^[2]^ | 0.67(0.37-1.23) |  |  |
| Post-booster adjusted antibody GMT (95%CI)^[3]^ | 107.47(82.98-139.19) | 167.79(101.03-278.68) | 0.0513 |
| Ratio of adjusted GMT between two groups(95%CI)^[3]^ | 0.64(0.41-1.00) |  |  |
| Rate of 4-fold rise^[4]^, n (%) | 97(40.93) | 7(58.33) | 0.2560 |
| 95%CI (%) | 34.61-47.48 | 27.67-84.83 |  |
| Rate difference between two groups (%, 95%CI^[5]^) | -16.67(-45.29-11.95) |  |  |
| Post-booster antibody GMT fold rise (95%CI) | 2.84(2.54-3.18) | 4.60(2.91-7.27) | 0.0652 |
| **All subjects (Excluding participants with positive**  **or weak positive COVID-19 PCR test)** |  |  |  |
| N(missing) | 216(0) | 10(0) |  |
| Pre-booster antibody titer ≥4, n (%) | 209(96.76) | 9(90.00) | 0.3078 |
| 95%CI (%) | 93.44-98.69 | 55.50-99.75 |  |
| Pre-booster antibody GMT^[1]^ (95%CI) | 40.52(34.87-47.09) | 40.81(16.22-102.68) | 0.9843 |
| Post-booster antibody GMT (95%CI) | 115.36(100.53-132.38) | 215.42(105.30-440.68) | 0.0609 |
| Ratio of GMT between two groups(95%CI)^[2]^ | 0.54(0.28-1.03) |  |  |
| Post-booster adjusted antibody GMT (95%CI)^[3]^ | 104.28(77.38-140.55) | 192.55(108.49-341.75) | 0.0168 |
| Ratio of adjusted GMT between two groups(95%CI)^[3]^ | 0.54(0.33-0.89) |  |  |
| Rate of 4-fold rise^[4]^, n (%) | 91(42.13) | 7(70.00) | 0.0886 |
| 95%CI (%) | 35.46-49.02 | 34.75-93.33 |  |
| Rate difference between two groups (%, 95%CI^[5]^) | -27.42(-56.60-1.77) |  |  |
| Post-booster antibody GMT fold rise (95%CI) | 2.85(2.52-3.22) | 5.28(3.18-8.77) | 0.0375 |

Notes: [1] GMT represent geometric mean titer.

[2] The ratio of GMT between two groups was calculated by “NVSI-06-09/ BBIBP-CorV”.

[3] Covariance analysis with least square method was used to calculate the adjusted GMT and the corresponding *p* value.

[4] Rate of 4-fold rise was defined as percentage of participants with a ≥4-fold rise in neutralizing antibody titer from baseline.

[5] Rate difference=(NVSI-06-09)-(BBIBP-CorV). Rate difference and 95%CI were estimated by CMH method considering stratification factors.

**Table S31:** **Comparison of the neutralizing antibody response against Omicron (BA.4) variant elicited by NVSI-06-09 booster between male and female subjects (PPS)**

|  | **NVSI-06-09** | | |
| --- | --- | --- | --- |
|  | **Male** | **Female** | ***p* value** |
| **All subjects** |  |  |  |
| N(missing) | 239(1) ^[6]^ | 16(0) |  |
| Pre-booster antibody titer ≥4, n (%) | 233(97.90) | 15(93.75) | 0.3259 |
| 95%CI (%) | 95.17-99.31 | 69.77-99.84 |  |
| Pre-booster antibody GMT^[1]^ (95%CI) | 42.63(37.51-48.45) | 19.60(10.42-36.85) | 0.0033 |
| Post-booster antibody GMT (95%CI) | 908.06(773.97-1065.38) | 942.49(462.22-1921.77) | 0.9088 |
| Ratio of GMT between two groups(95%CI)^[2]^ | 0.96(0.51-1.83) |  |  |
| Post-booster adjusted antibody GMT (95%CI)^[3]^ | 955.83(653.49-1398.06) | 1221.10(620.78-2401.96) | 0.4507 |
| Ratio of adjusted GMT between two groups(95%CI)^[3]^ | 0.78(0.41-1.48) |  |  |
| Rate of 4-fold rise^[4]^, n (%) | 219(92.02) | 16(100.0) | 0.2359 |
| 95%CI (%) | 87.81-95.13 | 79.41-100.00 |  |
| Rate difference between two groups (%, 95%CI^[5]^) | -8.22(-11.94--4.50) |  |  |
| Post-booster antibody GMT fold rise (95%CI) | 21.30(17.77-25.53) | 48.10(21.67-106.74) | 0.0276 |
| **All subjects (Excluding participants with positive**  **or weak positive COVID-19 PCR test)** |  |  |  |
| N(missing) | 216(1) ^[6]^ | 15(0) |  |
| Pre-booster antibody titer ≥4, n (%) | 212(98.60) | 15(100.00) | 1.0000 |
| 95%CI (%) | 95.98-99.71 | 78.20-100.00 |  |
| Pre-booster antibody GMT^[1]^ (95%CI) | 42.64(37.54-48.44) | 22.82(12.74-40.87) | 0.0149 |
| Post-booster antibody GMT (95%CI) | 910.75(768.70-1079.05) | 1047.95(506.62-2167.70) | 0.6782 |
| Ratio of GMT between two groups(95%CI)^[2]^ | 0.87(0.45-1.69) |  |  |
| Post-booster adjusted antibody GMT (95%CI)^[3]^ | 954.46(649.59-1402.44) | 1309.11(652.57-2626.16) | 0.3486 |
| Ratio of adjusted GMT between two groups(95%CI)^[3]^ | 0.73(0.38-1.41) |  |  |
| Rate of 4-fold rise^[4]^, n (%) | 200(93.02) | 15(100.0) | 0.2813 |
| 95%CI (%) | 88.75-96.04 | 78.20-100.00 |  |
| Rate difference between two groups (%, 95%CI^[5]^) | -7.31(-11.01--3.60) |  |  |
| Post-booster antibody GMT fold rise (95%CI) | 21.36(17.71-25.75) | 45.93(19.61-107.57) | 0.0420 |

Notes: [1] GMT represent geometric mean titer.

[2] The ratio of GMT between two groups was calculated by “NVSI-06-09/ BBIBP-CorV”.

[3] Covariance analysis with least square method was used to calculate the adjusted GMT and the corresponding *p* value.

[4] Rate of 4-fold rise was defined as percentage of participants with a ≥4-fold rise in neutralizing antibody titer from baseline.

[5] Rate difference=(NVSI-06-09)-(BBIBP-CorV). Rate difference and 95%CI were estimated by CMH method considering stratification factors.

[6] The serum sample of one subject was not tested due to contamination.

**Table S32: Comparison of the neutralizing antibody response against Omicron (BA.4) variant elicited by BBIBP-CorV booster between male and female subjects (PPS)**

|  | **BBIBP-CorV** | | |
| --- | --- | --- | --- |
|  | **Male** | **Female** | ***p* value** |
| **All subjects** |  |  |  |
| N(missing) | 237(0) | 12(0) |  |
| Pre-booster antibody titer ≥4, n (%) | 233(98.31) | 11(91.67) | 0.2204 |
| 95%CI (%) | 95.74-99.54 | 61.52-99.79 |  |
| Pre-booster antibody GMT^[1]^ (95%CI) | 42.15(36.70-48.41) | 41.93(19.50-90.18) | 0.9873 |
| Post-booster antibody GMT (95%CI) | 111.94(97.00-129.18) | 149.43(58.92-378.97) | 0.3914 |
| Ratio of GMT between two groups(95%CI)^[2]^ | 0.75(0.39-1.45) |  |  |
| Post-booster adjusted antibody GMT (95%CI)^[3]^ | 114.60(84.35-155.70) | 153.77(84.14-281.01) | 0.2781 |
| Ratio of adjusted GMT between two groups(95%CI)^[3]^ | 0.75(0.44-1.27) |  |  |
| Rate of 4-fold rise^[4]^, n (%) | 99(41.77) | 8(66.67) | 0.0888 |
| 95%CI (%) | 35.42-48.33 | 34.89-90.08 |  |
| Rate difference between two groups (%, 95%CI^[5]^) | -25.00(-52.43-2.43) |  |  |
| Post-booster antibody GMT fold rise (95%CI) | 2.66(2.34-3.01) | 3.56(1.67-7.60) | 0.3194 |
| **All subjects (Excluding participants with positive**  **or weak positive COVID-19 PCR test)** |  |  |  |
| N(missing) | 216(0) | 10(0) |  |
| Pre-booster antibody titer ≥4, n (%) | 212(98.15) | 9(90.00) | 0.2042 |
| 95%CI (%) | 95.33-99.49 | 55.50-99.75 |  |
| Pre-booster antibody GMT^[1]^ (95%CI) | 41.55(35.97-48.00) | 48.82(19.69-121.07) | 0.6462 |
| Post-booster antibody GMT (95%CI) | 112.85(96.96-131.33) | 195.29(69.41-549.46) | 0.1402 |
| Ratio of GMT between two groups(95%CI)^[2]^ | 0.58(0.28-1.20) |  |  |
| Post-booster adjusted antibody GMT (95%CI)^[3]^ | 110.03(77.58-156.05) | 171.59(87.53-336.38) | 0.1380 |
| Ratio of adjusted GMT between two groups(95%CI)^[3]^ | 0.64(0.36-1.15) |  |  |
| Rate of 4-fold rise^[4]^, n (%) | 90(41.67) | 7(70.00) | 0.0773 |
| 95%CI (%) | 35.02-48.55 | 34.75-93.33 |  |
| Rate difference between two groups (%, 95%CI^[5]^) | -28.37(-57.55-0.81) |  |  |
| Post-booster antibody GMT fold rise (95%CI) | 2.72(2.38-3.10) | 4.00(1.65-9.72) | 0.2328 |

Notes: [1] GMT represent geometric mean titer.

[2] The ratio of GMT between two groups was calculated by “NVSI-06-09/ BBIBP-CorV”.

[3] Covariance analysis with least square method was used to calculate the adjusted GMT and the corresponding *p* value.

[4] Rate of 4-fold rise was defined as percentage of participants with a ≥4-fold rise in neutralizing antibody titer from baseline.

[5] Rate difference=(NVSI-06-09)-(BBIBP-CorV). Rate difference and 95%CI were estimated by CMH method considering stratification factors.

**Table S33: Comparison of the neutralizing antibody response against Omicron (BA.5) variant elicited by NVSI-06-09 booster between male and female subjects (PPS)**

|  | **NVSI-06-09** | | |
| --- | --- | --- | --- |
|  | **Male** | **Female** | ***p* value** |
| **All subjects** |  |  |  |
| N(missing) | 239(1) ^[6]^ | 16(0) |  |
| Pre-booster antibody titer ≥4, n (%) | 233(97.90) | 15(93.75) | 0.3259 |
| 95%CI (%) | 95.17-99.31 | 69.77-99.84 |  |
| Pre-booster antibody GMT^[1]^ (95%CI) | 38.05(33.24-43.55) | 23.90(12.96-44.09) | 0.0920 |
| Post-booster antibody GMT (95%CI) | 1010.89(879.32-1162.13) | 849.21(426.81-1689.66) | 0.5419 |
| Ratio of GMT between two groups(95%CI)^[2]^ | 1.19(0.68-2.09) |  |  |
| Post-booster adjusted antibody GMT (95%CI)^[3]^ | 996.68(716.71-1386.00) | 959.93(536.61-1717.17) | 0.8925 |
| Ratio of adjusted GMT between two groups(95%CI)^[3]^ | 1.04(0.60-1.79) |  |  |
| Rate of 4-fold rise^[4]^, n (%) | 226(94.96) | 15(93.75) | 0.8851 |
| 95%CI (%) | 91.36-97.37 | 69.77-99.84 |  |
| Rate difference between two groups (%, 95%CI^[5]^) | 0.86(-11.55-13.26) |  |  |
| Post-booster antibody GMT fold rise (95%CI) | 26.57(22.53-31.34) | 35.53(16.55-76.29) | 0.3879 |
| **All subjects (Excluding participants with positive**  **or weak positive COVID-19 PCR test)** |  |  |  |
| N(missing) | 216(1) ^[6]^ | 15(0) |  |
| Pre-booster antibody titer ≥4, n (%) | 211(98.14) | 15(100.00) | 1.0000 |
| 95%CI (%) | 95.31-99.49 | 78.20-100.00 |  |
| Pre-booster antibody GMT^[1]^ (95%CI) | 38.36(33.34-44.13) | 28.20(16.46-48.32) | 0.2686 |
| Post-booster antibody GMT (95%CI) | 1001.84(863.34-1162.57) | 963.39(487.48-1903.92) | 0.8955 |
| Ratio of GMT between two groups(95%CI)^[2]^ | 1.04(0.58-1.87) |  |  |
| Post-booster adjusted antibody GMT (95%CI)^[3]^ | 989.52(708.43-1382.14) | 1046.60(574.84-1905.50) | 0.8463 |
| Ratio of adjusted GMT between two groups(95%CI)^[3]^ | 0.95(0.54-1.67) |  |  |
| Rate of 4-fold rise^[4]^, n (%) | 204(94.88) | 14(93.33) | 0.8472 |
| 95%CI (%) | 91.03-97.42 | 68.05-99.83 |  |
| Rate difference between two groups (%, 95%CI^[5]^) | 1.19(-12.02-14.40) |  |  |
| Post-booster antibody GMT fold rise (95%CI) | 26.12(22.00-31.00) | 34.16(15.09-77.34) | 0.4362 |

Notes: [1] GMT represent geometric mean titer.

[2] The ratio of GMT between two groups was calculated by “NVSI-06-09/ BBIBP-CorV”.

[3] Covariance analysis with least square method was used to calculate the adjusted GMT and the corresponding *p* value.

[4] Rate of 4-fold rise was defined as percentage of participants with a ≥4-fold rise in neutralizing antibody titer from baseline.

[5] Rate difference=(NVSI-06-09)-(BBIBP-CorV). Rate difference and 95%CI were estimated by CMH method considering stratification factors.

[6] The serum sample of one subject was not tested due to contamination.

**Table S34: Comparison of the neutralizing antibody response against Omicron (BA.5) variant elicited by BBIBP-CorV booster between male and female subjects (PPS)**

|  | **BBIBP-CorV** | | |
| --- | --- | --- | --- |
|  | **Male** | **Female** | ***p* value** |
| **All subjects** |  |  |  |
| N(missing) | 237(0) | 12(0) |  |
| Pre-booster antibody titer ≥4, n (%) | 227(95.78) | 11(91.67) | 0.4258 |
| 95%CI (%) | 92.38-97.96 | 61.52-99.79 |  |
| Pre-booster antibody GMT^[1]^ (95%CI) | 35.28(30.66-40.59) | 29.65(15.59-56.38) | 0.5913 |
| Post-booster antibody GMT (95%CI) | 164.59(141.95-190.84) | 182.01(75.08-441.23) | 0.7712 |
| Ratio of GMT between two groups(95%CI)^[2]^ | 0.90(0.46-1.79) |  |  |
| Post-booster adjusted antibody GMT (95%CI)^[3]^ | 131.08(93.05-184.64) | 156.07(79.66-305.78) | 0.5638 |
| Ratio of adjusted GMT between two groups(95%CI)^[3]^ | 0.84(0.46-1.52) |  |  |
| Rate of 4-fold rise^[4]^, n (%) | 140(59.07) | 7(58.33) | 0.9040 |
| 95%CI (%) | 52.52-65.39 | 27.67-84.83 |  |
| Rate difference between two groups (%, 95%CI^[5]^) | 1.75(-26.85-30.36) |  |  |
| Post-booster antibody GMT fold rise (95%CI) | 4.67(4.03-5.40) | 6.14(2.80-13.46) | 0.4186 |
| **All subjects (Excluding participants with positive**  **or weak positive COVID-19 PCR test)** |  |  |  |
| N(missing) | 216(0) | 10(0) |  |
| Pre-booster antibody titer ≥4, n (%) | 206(95.37) | 9(90.00) | 0.3992 |
| 95%CI (%) | 91.65-97.76 | 55.50-99.75 |  |
| Pre-booster antibody GMT^[1]^ (95%CI) | 34.96(30.13-40.57) | 30.05(13.53-66.76) | 0.6739 |
| Post-booster antibody GMT (95%CI) | 163.98(140.04-192.01) | 230.88(84.11-633.72) | 0.3738 |
| Ratio of GMT between two groups(95%CI)^[2]^ | 0.71(0.33-1.51) |  |  |
| Post-booster adjusted antibody GMT (95%CI)^[3]^ | 124.67(84.38-184.19) | 187.04(88.43-395.59) | 0.2238 |
| Ratio of adjusted GMT between two groups(95%CI)^[3]^ | 0.67(0.35-1.28) |  |  |
| Rate of 4-fold rise^[4]^, n (%) | 129(59.72) | 7(70.00) | 0.5399 |
| 95%CI (%) | 52.85-66.32 | 34.75-93.33 |  |
| Rate difference between two groups (%, 95%CI^[5]^) | -9.71(-38.88-19.45) |  |  |
| Post-booster antibody GMT fold rise (95%CI) | 4.69(4.02-5.47) | 7.68(3.19-18.51) | 0.1858 |

Notes: [1] GMT represent geometric mean titer.

[2] The ratio of GMT between two groups was calculated by “NVSI-06-09/ BBIBP-CorV”.

[3] Covariance analysis with least square method was used to calculate the adjusted GMT and the corresponding *p* value.

[4] Rate of 4-fold rise was defined as percentage of participants with a ≥4-fold rise in neutralizing antibody titer from baseline.

[5] Rate difference=(NVSI-06-09)-(BBIBP-CorV). Rate difference and 95%CI were estimated by CMH method considering stratification factors.

**Table S35: Comparison of the neutralizing antibody response against Omicron (BA.2) variant elicited by NVSI-06-09 booster between different age subgroups (PPS)**

|  | **NVSI-06-09** | | |
| --- | --- | --- | --- |
|  | **<45 year** | **≥45 year** | ***p* value** |
| **All subjects** |  |  |  |
| N(missing) | 199(1) ^[6]^ | 56(0) |  |
| Pre-booster antibody titer ≥4, n (%) | 192(96.97) | 54(96.43) | 1.0000 |
| 95%CI (%) | 93.52-98.88 | 87.69-99.56 |  |
| Pre-booster antibody GMT^[1]^ (95%CI) | 40.25(34.81-46.53) | 35.69(25.49-49.97) | 0.4657 |
| Post-booster antibody GMT (95%CI) | 943.74(799.53-1113.96) | 1137.24(855.87-1511.10) | 0.2882 |
| Ratio of GMT between two groups(95%CI)^[2]^ | 0.83(0.59-1.17) |  |  |
| Post-booster adjusted antibody GMT (95%CI)^[3]^ | 846.79(597.97-1199.14) | 1051.64(681.17-1623.58) | 0.2042 |
| Ratio of adjusted GMT between two groups(95%CI)^[3]^ | 0.81(0.58-1.13) |  |  |
| Rate of 4-fold rise^[4]^, n (%) | 187(94.44) | 53(94.64) | 0.9415 |
| 95%CI (%) | 90.28-97.19 | 85.13-98.88 |  |
| Rate difference between two groups (%, 95%CI^[5]^) | -0.25(-6.95-6.45) |  |  |
| Post-booster antibody GMT fold rise (95%CI) | 23.45(19.43-28.30) | 31.87(21.48-47.26) | 0.1407 |
| **All subjects (Excluding participants with positive**  **or weak positive COVID-19 PCR test)** |  |  |  |
| N(missing) | 180(1) ^[6]^ | 51 |  |
| Pre-booster antibody titer ≥4, n (%) | 174(97.21) | 50(98.04) | 1.0000 |
| 95%CI (%) | 93.60-99.09 | 89.55-99.95 |  |
| Pre-booster antibody GMT^[1]^ (95%CI) | 41.30(35.55-47.98) | 37.19(26.39-52.40) | 0.5352 |
| Post-booster antibody GMT (95%CI) | 922.63(773.57-1100.42) | 1243.74(933.40-1657.25) | 0.1058 |
| Ratio of GMT between two groups(95%CI)^[2]^ | 0.74(0.52-1.07) |  |  |
| Post-booster adjusted antibody GMT (95%CI)^[3]^ | 832.65(586.20-1182.70) | 1151.46(738.03-1796.48) | 0.0716 |
| Ratio of adjusted GMT between two groups(95%CI)^[3]^ | 0.72(0.51-1.03) |  |  |
| Rate of 4-fold rise^[4]^, n (%) | 168(93.85) | 49(96.08) | 0.5339 |
| 95%CI (%) | 89.27-96.89 | 86.54-99.52 |  |
| Rate difference between two groups (%, 95%CI^[5]^) | -2.29(-8.67-4.09) |  |  |
| Post-booster antibody GMT fold rise (95%CI) | 22.34(18.35-27.20) | 33.44(22.12-50.56) | 0.0640 |

Notes: [1] GMT represent geometric mean titer.

[2] The ratio of GMT between two groups was calculated by “NVSI-06-09/ BBIBP-CorV”.

[3] Covariance analysis with least square method was used to calculate the adjusted GMT and the corresponding *p* value.

[4] Rate of 4-fold rise was defined as percentage of participants with a ≥4-fold rise in neutralizing antibody titer from baseline.

[5] Rate difference=(NVSI-06-09)-(BBIBP-CorV). Rate difference and 95%CI were estimated by CMH method considering stratification factors.

[6] The serum sample of one subject was not tested due to contamination.

**Table S36: Comparison of the neutralizing antibody response against Omicron (BA.2) variant elicited by BBIBP-CorV booster between different age subgroups (PPS)**

|  | **BBIBP-CorV** | | |
| --- | --- | --- | --- |
|  | **<45 year** | **≥45 year** | ***p* value** |
| **All subjects** |  |  |  |
| N(missing) | 198(0) | 51(0) |  |
| Pre-booster antibody titer ≥4, n (%) | 193(97.47) | 48(94.12) | 0.2112 |
| 95%CI (%) | 94.21-99.18 | 83.76-98.77 |  |
| Pre-booster antibody GMT^[1]^ (95%CI) | 39.49(33.76-46.18) | 43.88(31.55-61.02) | 0.5525 |
| Post-booster antibody GMT (95%CI) | 114.08(98.29-132.40) | 130.86(100.58-170.25) | 0.4008 |
| Ratio of GMT between two groups(95%CI)^[2]^ | 0.87(0.63-1.20) |  |  |
| Post-booster adjusted antibody GMT (95%CI)^[3]^ | 106.53(81.51-139.24) | 115.04(84.00-157.57) | 0.5283 |
| Ratio of adjusted GMT between two groups(95%CI)^[3]^ | 0.93(0.73-1.18) |  |  |
| Rate of 4-fold rise^[4]^, n (%) | 81(40.91) | 23(45.10) | 0.5371 |
| 95%CI (%) | 33.99-48.10 | 31.13-59.66 |  |
| Rate difference between two groups (%, 95%CI^[5]^) | -4.79(-20.04-10.47) |  |  |
| Post-booster antibody GMT fold rise (95%CI) | 2.89(2.57-3.24) | 2.98(2.20-4.04) | 0.8194 |
| **All subjects (Excluding participants with positive**  **or weak positive COVID-19 PCR test)** |  |  |  |
| N(missing) | 180(0) | 46(0) |  |
| Pre-booster antibody titer ≥4, n (%) | 175(97.22) | 43(93.48) | 0.2080 |
| 95%CI (%) | 93.64-99.09 | 82.10-98.63 |  |
| Pre-booster antibody GMT^[1]^ (95%CI) | 40.04(34.02-47.13) | 42.54(29.80-60.72) | 0.7454 |
| Post-booster antibody GMT (95%CI) | 116.10(99.44-135.55) | 128.88(97.34-170.64) | 0.5410 |
| Ratio of GMT between two groups(95%CI)^[2]^ | 0.90(0.64-1.26) |  |  |
| Post-booster adjusted antibody GMT (95%CI)^[3]^ | 103.90(76.33-141.42) | 111.73(77.93-160.20) | 0.5812 |
| Ratio of adjusted GMT between two groups(95%CI)^[3]^ | 0.93(0.72-1.21) |  |  |
| Rate of 4-fold rise^[4]^, n (%) | 76(42.22) | 22(47.83) | 0.4763 |
| 95%CI (%) | 34.91-49.79 | 32.89-63.05 |  |
| Rate difference between two groups (%, 95%CI^[5]^) | -5.85(-22.00-10.30) |  |  |
| Post-booster antibody GMT fold rise (95%CI) | 2.90(2.56-3.29) | 3.03(2.16-4.24) | 0.7732 |

Notes: [1] GMT represent geometric mean titer.

[2] The ratio of GMT between two groups was calculated by “NVSI-06-09/ BBIBP-CorV”.

[3] Covariance analysis with least square method was used to calculate the adjusted GMT and the corresponding *p* value.

[4] Rate of 4-fold rise was defined as percentage of participants with a ≥4-fold rise in neutralizing antibody titer from baseline.

[5] Rate difference=(NVSI-06-09)-(BBIBP-CorV). Rate difference and 95%CI were estimated by CMH method considering stratification factors.

**Table S37: Comparison of the neutralizing antibody response against Omicron (BA.2) variant elicited by NVSI-06-09 booster between different age subgroups (PPS)**

|  | **NVSI-06-09** | | |
| --- | --- | --- | --- |
|  | **45-59 year** | **≥60 year** | ***p* value** |
| **All subjects** |  |  |  |
| N(missing) | 52(0) | 4(0) |  |
| Pre-booster antibody titer ≥4, n (%) | 50(96.15) | 4(100.00) | 1.0000 |
| 95%CI (%) | 86.79-99.53 | 39.76-100.00 |  |
| Pre-booster antibody GMT^[1]^ (95%CI) | 35.35(24.91-50.17) | 40.36(4.15-392.17) | 0.8410 |
| Post-booster antibody GMT (95%CI) | 1096.53(819.45-1467.30) | 1826.62(226.41-14736.43) | 0.3589 |
| Ratio of GMT between two groups(95%CI)^[2]^ | 0.60(0.20-1.81) |  |  |
| Post-booster adjusted antibody GMT (95%CI)^[3]^ | 1308.02(599.80-2852.49) | 2159.05(579.25-8047.55) | 0.3666 |
| Ratio of adjusted GMT between two groups(95%CI)^[3]^ | 0.61(0.20-1.83) |  |  |
| Rate of 4-fold rise^[4]^, n (%) | 49(94.23) | 4(100.0) | 0.6175 |
| 95%CI (%) | 84.05-98.79 | 39.76-100.00 |  |
| Rate difference between two groups (%, 95%CI^[5]^) | -6.00(-12.58-0.58) |  |  |
| Post-booster antibody GMT fold rise (95%CI) | 31.02(20.66-46.56) | 45.25(2.45-837.52) | 0.6253 |
| **All subjects (Excluding participants with positive**  **or weak positive COVID-19 PCR test)** |  |  |  |
| N(missing) | 47(0) | 4(0) |  |
| Pre-booster antibody titer ≥4, n (%) | 46(97.87) | 4(100.00) | 1.0000 |
| 95%CI (%) | 88.71-99.95 | 39.76-100.00 |  |
| Pre-booster antibody GMT^[1]^ (95%CI) | 36.93(25.83-52.80) | 40.36(4.15-392.17) | 0.8904 |
| Post-booster antibody GMT (95%CI) | 1203.71(896.67-1615.89) | 1826.62(226.41-14736.43) | 0.4382 |
| Ratio of GMT between two groups(95%CI)^[2]^ | 0.66(0.23-1.93) |  |  |
| Post-booster adjusted antibody GMT (95%CI)^[3]^ | 1334.12(618.90-2875.90) | 2020.36(555.05-7354.07) | 0.4456 |
| Ratio of adjusted GMT between two groups(95%CI)^[3]^ | 0.66(0.22-1.95) |  |  |
| Rate of 4-fold rise^[4]^, n (%) | 45(95.74) | 4(100.0) | 0.6700 |
| 95%CI (%) | 85.46-99.48 | 39.76-100.00 |  |
| Rate difference between two groups (%, 95%CI^[5]^) | -4.44(-10.47-1.58) |  |  |
| Post-booster antibody GMT fold rise (95%CI) | 32.59(21.25-49.98) | 45.25(2.45-837.52) | 0.6725 |

Notes: [1] GMT represent geometric mean titer.

[2] The ratio of GMT between two groups was calculated by “NVSI-06-09/ BBIBP-CorV”.

[3] Covariance analysis with least square method was used to calculate the adjusted GMT and the corresponding *p* value.

[4] Rate of 4-fold rise was defined as percentage of participants with a ≥4-fold rise in neutralizing antibody titer from baseline.

[5] Rate difference=(NVSI-06-09)-(BBIBP-CorV). Rate difference and 95%CI were estimated by CMH method considering stratification factors.

**Table S38: Comparison of the neutralizing antibody response against Omicron (BA.2) variant elicited by BBIBP-CorV booster between different age subgroups (PPS)**

|  | **BBIBP-CorV** | | |
| --- | --- | --- | --- |
|  | **45-59 year** | **≥60 year** | ***p* value** |
| **All subjects** |  |  |  |
| N(missing) | 50(0) | 1(0) |  |
| Pre-booster antibody titer ≥4, n (%) | 47(94.00) | 1(100.00) | 1.0000 |
| 95%CI (%) | 83.45-98.75 | 2.50-100.00 |  |
| Pre-booster antibody GMT^[1]^ (95%CI) | 43.20(30.90-60.40) | 96.00(NA) | 0.5057 |
| Post-booster antibody GMT (95%CI) | 127.33(97.91-165.60) | 512.00(NA) | 0.1426 |
| Ratio of GMT between two groups(95%CI)^[2]^ | 0.25(0.04-1.62) |  |  |
| Post-booster adjusted antibody GMT (95%CI)^[3]^ | 144.61(88.04-237.55) | 439.03(76.92-2505.76) | 0.1902 |
| Ratio of adjusted GMT between two groups(95%CI)^[3]^ | 0.33(0.06-1.77) |  |  |
| Rate of 4-fold rise^[4]^, n (%) | 22(44.00) | 1(100.0) | 0.2770 |
| 95%CI (%) | 29.99-58.75 | 2.50-100.00 |  |
| Rate difference between two groups (%, 95%CI^[5]^) | -55.32(-69.53--41.11) |  |  |
| Post-booster antibody GMT fold rise (95%CI) | 2.95(2.16-4.02) | 5.33(NA) | 0.5924 |
| **All subjects (Excluding participants with positive**  **or weak positive COVID-19 PCR test)** |  |  |  |
| N(missing) | 45(0) | 1(0) |  |
| Pre-booster antibody titer ≥4, n (%) | 42(93.33) | 1(100.00) | 1.0000 |
| 95%CI (%) | 81.73-98.60 | 2.50-100.00 |  |
| Pre-booster antibody GMT^[1]^ (95%CI) | 41.77(29.08-60.01) | 96.00(NA) | 0.4985 |
| Post-booster antibody GMT (95%CI) | 124.99(94.45-165.39) | 512.00(NA) | 0.1418 |
| Ratio of GMT between two groups(95%CI)^[2]^ | 0.24(0.04-1.63) |  |  |
| Post-booster adjusted antibody GMT (95%CI)^[3]^ | 133.67(71.85-248.69) | 411.75(65.69-2580.80) | 0.2002 |
| Ratio of adjusted GMT between two groups(95%CI)^[3]^ | 0.32(0.06-1.86) |  |  |
| Rate of 4-fold rise^[4]^, n (%) | 21(46.67) | 1(100.0) | 0.2953 |
| 95%CI (%) | 31.66-62.13 | 2.50-100.00 |  |
| Rate difference between two groups (%, 95%CI^[5]^) | -53.49(-68.40--38.58) |  |  |
| Post-booster antibody GMT fold rise (95%CI) | 2.99(2.12-4.22) | 5.33(NA) | 0.6189 |

Notes: [1] GMT represent geometric mean titer.

[2] The ratio of GMT between two groups was calculated by “NVSI-06-09/ BBIBP-CorV”.

[3] Covariance analysis with least square method was used to calculate the adjusted GMT and the corresponding *p* value.

[4] Rate of 4-fold rise was defined as percentage of participants with a ≥4-fold rise in neutralizing antibody titer from baseline.

[5] Rate difference=(NVSI-06-09)-(BBIBP-CorV). Rate difference and 95%CI were estimated by CMH method considering stratification factors.

**Table S39:** **Comparison of the neutralizing antibody response against Omicron (BA.4) variant elicited by NVSI-06-09 booster between different age subgroups (PPS)**

|  | **NVSI-06-09** | | |
| --- | --- | --- | --- |
|  | **<45 year** | **≥45 year** | ***p* value** |
| **All subjects** |  |  |  |
| N(missing) | 199(1) ^[6]^ | 56(0) |  |
| Pre-booster antibody titer ≥4, n (%) | 194(97.98) | 54(96.43) | 0.6159 |
| 95%CI (%) | 94.91-99.45 | 87.69-99.56 |  |
| Pre-booster antibody GMT^[1]^ (95%CI) | 42.34(36.77-48.75) | 35.00(26.04-47.03) | 0.2223 |
| Post-booster antibody GMT (95%CI) | 856.67(718.27-1021.72) | 1127.71(811.27-1567.58) | 0.1479 |
| Ratio of GMT between two groups(95%CI)^[2]^ | 0.76(0.52-1.10) |  |  |
| Post-booster adjusted antibody GMT (95%CI)^[3]^ | 920.68(630.81-1343.77) | 1281.37(798.89-2055.26) | 0.0759 |
| Ratio of adjusted GMT between two groups(95%CI)^[3]^ | 0.72(0.50-1.04) |  |  |
| Rate of 4-fold rise^[4]^, n (%) | 183(92.42) | 52(92.86) | 0.9170 |
| 95%CI (%) | 87.81-95.70 | 82.71-98.02 |  |
| Rate difference between two groups (%, 95%CI^[5]^) | -0.42(-8.10-7.27) |  |  |
| Post-booster antibody GMT fold rise (95%CI) | 20.24(16.61-24.65) | 32.22(21.69-47.87) | 0.0318 |
| **All subjects (Excluding participants with positive**  **or weak positive COVID-19 PCR test)** |  |  |  |
| N(missing) | 180(1) ^[6]^ | 51(0) |  |
| Pre-booster antibody titer ≥4, n (%) | 177(98.88) | 50(98.04) | 0.5304 |
| 95%CI (%) | 96.02-99.86 | 89.55-99.95 |  |
| Pre-booster antibody GMT^[1]^ (95%CI) | 42.40(36.90-48.73) | 36.19(27.02-48.49) | 0.3022 |
| Post-booster antibody GMT (95%CI) | 853.40(707.87-1028.85) | 1192.51(846.11-1680.72) | 0.0950 |
| Ratio of GMT between two groups(95%CI)^[2]^ | 0.72(0.48-1.06) |  |  |
| Post-booster adjusted antibody GMT (95%CI)^[3]^ | 917.18(625.76-1344.31) | 1346.40(828.92-2186.94) | 0.0509 |
| Ratio of adjusted GMT between two groups(95%CI)^[3]^ | 0.68(0.46-1.00) |  |  |
| Rate of 4-fold rise^[4]^, n (%) | 167(93.30) | 48(94.12) | 0.8404 |
| 95%CI (%) | 88.58-96.49 | 83.76-98.77 |  |
| Rate difference between two groups (%, 95%CI^[5]^) | -0.79(-8.21-6.63) |  |  |
| Post-booster antibody GMT fold rise (95%CI) | 20.13(16.37-24.74) | 32.95(22.20-48.89) | 0.0275 |

Notes: [1] GMT represent geometric mean titer.

[2] The ratio of GMT between two groups was calculated by “NVSI-06-09/ BBIBP-CorV”.

[3] Covariance analysis with least square method was used to calculate the adjusted GMT and the corresponding *p* value.

[4] Rate of 4-fold rise was defined as percentage of participants with a ≥4-fold rise in neutralizing antibody titer from baseline.

[5] Rate difference=(NVSI-06-09)-(BBIBP-CorV). Rate difference and 95%CI were estimated by CMH method considering stratification factors.

[6] The serum sample of one subject was not tested due to contamination.

**Table S40: Comparison of the neutralizing antibody response against Omicron (BA.4) variant elicited by BBIBP-CorV booster between different age subgroups (PPS)**

|  | **BBIBP-CorV** | | |
| --- | --- | --- | --- |
|  | **<45 year** | **≥45 year** | ***p* value** |
| **All subjects** |  |  |  |
| N(missing) | 198(0) | 51(0) |  |
| Pre-booster antibody titer ≥4, n (%) | 194(97.98) | 50(98.04) | 1.0000 |
| 95%CI (%) | 94.91-99.45 | 89.55-99.95 |  |
| Pre-booster antibody GMT^[1]^ (95%CI) | 42.39(36.37-49.41) | 41.18(30.52-55.55) | 0.8651 |
| Post-booster antibody GMT (95%CI) | 115.53(98.62-135.34) | 106.01(76.25-147.40) | 0.6310 |
| Ratio of GMT between two groups(95%CI)^[2]^ | 1.09(0.77-1.55) |  |  |
| Post-booster adjusted antibody GMT (95%CI)^[3]^ | 117.60(85.74-161.31) | 109.75(75.78-158.96) | 0.6319 |
| Ratio of adjusted GMT between two groups(95%CI)^[3]^ | 1.07(0.81-1.42) |  |  |
| Rate of 4-fold rise^[4]^, n (%) | 84(42.42) | 23(45.10) | 0.7359 |
| 95%CI (%) | 35.45-49.63 | 31.13-59.66 |  |
| Rate difference between two groups (%, 95%CI^[5]^) | -2.64(-17.99-12.71) |  |  |
| Post-booster antibody GMT fold rise (95%CI) | 2.73(2.37-3.14) | 2.57(1.96-3.38) | 0.7167 |
| **All subjects (Excluding participants with positive**  **or weak positive COVID-19 PCR test)** |  |  |  |
| N(missing) | 180(0) | 46(0) |  |
| Pre-booster antibody titer ≥4, n (%) | 176(97.78) | 45(97.83) | 1.0000 |
| 95%CI (%) | 94.41-99.39 | 88.47-99.94 |  |
| Pre-booster antibody GMT^[1]^ (95%CI) | 42.43(36.16-49.80) | 39.64(28.84-54.49) | 0.7042 |
| Post-booster antibody GMT (95%CI) | 116.64(98.52-138.10) | 111.71(79.10-157.76) | 0.8204 |
| Ratio of GMT between two groups(95%CI)^[2]^ | 1.04(0.72-1.52) |  |  |
| Post-booster adjusted antibody GMT (95%CI)^[3]^ | 111.06(77.59-158.97) | 111.22(73.19-169.01) | 0.9927 |
| Ratio of adjusted GMT between two groups(95%CI)^[3]^ | 1.00(0.74-1.35) |  |  |
| Rate of 4-fold rise^[4]^, n (%) | 76(42.22) | 21(45.65) | 0.6763 |
| 95%CI (%) | 34.91-49.79 | 30.90-60.99 |  |
| Rate difference between two groups (%, 95%CI^[5]^) | -3.44(-19.55-12.68) |  |  |
| Post-booster antibody GMT fold rise (95%CI) | 2.75(2.36-3.20) | 2.82(2.16-3.68) | 0.8808 |

Notes: [1] GMT represent geometric mean titer.

[2] The ratio of GMT between two groups was calculated by “NVSI-06-09/ BBIBP-CorV”.

[3] Covariance analysis with least square method was used to calculate the adjusted GMT and the corresponding *p* value.

[4] Rate of 4-fold rise was defined as percentage of participants with a ≥4-fold rise in neutralizing antibody titer from baseline.

[5] Rate difference=(NVSI-06-09)-(BBIBP-CorV). Rate difference and 95%CI were estimated by CMH method considering stratification factors.

**Table S41: Comparison of the neutralizing antibody response against Omicron (BA.4) variant elicited by NVSI-06-09 booster between different age subgroups (PPS)**

|  | **NVSI-06-09** | | |
| --- | --- | --- | --- |
|  | **45-59 year** | **≥60 year** | ***p* value** |
| **All subjects** |  |  |  |
| N(missing) | 52(0) | 4(0) |  |
| Pre-booster antibody titer ≥4, n (%) | 50(96.15) | 4(100.00) | 1.0000 |
| 95%CI (%) | 86.79-99.53 | 39.76-100.00 |  |
| Pre-booster antibody GMT^[1]^ (95%CI) | 34.42(25.34-46.75) | 43.37(5.46-344.72) | 0.6903 |
| Post-booster antibody GMT (95%CI) | 1083.09(768.69-1526.10) | 1905.88(263.93-13762.72) | 0.3807 |
| Ratio of GMT between two groups(95%CI)^[2]^ | 0.57(0.16-2.05) |  |  |
| Post-booster adjusted antibody GMT (95%CI)^[3]^ | 2381.44(1006.27-5635.89) | 4232.98(974.45-18388.00) | 0.3561 |
| Ratio of adjusted GMT between two groups(95%CI)^[3]^ | 0.56(0.16-1.94) |  |  |
| Rate of 4-fold rise^[4]^, n (%) | 48(92.31) | 4(100.0) | 0.5603 |
| 95%CI (%) | 81.46-97.86 | 39.76-100.00 |  |
| Rate difference between two groups (%, 95%CI^[5]^) | -8.00(-15.52--0.48) |  |  |
| Post-booster antibody GMT fold rise (95%CI) | 31.47(20.89-47.40) | 43.94(2.70-714.93) | 0.6672 |
| **All subjects (Excluding participants with positive**  **or weak positive COVID-19 PCR test)** |  |  |  |
| N(missing) | 47(0) | 4(0) |  |
| Pre-booster antibody titer ≥4, n (%) | 46(97.87) | 4(100.00) | 1.0000 |
| 95%CI (%) | 88.71-99.95 | 39.76-100.00 |  |
| Pre-booster antibody GMT^[1]^ (95%CI) | 35.64(26.34-48.23) | 43.37(5.46-344.72) | 0.7208 |
| Post-booster antibody GMT (95%CI) | 1145.86(800.08-1641.08) | 1905.88(263.93-13762.72) | 0.4289 |
| Ratio of GMT between two groups(95%CI)^[2]^ | 0.60(0.17-2.17) |  |  |
| Post-booster adjusted antibody GMT (95%CI)^[3]^ | 2487.85(1061.01-5833.47) | 4191.00(983.60-17857.30) | 0.3969 |
| Ratio of adjusted GMT between two groups(95%CI)^[3]^ | 0.59(0.17-2.03) |  |  |
| Rate of 4-fold rise^[4]^, n (%) | 44(93.62) | 4(100.0) | 0.5978 |
| 95%CI (%) | 82.46-98.66 | 39.76-100.00 |  |
| Rate difference between two groups (%, 95%CI^[5]^) | -6.67(-13.95-0.62) |  |  |
| Post-booster antibody GMT fold rise (95%CI) | 32.15(21.38-48.35) | 43.94(2.70-714.93) | 0.6735 |

Notes: [1] GMT represent geometric mean titer.

[2] The ratio of GMT between two groups was calculated by “NVSI-06-09/ BBIBP-CorV”.

[3] Covariance analysis with least square method was used to calculate the adjusted GMT and the corresponding *p* value.

[4] Rate of 4-fold rise was defined as percentage of participants with a ≥4-fold rise in neutralizing antibody titer from baseline.

[5] Rate difference=(NVSI-06-09)-(BBIBP-CorV). Rate difference and 95%CI were estimated by CMH method considering stratification factors.

**Table S42: Comparison of the neutralizing antibody response against Omicron (BA.4) variant elicited by BBIBP-CorV booster between different age subgroups (PPS)**

|  | **BBIBP-CorV** | | |
| --- | --- | --- | --- |
|  | **45-59 year** | **≥60 year** | ***p* value** |
| **All subjects** |  |  |  |
| N(missing) | 50(0) | 1(0) |  |
| Pre-booster antibody titer ≥4, n (%) | 49(98.00) | 1(100.00) | 1.0000 |
| 95%CI (%) | 89.35-99.95 | 2.50-100.00 |  |
| Pre-booster antibody GMT^[1]^ (95%CI) | 40.48(29.88-54.85) | 96.00(NA) | 0.4276 |
| Post-booster antibody GMT (95%CI) | 103.32(74.11-144.04) | 384.00(NA) | 0.2716 |
| Ratio of GMT between two groups(95%CI)^[2]^ | 0.27(0.03-2.89) |  |  |
| Post-booster adjusted antibody GMT (95%CI)^[3]^ | 135.50(77.54-236.77) | 295.20(41.41-2104.48) | 0.4126 |
| Ratio of adjusted GMT between two groups(95%CI)^[3]^ | 0.46(0.07-3.05) |  |  |
| Rate of 4-fold rise^[4]^, n (%) | 22(44.00) | 1(100.0) | 0.2568 |
| 95%CI (%) | 29.99-58.75 | 2.50-100.00 |  |
| Rate difference between two groups (%, 95%CI^[5]^) | -57.45(-71.58--43.31) |  |  |
| Post-booster antibody GMT fold rise (95%CI) | 2.55(1.93-3.37) | 4.00(NA) | 0.6516 |
| **All subjects (Excluding participants with positive**  **or weak positive COVID-19 PCR test)** |  |  |  |
| N(missing) | 45(0) | 1(0) |  |
| Pre-booster antibody titer ≥4, n (%) | 44(97.78) | 1(100.00) | 1.0000 |
| 95%CI (%) | 88.23-99.94 | 2.50-100.00 |  |
| Pre-booster antibody GMT^[1]^ (95%CI) | 38.87(28.14-53.69) | 96.00(NA) | 0.4100 |
| Post-booster antibody GMT (95%CI) | 108.68(76.69-154.02) | 384.00(NA) | 0.2879 |
| Ratio of GMT between two groups(95%CI)^[2]^ | 0.28(0.03-3.01) |  |  |
| Post-booster adjusted antibody GMT (95%CI)^[3]^ | 130.48(68.80-247.45) | 244.32(36.67-1627.86) | 0.4854 |
| Ratio of adjusted GMT between two groups(95%CI)^[3]^ | 0.53(0.09-3.23) |  |  |
| Rate of 4-fold rise^[4]^, n (%) | 20(44.44) | 1(100.0) | 0.2733 |
| 95%CI (%) | 29.64-60.00 | 2.50-100.00 |  |
| Rate difference between two groups (%, 95%CI^[5]^) | -55.81(-70.66--40.97) |  |  |
| Post-booster antibody GMT fold rise (95%CI) | 2.80(2.13-3.67) | 4.00(NA) | 0.6977 |

Notes: [1] GMT represent geometric mean titer.

[2] The ratio of GMT between two groups was calculated by “NVSI-06-09/ BBIBP-CorV”.

[3] Covariance analysis with least square method was used to calculate the adjusted GMT and the corresponding *p* value.

[4] Rate of 4-fold rise was defined as percentage of participants with a ≥4-fold rise in neutralizing antibody titer from baseline.

[5] Rate difference=(NVSI-06-09)-(BBIBP-CorV). Rate difference and 95%CI were estimated by CMH method considering stratification factors.

**Table S43: Comparison of the neutralizing antibody response against Omicron (BA.5) variant elicited by NVSI-06-09 booster between different age subgroups (PPS)**

|  | **NVSI-06-09** | | |
| --- | --- | --- | --- |
|  | **<45 year** | **≥45 year** | ***p* value** |
| **All subjects** |  |  |  |
| N(missing) | 199(1) ^[6]^ | 56(0) |  |
| Pre-booster antibody titer ≥4, n (%) | 194(97.98) | 54(96.43) | 0.6159 |
| 95%CI (%) | 94.91-99.45 | 87.69-99.56 |  |
| Pre-booster antibody GMT^[1]^ (95%CI) | 38.18(33.01-44.17) | 32.89(24.06-44.98) | 0.3575 |
| Post-booster antibody GMT (95%CI) | 968.31(829.79-1129.94) | 1119.84(831.33-1508.49) | 0.3850 |
| Ratio of GMT between two groups(95%CI)^[2]^ | 0.86(0.62-1.20) |  |  |
| Post-booster adjusted antibody GMT (95%CI)^[3]^ | 955.57(687.85-1327.50) | 1155.06(766.11-1741.48) | 0.2400 |
| Ratio of adjusted GMT between two groups(95%CI)^[3]^ | 0.83(0.60-1.14) |  |  |
| Rate of 4-fold rise^[4]^, n (%) | 189(95.45) | 52(92.86) | 0.4300 |
| 95%CI (%) | 91.55-97.90 | 82.71-98.02 |  |
| Rate difference between two groups (%, 95%CI^[5]^) | 2.64(-4.70-9.98) |  |  |
| Post-booster antibody GMT fold rise (95%CI) | 25.36(21.29-30.21) | 34.04(23.03-50.31) | 0.1348 |
| **All subjects (Excluding participants with positive**  **or weak positive COVID-19 PCR test)** |  |  |  |
| N(missing) | 180(1) ^[6]^ | 51(1) |  |
| Pre-booster antibody titer ≥4, n (%) | 176(98.32) | 50(98.04) | 1.0000 |
| 95%CI (%) | 95.18-99.65 | 89.55-99.95 |  |
| Pre-booster antibody GMT^[1]^ (95%CI) | 38.54(33.19-44.75) | 34.47(25.05-47.43) | 0.5000 |
| Post-booster antibody GMT (95%CI) | 949.86(806.78-1118.31) | 1194.05(870.31-1638.20) | 0.1957 |
| Ratio of GMT between two groups(95%CI)^[2]^ | 0.80(0.56-1.13) |  |  |
| Post-booster adjusted antibody GMT (95%CI)^[3]^ | 944.94(677.71-1317.56) | 1229.66(806.85-1874.05) | 0.1221 |
| Ratio of adjusted GMT between two groups(95%CI)^[3]^ | 0.77(0.55-1.07) |  |  |
| Rate of 4-fold rise^[4]^, n (%) | 170(94.97) | 48(94.12) | 0.7993 |
| 95%CI (%) | 90.67-97.68 | 83.76-98.77 |  |
| Rate difference between two groups (%, 95%CI^[5]^) | 0.90(-6.31-8.11) |  |  |
| Post-booster antibody GMT fold rise (95%CI) | 24.65(20.55-29.56) | 34.64(23.06-52.03) | 0.0961 |

Notes: [1] GMT represent geometric mean titer.

[2] The ratio of GMT between two groups was calculated by “NVSI-06-09/ BBIBP-CorV”.

[3] Covariance analysis with least square method was used to calculate the adjusted GMT and the corresponding *p* value.

[4] Rate of 4-fold rise was defined as percentage of participants with a ≥4-fold rise in neutralizing antibody titer from baseline.

[5] Rate difference=(NVSI-06-09)-(BBIBP-CorV). Rate difference and 95%CI were estimated by CMH method considering stratification factors.

[6] The serum sample of one subject was not tested due to contamination.

**Table S44: Comparison of the neutralizing antibody response against Omicron (BA.5) variant elicited by BBIBP-CorV booster between different age subgroups (PPS)**

|  | **BBIBP-CorV** | | |
| --- | --- | --- | --- |
|  | **<45 year** | **≥45 year** | ***p* value** |
| **All subjects** |  |  |  |
| N(missing) | 198(0) | 51(0) |  |
| Pre-booster antibody titer ≥4, n (%) | 189(95.45) | 49(96.08) | 1.0000 |
| 95%CI (%) | 91.55-97.90 | 86.54-99.52 |  |
| Pre-booster antibody GMT^[1]^ (95%CI) | 34.22(29.35-39.90) | 38.13(28.15-51.64) | 0.5289 |
| Post-booster antibody GMT (95%CI) | 163.64(138.65-193.14) | 172.35(126.16-235.45) | 0.7777 |
| Ratio of GMT between two groups(95%CI)^[2]^ | 0.95(0.66-1.36) |  |  |
| Post-booster adjusted antibody GMT (95%CI)^[3]^ | 131.39(92.36-186.91) | 132.52(87.58-200.53) | 0.9574 |
| Ratio of adjusted GMT between two groups(95%CI)^[3]^ | 0.99(0.72-1.36) |  |  |
| Rate of 4-fold rise^[4]^, n (%) | 118(59.60) | 29(56.86) | 0.7978 |
| 95%CI (%) | 52.41-66.49 | 42.25-70.65 |  |
| Rate difference between two groups (%, 95%CI^[5]^) | 1.98(-13.16-17.12) |  |  |
| Post-booster antibody GMT fold rise (95%CI) | 4.78(4.06-5.64) | 4.52(3.40-6.02) | 0.7545 |
| **All subjects (Excluding participants with positive**  **or weak positive COVID-19 PCR test)** |  |  |  |
| N(missing) | 180(0) | 46(0) |  |
| Pre-booster antibody titer ≥4, n (%) | 171(95.00) | 44(95.65) | 1.0000 |
| 95%CI (%) | 90.72-97.69 | 85.16-99.47 |  |
| Pre-booster antibody GMT^[1]^ (95%CI) | 34.10(28.98-40.13) | 37.28(26.71-52.03) | 0.6277 |
| Post-booster antibody GMT (95%CI) | 163.24(136.84-194.74) | 179.79(128.04-252.45) | 0.6233 |
| Ratio of GMT between two groups(95%CI)^[2]^ | 0.91(0.62-1.34) |  |  |
| Post-booster adjusted antibody GMT (95%CI)^[3]^ | 124.16(83.27-185.11) | 131.54(82.46-209.83) | 0.7348 |
| Ratio of adjusted GMT between two groups(95%CI)^[3]^ | 0.94(0.67-1.32) |  |  |
| Rate of 4-fold rise^[4]^, n (%) | 108(60.00) | 28(60.87) | 0.8872 |
| 95%CI (%) | 52.45-67.22 | 45.37-74.91 |  |
| Rate difference between two groups (%, 95%CI^[5]^) | -1.15(-16.95-14.64) |  |  |
| Post-booster antibody GMT fold rise (95%CI) | 4.79(4.02-5.69) | 4.82(3.54-6.58) | 0.9684 |

Notes: [1] GMT represent geometric mean titer.

[2] The ratio of GMT between two groups was calculated by “NVSI-06-09/ BBIBP-CorV”.

[3] Covariance analysis with least square method was used to calculate the adjusted GMT and the corresponding *p* value.

[4] Rate of 4-fold rise was defined as percentage of participants with a ≥4-fold rise in neutralizing antibody titer from baseline.

[5] Rate difference=(NVSI-06-09)-(BBIBP-CorV). Rate difference and 95%CI were estimated by CMH method considering stratification factors.

**Table S45: Comparison of the neutralizing antibody response against Omicron (BA.5) variant elicited by NVSI-06-09 booster between different age subgroups (PPS)**

|  | **NVSI-06-09** | | |
| --- | --- | --- | --- |
|  | **45-59 year** | **≥60 year** | ***p* value** |
| **All subjects** |  |  |  |
| N(missing) | 52(0) | 4(0) |  |
| Pre-booster antibody titer ≥4, n (%) | 50(96.15) | 4(100.00) | 1.0000 |
| 95%CI (%) | 86.79-99.53 | 39.76-100.00 |  |
| Pre-booster antibody GMT^[1]^ (95%CI) | 33.15(23.79-46.19) | 29.78(6.88-128.98) | 0.8615 |
| Post-booster antibody GMT (95%CI) | 1078.48(793.67-1465.49) | 1826.62(226.41-14736.43) | 0.3661 |
| Ratio of GMT between two groups(95%CI)^[2]^ | 0.59(0.19-1.88) |  |  |
| Post-booster adjusted antibody GMT (95%CI)^[3]^ | 2025.24(910.88-4502.89) | 3702.29(954.50-14360.43) | 0.2899 |
| Ratio of adjusted GMT between two groups(95%CI)^[3]^ | 0.55(0.18-1.70) |  |  |
| Rate of 4-fold rise^[4]^, n (%) | 48(92.31) | 4(100.0) | 0.5603 |
| 95%CI (%) | 81.46-97.86 | 39.76-100.00 |  |
| Rate difference between two groups (%, 95%CI^[5]^) | -8.00(-15.52--0.48) |  |  |
| Post-booster antibody GMT fold rise (95%CI) | 32.54(21.51-49.22) | 61.34(13.16-285.86) | 0.4072 |
| **All subjects (Excluding participants with positive**  **or weak positive COVID-19 PCR test)** |  |  |  |
| N(missing) | 47(0) | 4(0) |  |
| Pre-booster antibody titer ≥4, n (%) | 46(97.87) | 4(100.00) | 1.0000 |
| 95%CI (%) | 88.71-99.95 | 39.76-100.00 |  |
| Pre-booster antibody GMT^[1]^ (95%CI) | 34.90(24.84-49.05) | 29.78(6.88-128.98) | 0.7913 |
| Post-booster antibody GMT (95%CI) | 1151.62(829.91-1598.03) | 1826.62(226.41-14736.43) | 0.4364 |
| Ratio of GMT between two groups(95%CI)^[2]^ | 0.63(0.19-2.05) |  |  |
| Post-booster adjusted antibody GMT (95%CI)^[3]^ | 2116.78(933.78-4798.53) | 3689.42(922.23-14759.75) | 0.3394 |
| Ratio of adjusted GMT between two groups(95%CI)^[3]^ | 0.57(0.18-1.83) |  |  |
| Rate of 4-fold rise^[4]^, n (%) | 44(93.62) | 4(100.0) | 0.5978 |
| 95%CI (%) | 82.46-98.66 | 39.76-100.00 |  |
| Rate difference between two groups (%, 95%CI^[5]^) | -6.67(-13.95-0.62) |  |  |
| Post-booster antibody GMT fold rise (95%CI) | 32.99(21.38-50.92) | 61.34(13.16-285.86) | 0.4161 |

Notes: [1] GMT represent geometric mean titer.

[2] The ratio of GMT between two groups was calculated by “NVSI-06-09/ BBIBP-CorV”.

[3] Covariance analysis with least square method was used to calculate the adjusted GMT and the corresponding *p* value.

[4] Rate of 4-fold rise was defined as percentage of participants with a ≥4-fold rise in neutralizing antibody titer from baseline.

[5] Rate difference=(NVSI-06-09)-(BBIBP-CorV). Rate difference and 95%CI were estimated by CMH method considering stratification factors.

**Table S46: Comparison of the neutralizing antibody response against Omicron (BA.5) variant elicited by BBIBP-CorV booster between different age subgroups (PPS)**

|  | **BBIBP-CorV** | | |
| --- | --- | --- | --- |
|  | **45-59 year** | **≥60 year** | ***p* value** |
| **All subjects** |  |  |  |
| N(missing) | 50(0) | 1(0) |  |
| Pre-booster antibody titer ≥4, n (%) | 48(96.00) | 1(100.00) | 1.0000 |
| 95%CI (%) | 86.29-99.51 | 2.50-100.00 |  |
| Pre-booster antibody GMT^[1]^ (95%CI) | 37.43(27.53-50.89) | 96.00(NA) | 0.3926 |
| Post-booster antibody GMT (95%CI) | 168.64(123.03-231.15) | 512.00(NA) | 0.3265 |
| Ratio of GMT between two groups(95%CI)^[2]^ | 0.33(0.03-3.13) |  |  |
| Post-booster adjusted antibody GMT (95%CI)^[3]^ | 162.05(92.12-285.05) | 283.06(38.66-2072.67) | 0.5619 |
| Ratio of adjusted GMT between two groups(95%CI)^[3]^ | 0.57(0.08-3.91) |  |  |
| Rate of 4-fold rise^[4]^, n (%) | 28(56.00) | 1(100.0) | 0.3980 |
| 95%CI (%) | 41.25-70.01 | 2.50-100.00 |  |
| Rate difference between two groups (%, 95%CI^[5]^) | -42.55(-56.69--28.42) |  |  |
| Post-booster antibody GMT fold rise (95%CI) | 4.51(3.37-6.03) | 5.33(NA) | 0.8714 |
| **All subjects (Excluding participants with positive**  **or weak positive COVID-19 PCR test)** |  |  |  |
| N(missing) | 45(0) | 1(0) |  |
| Pre-booster antibody titer ≥4, n (%) | 43(95.56) | 1(100.00) | 1.0000 |
| 95%CI (%) | 84.85-99.46 | 2.50-100.00 |  |
| Pre-booster antibody GMT^[1]^ (95%CI) | 36.50(26.03-51.20) | 96.00(NA) | 0.4003 |
| Post-booster antibody GMT (95%CI) | 175.66(124.54-247.77) | 512.00(NA) | 0.3604 |
| Ratio of GMT between two groups(95%CI)^[2]^ | 0.34(0.03-3.54) |  |  |
| Post-booster adjusted antibody GMT (95%CI)^[3]^ | 167.37(82.49-339.60) | 276.79(34.02-2251.79) | 0.6119 |
| Ratio of adjusted GMT between two groups(95%CI)^[3]^ | 0.60(0.08-4.41) |  |  |
| Rate of 4-fold rise^[4]^, n (%) | 27(60.00) | 1(100.0) | 0.4275 |
| 95%CI (%) | 44.33-74.30 | 2.50-100.00 |  |
| Rate difference between two groups (%, 95%CI^[5]^) | -39.53(-54.15--24.92) |  |  |
| Post-booster antibody GMT fold rise (95%CI) | 4.81(3.50-6.61) | 5.33(NA) | 0.9237 |

Notes: [1] GMT represent geometric mean titer.

[2] The ratio of GMT between two groups was calculated by “NVSI-06-09/ BBIBP-CorV”.

[3] Covariance analysis with least square method was used to calculate the adjusted GMT and the corresponding *p* value.

[4] Rate of 4-fold rise was defined as percentage of participants with a ≥4-fold rise in neutralizing antibody titer from baseline.

[5] Rate difference=(NVSI-06-09)-(BBIBP-CorV). Rate difference and 95%CI were estimated by CMH method considering stratification factors.

**Table S47: Live-virus neutralizing antibody responses against Beta and Delta variants**

|  | **NVSI-06-09** | **BBIBP-CorV** | **GMT ratio (95%CI)** | ***p* value** |
| --- | --- | --- | --- | --- |
| **Beta strain** |  |  |  |  |
| n | 99 | 100 |  |  |
| GMT (95%CI) | 3075.59(2393.00-3952.89) | 465.69(376.75-575.63) | 6.60(4.77-9.15) | <0.0001 |
| **Delta strain** |  |  |  |  |
| n | 99 | 100 |  |  |
| GMT (95%CI) | 2831.50(2200.42-3643.58) | 395.05(316.82-492.58) | 7.17(5.14-10.00) | <0.0001 |
| **Prototype strain** |  |  |  |  |
| n | 258 | 250 |  |  |
| GMT (95%CI) | 2339.40(2051.00-2668.35) | 505.09(457.12-558.10) | 4.63(3.93-5.46) | <0.0001 |
| **Beta strain VS Prototype strain** |  |  |  |  |
| GMT ratio(95%CI) | 1.31(1.01-1.71) | 0.92(0.75-1.13) |  |  |
| *p* value | 0.0408 | 0.4387 |  |  |
| **Delta strain VS Prototype strain** |  |  |  |  |
| GMT ratio(95%CI) | 1.21(0.93-1.57) | 0.78(0.63-0.96) |  |  |
| *p* value | 0.1536 | 0.0217 |  |  |

Notes: “n” is the number of serum samples used in the test.
