# Supplementary material for: Safety and immunogenicity of a mosaic vaccine booster against Omicron and other SARS-CoV-2 variants: a randomized phase 2 trial
Source: Signal Transduct Target Ther. 2023 Jan 3;8:20. doi: 10.1038/s41392-022-01295-2 (PMC9808735; doi:10.1038/s41392-022-01295-2)
Supplement: Supplementary file 2 — Supplementary Protocol [file 41392_2022_1295_MOESM2_ESM.pdf]

# A Randomized, Double-Blind, Sequential Study to Evaluate the Safety, Immunogenicity, and Cellular Immunity of the Universal Recombinant COVID-19 (NVSI-06-09) Vaccine versus the Inactivated COVID-19 (Vero Cell) Vaccine in Adults Previously Immunized with the Vero Cell Vaccine

| | |
|---|---|
| Protocol Title: | A Randomized, Double-Blind, Sequential Study to Evaluate the Safety, Immunogenicity, and Cellular Immunity of the Universal Recombinant COVID-19 (NVSI-06-09) Vaccine versus the Inactivated COVID-19 (Vero Cell) Vaccine in Adults Previously Immunized with the Vero Cell Vaccine |
| Product Name: | Recombinant COVID-19 Vaccine (NVSI-06-09) |
| Specification: | 20ug/dose/0.5 ml |
| Protocol Number: | CNBG-REC-2022004 |
| Version Date: | 1st April 2022 |
| Version Number: | 1.0 |
| Sponsor (Seal): | China National Biotec Group Co., Ltd (CNBG)<br>Sinopharm CNBG National Vaccine & Serum Institute<br>Beijing Institute of Biological Products Co., Ltd<br>Lanzhou Institute of Biological Products Co., Ltd |
| Responsible Institute (seal): | |
| Statistical Institute (Seal): | |

CONFIDENTIAL

# TABLE OF CONTENTS


**TABLE OF CONTENTS** ........ 2

**STATEMENT OF COMPLIANCE** ........ 5

**INVESTIGATOR'S COMPLIANCE DECLARATION** ........ 6

**List of abbreviations** ........ 7

**Synopsis** ........ 9

**1. Introduction** ........ 17

**2. Background and Principle** ........ 17

- **2.1 Background of disease** ........ 17
- **2.2. Pathogenic background** ........ 18
- **2.3. Vaccine background** ........ 19

**3. Study Objective** ........ 21

- **3.1. Primary Objectives** ........ 21
- **3.2. Secondary Objectives** ........ 21
- **2.3 Exploratory Objectives** ........ 22

**4. Study Design** ........ 22

- **4.1. Trial Description** ........ 22
- **4.2. Procedures and Methodology** ........ 22
- **4.4 Trial Hypothesis** ........ 26
- **4.5 Safety Observation Indicators and Grading Criteria** ........ 26
- **4.6 Randomization and Blinding** ........ 32

**5. Screening and Withdrawal of Participants** ........ 34

- **5.1 Participant Population** ........ 34

**6. Product Introduction** ........ 36

- **6.1 Description and Characteristics of the Investigational Products** ........ 36
- **6.2 Vaccine Packaging and Labeling** ........ 38
- **6.3 Vaccine Storage and Transportation** ........ 39
- **6.4 Administration and Immunization Schedule** ........ 40

**7. Study Workflow and Visiting Method** ........ 40

- **7.1 Schedule of Visits** ........ 40

7.2 Study Procedure ........ 40
7.3 Safety Observation and Follow-up ........ 43
7.4 Laboratory Testing ........ 44
7.5 COVID-19 Case Monitoring ........ 49
7.6 Concomitant Medication ........ 51
7.7 Criteria for Suspension or Early Termination ........ 52
7.8 Protocol Violation and Deviation ........ 53
7.9 Study Duration ........ 53
7.10 Site Close ........ 53
8. Safety Definition, Reporting and Monitoring ........ 54
8.1 General Precautions ........ 54
8.2 Risk Prevention Measures Related to COVID-19 ........ 54
8.3 Handling and Reporting of Serious Adverse Events ........ 56
8.4. Outcome of Serious Adverse Events ........ 58
9. Completion of Clinical Trial ........ 58
10. Statistical Considerations ........ 59
10.1 Sample Size Considerations ........ 59
10.2 Analysis Set ........ 61
10.3 Statistical Method ........ 62
10.4 Subgroup Analysis ........ 63
10.5 Interim Analysis ........ 63
10.6 Multiplicity ........ 63
10.7 Handling of Missing Data ........ 63
11. Ethical and Regulatory Matters ........ 64
11.1. Ethics Committees and Regulatory Authorities ........ 64
11.2. Responsibilities of the Sponsor ........ 65
11.3. Responsibilities of the Investigator ........ 65
11.4. Participant Information and Informed Consent ........ 66
11.5. Compensation to Participants ........ 67
11.6. Participant Confidentiality ........ 68

11.7. **Amendment to Participant Related Information** .....68
11.8. **Direct Access to Source Documentation** .....68
**12. Study Management** .....69
12.1. **Case Report Form (eCRF) Handling** .....69
12.2. **Source Data and Participant Files** .....70
12.3. **Investigator Site File and Archiving** .....71
12.4. **Monitoring, Quality Assurance and RA Inspection** .....72
12.5. **Amendment to the Study Protocol** .....72
12.6. **Study Report and Publication Policy** .....73
**13. References** .....74

# STATEMENT OF COMPLIANCE

This study will be conducted in accordance compliance with the protocol, the International Conference on Harmonisation-Good Clinical Practice (ICH-GCP), the Declaration of Helsinki (with amendments) and local legal and regulatory requirements.

**Signature Page**

The signature below constitutes the approval of this protocol and the attachments, as well as provides the necessary assurances that this trial will be conducted according to all stipulations of the protocol, including all confidentiality statements, and according to Good Clinical Practice (GCP), Declaration of Helsinki (with amendments) and the laws and regulations of the countries in which the study takes place.

| | | |
|---|---|---|
| **Protocol name** | A Randomized, Double-Blind, Sequential Study to Evaluate the Safety, Immunogenicity, and Cellular Immunity of the Universal Recombinant COVID-19 (NVSI-06-09) Vaccine versus the Inactivated COVID-19 (Vero Cell) Vaccine in Adults Previously Immunized with the Vero Cell Vaccine | |
| **Protocol number** | CNBG-REC-2022004 | |
| **Version date** | 1st April 2022 | |
| **Version number** | Version 1.0 | |
| **Sponsor** | China National Biotec Group Co. Ltd. (CNBG)<br>Sinopharm CNBG National Vaccine & Serum Institute<br>Beijing Institute of Biological Products Co., Ltd<br>Lanzhou Institute of Biological Products Co., Ltd | |
| **Sponsor Project leader** | Name: Yang Yunkai<br>Company: China National Biotec Group Co., Ltd. (CNBG)<br>Address: No. B 2 Shuangqiao Road, Chaoyang District, Beijing, 100024 P.R. of China<br>Zip Code: 100024<br>Tel: 13601126881<br>E-mail: yangyunkai@sinopharm.com | Signature:<br><br>Date: |

CONFIDENTIAL

# INVESTIGATOR'S COMPLIANCE DECLARATION

- I have read this protocol and agree to conduct the study as outlined herein, and as implemented by any future protocol amendment/update, according to the terms of the clinical trial contract, and in accordance with any supplementary study: conduct procedures and/or guidance or documents of this study, complying with the obligations and requirements of clinical investigators and all other requirements listed in relevant national and international regulations including ICH GCP guidelines.8
- I assume responsibility for the compliance of the site personnel reporting to me or assisting me with the study.
- I confirm that I am aware of my obligations towards relevant regulatory authorities, as it concerns my participation in this study as a researcher.
- I agree to disclose and provide information to the Sponsor on any potential conflict of interest I may have to participate in this study as an investigator.
- I declare that I will co-operate with the Sponsor personnel and/or representatives, and vendors managing or supporting the study, including CRO, timely and adequately to ensure timely study conduct and compliance with study documents and relevant regulations.
- I am fully familiar with the correct method of using the vaccine described in the protocol, and other information provided by the Sponsor, including but not limited to the following contents: current investigator's brochure (IB) or equivalent documents and relevant supplements.
- I am familiar with and will abide by the GCP, the Guiding Principles for Quality Management of Vaccine Clinical Trials (Tentative) and all existing regulatory requirements.

| | |
|---|---|
| **Protocol Title** | A Randomized, Double-Blind, Sequential Study to Evaluate the Safety, Immunogenicity, and Cellular Immunity of the Universal Recombinant COVID-19 (NVSI-06-09) Vaccine versus the Inactivated COVID-19 (Vero Cell) Vaccine in Adults Previously Immunized with the Vero Cell Vaccine |
| **Protocol Number** | CNBG-REC-2022004 |
| **Version date** | 1st April 2022 |
| **Version Number** | Version 1.0 |

Name of Investigator (please print) ______________________

Investigator Signature: ________________________________

Date: ______________________________________________

CONFIDENTIAL

# LIST OF ABBREVIATIONS

| | |
|---|---|
| ACE2 | Angiotensin converting enzyme 2 |
| ADE | Antibody Dependence Enhancement |
| AE | Adverse Event |
| BCR | B-Cell Antigen Receptor |
| BP | Blood Pressure |
| CI | Confidence Interval |
| COA | Certificate of Analysis |
| CoV | Coronavirus |
| COVID-19 | Coronavirus disease of 2019 |
| DNA | Deoxyribonucleic acid |
| DSMB | Data and Safety Monitoring Board |
| eCRF | electronic Case Report Form |
| EDC | Electronic Data Capture System |
| EAC | Endpoint Assessment Committee |
| FAS | Full Analysis Set |
| GCP | Good Clinical Practice |
| GMP | Good Manufacturing Practice |
| GMT | Geometric Mean Titer |
| GMI | Geometric mean increase |
| IB | Investigator's Brochure |
| ICF | Informed Content Form |
| IEC | Independent Ethics Committee |
| MERS | Middle East Response Syndrome |
| NMPA | National Medical Products Administration |
| mFAS | modified Full Analysis Set |
| PPS | Per-Protocol Set |
| PCR | Polymerase Chain Reaction |
| RBD | Receptor Binding Domain |
| RNA | Ribonucleic acid |
| SAE | Serious Adverse Events |

CONFIDENTIAL

| | |
|---|---|
| SARS | Severe Acute Respiratory Syndrome |
| SOP | Standard Operating Procedure |
| SS | Safety Set |
| SUSAR | Suspected and Unexpected Serious Adverse Reactions |
| TCR | T-Cell Receptor |
| VED | Vaccine Enhanced Disease |
| WHO | World Health Organization |

CONFIDENTIAL

CONFIDENTIAL

# Synopsis

| | |
|---|---|
| **Study Title** | A Randomized, Double-Blind, SequentialStudy to Evaluate the Safety, Immunogenicity, and Cellular Immunity of the Universal Recombinant COVID-19 (NVSI-06-09) Vaccine versus the Inactivated COVID-19 (Vero Cell) Vaccine in Adults Previously Immunized with the Vero Cell Vaccine |
| **Product Characteristic** | The universal recombinant protein used to develop the COVID-19 NVSI-06-09 vaccine was expressed by recombinant Chinese hamster ovary (CHO) cells and designed based on the receptor-binding domain (RBD) of SARS-CoV-2 Spike (S) protein. Engineered cells were cultured, harvested and purified by a series of processes, and then added with aluminum hydroxide adjuvant. The product is a universal recombinant COVID-19 vaccine against major variants such as the SARS-CoV-2 Omicron variant. It is developed through in-depth research on virus structure, mutation pattern and mutation sites, especially based on the analysis of mutations at the receptor-binding domain of the SARS-CoV-2 Omicron variant. |
| **Indications** | The universal recombinant COVID-19 (NVSI-06-09) vaccine can be used to immunize adults aged 18 years and above. Immunization with this vaccine can stimulate the body to produce an immune response to SARS-CoV-2 for the prevention of COVID-19 infection. |
| **Study Design** | This is a randomized, double-blind parallel, and positive controlled study design. Participants who have been previously vaccinated with two or three doses of Vero cell vaccine (≥6 months prior to the study screening visit) will receive one booster dose of either NVSI-06-09 vaccine or Vero Cell vaccine.<br><br>**Sample Size**: A total of 516 participants will be randomly assigned to one of two study groups (NVSI-06-09 vaccine group or Vero Cell vaccine group) in a 1:1 ratio, with 258 participants in each group.<br><br>**Immunization Schedule:** Participants will be immunized with one booster dose of either NVSI-06-09 vaccine or Vero Cell vaccine. |

CONFIDENTIAL

**Route of Administration**: Vaccines will be administed via intramuscular injection in the lateral deltoid muscle of the upper arm.

**Safety Observation:** The participants will be observed at the study site for 30 minutes after immunization administration. Local and systemic adverse events will be collected and recorded. Within 0-28 days after booster vaccination, the local and systemic reactions of the participants will be actively followed up and recorded on the diary card/contact card. Serious adverse events (SAEs) will be collected up to 12 months following administration of the booster vaccine.

**Immunogenicity Observation:** Blood samples (12.5ml) will be collected from all participants and serum will be separated. The neutralizing antibody will be tested by a micro-dose cytopathogenic effect (CPE) assay. Time points of blood sample collection are described in Table 1 (Sample Size and Procedures of Clinical Trial).

**Cellular Immunity Observation:** A total of 20 participants from each study group will be sequentialy invited to give 5 additional venous blood samples (12ml per participant per sample). Peripheral blood mononuclear cells (PBMC) are separated, and the activation of specific T cells are detected by enzyme-linked immunospot (ELISPOT) and flow cytometry. T-cell receptor (TCR) characteristics of specific T cells and B-cell antigen receptor (BCR) characteristics of B cells are analyzed using high-throughput single-cell sequencing to analyze the type and proportion of immune cells in PBMC, as well as the expression profile of immune-related genes in each subgroup of cells.

**Table 1 Sample Size and Procedures of Clinical Trial**

| Previous Immunization (No. doses and timeline) | Intervention Group (Booster Vaccine) | Sample Size (*N*) | Safety | Immunogenicity Blood Collection Time Point | Cellular immunity Blood Collection Time Point |
|---|---|---|---|---|---|

CONFIDENTIAL

| | | | | | |
|---|---|---|---|---|---|
| Vero Cell Vaccine (2 or 3 doses, ≥6 months prior to study screening) | NVSI-06-09 Vaccine | 258 | Safety data is collected after booster vaccine administration.<br><br>Solicited AEs collected within 30 minutes via onsite observation and 0-7 days via diary cards.<br><br>Unsolicited AEs collected within 8-30 days via safety visits.<br><br>SAEs collected up to 12 months following the booster dose via safety visits. | Blood samples are collected during the following study visits:<br><br>Before booster vaccine administration:<br>• Visit 1 (D0)*<br><br>After booster vaccine administration:<br>• Visit 4 (D14) [a]<br>• Visit 6 (D28) [a]<br>• Visit 8 (3M after V1) [b]<br>• Visit 11 (6M after V1) [b]<br>• Visit 12 (9M after V1) [b]<br>• Visit 13 (12M after V1) [b] | Blood samples are collected during the following study visits:<br><br>Before booster vaccine administration:<br>• Visit 1 (D0) *<br><br>After booster vaccine administration:<br>• Visit 4 (D14) [a]<br>• Visit 6 (D28) [a]<br>Visit 11 (D28) (6M after V1) [b] |
| | Vero Cell Vaccine | 258 | | | |
| Total sample size (*N*) | | 516 | | | |

**Abbreviations: N: number, D: days, M, months.**

[a] Blood collection window at D14 and D28 is +10 days

[b] Blood collection window at 3M, 6M, 9M, 12M is +30 days.

**Study Objectives & Endpoints**

**Primary Objectives:**

1. To compare the immunogenicity (prototype SARS-CoV-2 Omicron variant stain) of NVSI-06-09 vaccine with Vero Cell vaccine, 14 days after a single dose of the booster vaccine in adults ≥18 years of age.
2. To compare the safety of NVSI-06-09 vaccine with Vero Cell vaccine, 28 days after a single dose of the booster vaccine in adults ≥18 years of age.

**Primary Endpoints:**

1. GMT and the 4-fold rise rate of anti-omicron neutralizing antibody 14 days after a

CONFIDENTIAL

| | |
|---|---|
| | single dose of the booster vaccine in adults ≥18 years of age.<br>2. The incidence and severity of any adverse reactions/events within 30 minutes after vaccination;<br>3. The incidence and severity of solicited adverse reactions/events within 0-7 days after vaccination;<br>4. The incidence and severity of unsolicited adverse reactions/events within 8-30 days after vaccination;<br>5. The incidence of SAE and AESI observed after vaccination and up to 12 months after full course of immunization.<br><br>**Secondary Objectives:**<br>1. To compare the immunogenicity (prototype SARS-CoV-2 Omicron variant stain) of NVSI-06-09 vaccine with Vero Cell vaccine, 28 days after a single dose of the booster vaccine in adults ≥18 years of age.<br>2. To compare the immune persistence of booster immunization using recombinant COVID-19 vaccine (NVSI-06-09) and inactivated COVID-19 vaccine (Vero cell) in populations≥18 years of age.<br>3. To compare cellular immunity after immunization with NVSI-06-09 vaccine or Vero Cell vaccine in adults ≥18 years of age.<br><br>**Secondary endpoint:**<br>1. GMT and 4-fold rise rate of anti-omicron neutralizing antibody, 28 days after a single dose of the booster vaccine in adults ≥18 years of age.<br>2. GMT of anti-omicron IgG antibody, 4-fold rise rate, GMI, and proportions of neutralizing antibody tittered ≥ 1: 16, ≥ 1: 32 and ≥ 1: 64 before booster vaccination and 28 days after a single dose of the booster vaccine in adults ≥18 years of age.<br>3. GMTs of anti-omicron neutralizing antibody and IgG antibody and the proportions of neutralizing antibody titered ≥ 1: 16, ≥ 1: 32 and ≥ 1: 64 in 3 months, 6 |

CONFIDENTIAL

| | |
|---|---|
| | months, 9 months and 12 months after full course immunization (i.e., 2 or 3 previous Vero Cell vaccine doses and 1 booster dose).<br><br>**Exploratory Objectives:**<br>1. To evaluate the efficacy of NVSI-06-09 vaccine against COVID-19, especially severe cases (including deaths), 14 days after booster vaccine administration.<br>2. To compare the cross-protection effects of NVSI-06-09 vaccine against COVID-19 Variants of Concern (VoC) such as (Alpha, Beta, Delta, and Omicron), 14 and 28 days after a single dose of NVSI-06-09 administration.<br><br>**Exploratory Endpoints**<br>1. The efficacy of NVSI-06-09 vaccine against omicron-caused COVID-19, especially severe cases (including deaths), 14 days after booster vaccination in adults ⩾18 years of age.<br>2. Cross-protecting effect of neutralizing antibodies against different variants (Alpha, Beta, Delta, and Omicron), 14 days after a single dose of the booster vaccine in adults ⩾18 years of age.<br>**3.** Cross-protecting effect of neutralizing antibodies against different variants (Alpha, Beta, Delta, and Omicron), 28 days after a single dose of the booster vaccine in adults ⩾18 years of age. |

CONFIDENTIAL

| | |
|---|---|
| **Investigational Vaccine** | **Investigational Vaccine**: Recombinant COVID-19 Vaccine (NVSI-06-09)<br>**Manufacturer**: Sinopharm CNBG National Vaccine & Serum Institute<br>Lanzhou Institute of Biological Products Co., Ltd<br>Beijing Institute of Biological Products Co., Ltd.<br>**Specifications**: 0.5 ml/vial. The dose for human use is 0.5 mL per time, which contains 20ug of SARS-CoV-2 antigen.<br>**Storage Condition**: 2-8 ℃<br>**Batch Number**: xxxxxxx **Expiry Date**: xxxxxxx<br><br>**Control Vaccine**: Inactivated SARS-CoV-2 Vaccine (Vero cell)<br>**Manufacturer**: Beijing Institute of Biological Products Co., Ltd.<br>**Specifications**: 0.5 ml/vial. The dose for human use is 0.5 mL per time, which contains 6.5U of inactivated SARS-CoV-2 antigen.<br>**Storage Condition**: 2-8 ℃<br>**Batch Number**: xxxxxxx **Expiry Date**: xxxxxxx |
| **Inclusion Criteria** | Individuals are eligible to participate in this study if all of the following criteria apply:<br>- Age: ≥18 years;<br>- Judged by the investigator that the health condition is well after inquiry and physical examination;<br>- Vaccinated with 2 or 3 doses of inactivated COVID-19 vaccine (Vero Cell vaccine) ≥6 months prior to study screening and according to product insert;<br>- Female participants who are not pregnant or nursing or at the time of enrolment (confirmed via negative urine pregnancy test), and do not have plans to become pregnant within the first 6 months after enrollment. Effective contraceptive measures have been taken within 2 weeks before study inclusion and initiation;<br>- Be able and willing to provide written informed consent to participate in the study and complete all study requirements according to the study protocol; |

CONFIDENTIAL

| | |
|---|---|
| **Exclusion Criteria** | Individuals are not eligible to participate in this study and are therefore excluded from study participation if one or more of the following criteria apply:<br>- COVID-19 infection positive patients (including suspected or asymptomatic cases);<br>- Have a history of SARS and MERS infection;<br>- Have been vaccinated by any COVID-19 Vaccines other than Vero Cell vaccine<br>- Have an axillary temperature ≥37.3℃ (forehead temperature ≥37.8℃);<br>- Have had previous allergic reactions to vaccination (such as acute allergic reactions, urticaria, eczema, dyspnea, angioneurotic oedema or abdominal pain) or allergy to known components of COVID-19 vaccine;<br>- History of thrombocytopenia or other coagulation disorders;<br>- Patients with known immunological impairment or immunocompromised.<br>- Received whole blood, blood products, plasma and/or immunoglobulin therapy within 3 months before study enrollment<br>- Have known or suspected severe illness such as respiratory illness, acute infection or active attacks of chronic illness, liver and kidney disease, severe diabetes mellitus, malignant tumour, infectious or allergic skin disease, human immunodeficiency virus (HIV) infection (test report available);<br>- Diagnosed with serious cardiovascular diseases such as cardiopulmonary failure, drug-uncontrolled hypertension (Systolic blood pressure ≥160 mmHg and/or diastolic blood pressure ≥ 95 mmHg).<br>- Received live attenuated vaccines within 1 month before study enrollment;<br>- Received inactivated vaccines within 14 days before study enrollment;<br>- Received other investigational drugs within 6 months before study enrollment;<br>- Other vaccination-related contraindications considered by investigators. |
| **Participant's** | Early withdrawal means that the participant fails to complete the booster vaccination and blood collection procedures according to the clinical study protocol and permanently discontinues study participation.<br>When any of the following conditions occur, the participant will be withdrawn from the |

CONFIDENTIAL

| | |
|---|---|
| **early withdrawal** | study.<br>- The participant requests to withdraw from the clinical trial;<br>- Intolerable adverse events, whether related to the investigational drug or not;<br>- The health status of the participant does not allow them to continue to participate in this trial;<br>- The participant is vaccinated with other clinical investigational vaccines during the study period;<br>- Any other reason that investigator considers. |
| **Criteria for suspension and early termination** | In case of any of the following circumstances, the trial shall be suspended, the investigator, the Sponsor and the Ethics Committee shall jointly hold a meeting to decide whether to terminate the clinical trial early:<br>- The number of participants with adverse reactions of severity rated Grade 3 or above exceeds 15% of the participants study vaccinated in this subgroup;<br>- One case of Grade 4 adverse reaction related to vaccination or a suspected unexpected serious adverse reaction (SUSAR) has occurred.<br><br>In case of any of the following circumstances, the clinical trial shall be terminated early:<br>- The Sponsor has found that the vaccine has potential safety hazards or the trial has quality-related problems and requires the complete termination of the study.<br>- The Ethics Committee requests to terminate the trial because of the ethical violation in the trial.<br>- The administrative authority requests to terminate the trial. |
| **Trial Completion** | Trial sites will be closed and immune persistence evaluation will be initiated only once the following activities have been completed:<br>1. Completed all study requirements according to the study protocol<br>2. Locked the database<br>3. Clarification of data by the statistician (blind audit)<br>4. Unblinded the necessary study staff to evaluate the safety, immunogenicity and vaccine efficacy. |

CONFIDENTIAL

CONFIDENTIAL

# A Randomized, Double-Blind, Sequential Study to Evaluate the Safety, Immunogenicity, and Cellular Immunity of the Universal Recombinant COVID-19 (NVSI-06-09) Vaccine versus the Inactivated COVID-19 (Vero Cell) Vaccine in Adults Previously Immunized with the Vero Cell Vaccine

## 1. INTRODUCTION

The recombinant COVID-19 vaccine (NVSI-06-09) was developed by Sinopharm CNBG National Vaccine & Serum Institute (NVSI) affiliated to China National Biotec Group Co., Ltd. (CNBG) is used to prevent the diseases caused by SARS-CoV-2. NVSI, Lanzhou Institute of Biological Products Co., Ltd. (hereinafter referred to as "LIBP") and Beijing Institute of Biological Products Co., Ltd. (hereinafter referred to as "BIBP") affiliated to CNBG, jointly applied as Sponsors to carry out clinical trials on this product.

In this project, a randomized, blinded, positive control method will be used to evaluate the safety and immunogenicity of booster immunization of Recombinant COVID-19 Vaccine (NVSI-06-09) in a healthy population aged 18 years and above. This study was approved by the Ministry of Health of UAE.

## 2. BACKGROUND AND PRINCIPLE

### 2.1 Background of disease

According to WHO data, as of January 10, 2022, there were 305,914,601 confirmed cases and 5,486,304 deaths due to COVID-19, with a mortality rate of 1.8%. The infection sources of COVID-19 are mainly SARS-CoV-2 infected patients and asymptomatic infected persons, but it is found that patients in the incubation period are also contagious, and they are highly contagious within 5 days after onset. SARS-CoV-2 is mainly transmitted through respiratory droplets and close contact, which may be transmitted by aerosol. Contact with virus-contaminated articles can also cause infection. Because SARS-CoV-2 can be isolated from patients' faeces and urine, environmental pollution caused by faeces and urine can also cause aerosol or contact transmission. People are generally susceptible, and they can become infected at any age. People can get certain

CONFIDENTIAL

immunity after infection or vaccination with the COVID-19 vaccine, but the duration is not clear. Based on the current epidemiological investigation, the incubation period of the virus is generally 1 ~ 14 days, mostly 3 ~ 7 days, and the incubation period of very few cases can reach 24 days.

The main clinical symptoms of COVID-19 are fever, dry cough and fatigue, and the first symptoms of some patients are loss of smell and taste. A few patients are accompanied by nasal congestion, runny nose, sore throat, muscle pain and diarrhoea. According to clinical symptoms, it can be divided into four categories: mild, moderate, severe and critical. Severe patients often have dyspnea and/or hypoxemia one week after onset, and severe patients can rapidly progress to acute respiratory distress syndrome, septic shock, metabolic acidosis, coagulation dysfunction and multiple organ failure. Very few patients may also have central nervous system involvement and microvascular necrosis. It is worth noting that severe and critical patients may have moderate to low fever or even no obvious fever during the disease course. Mild patients may show low fever, slight fatigue, smell and taste disorders, etc., without pneumonia. A few patients have no obvious clinical symptoms after being infected with SARS-CoV-2. According to the current cases, most patients have a good prognosis, while a few patients are in critical condition. The prognosis of the elderly and those with chronic basic diseases is poor. The clinical process of pregnant women with COVID-19 is similar to that of patients of the same age. Paediatric patients have relatively mild symptoms.

## 2.2. Pathogenic background

SARS-CoV-2 belongs to β coronavirus. Under a cryoelectron microscope, it is approximately spherical particles with variable diameters, mainly around 100nm. SARS-CoV-2 contains a dense virus matrix, surrounded by a lipid bilayer, and its outer surface protrudes. It is an enveloped virus, which is composed of non-segmental positive-sense single-stranded RNA and protein. It belongs to Orthocoronavirinae and is widely distributed in humans and other mammals.

The genome of the SARS-CoV-2 strain is 29.9 kb long and encodes 9860 amino acids. SARS-CoV-2 has four main structural proteins, known as spike protein (S), membrane protein (M), an envelope protein (E) and nucleocapsid protein (N), as well as several helper proteins. S, M and E proteins constitute the viral envelope and are the main surface antigens of a virus-induced immune

CONFIDENTIAL

response. S protein is a transmembrane glycoprotein with a molecular weight of about 150kDa, which is divided into two subunits, namely S1 and S2, by the Furin protease of host cells. S1 contains one RBD, which is responsible for determining the cell-targeting between host cells and virus receptor binding domains; As a membrane fusion subunit, S2 can mediate virus to fuse with host cells. M protein plays an important role in determining the shape of the viral envelope. It can bind with other structural proteins, and binding with N protein helps to stabilize nucleocapsid and promote the assembly of the virus. E protein plays an important role in the process of virus production and maturation. N protein is located in the core of the virus and combines with viral RNA to form nucleocapsid. N protein has a highly conserved sequence, and it participates in virus replication and infection of host cells.

The binding of spike protein (S protein) with angiotensin-converting enzyme 2 (ACE2) protein is essential for SARS-CoV-2 infecting human cells. After fusion, transmembrane protein serine 2 (TMPRSS2), which exists on the surface of host cells, scavenges ACE2 and activates S protein. Activation of the S protein causes a conformational change and allows the virus to enter the host cell. The affinity of S protein of SARS-CoV-2 with ACE2 is 10 to 20 times that of the SARS virus, which may be the reason for the strong transmission of COVID-19. ACE2 expressed in respiratory epithelial cells and type II alveolar cells of the lung is the receptor of SARS-CoV-2 entering host cells. Besides lung, ACE2 is also distributed in intestinal epithelial cells of the ileum and colon, vascular smooth muscle cells, proximal tubular epithelial cells of the kidney, urinary tract epithelial cells of the bladder and testis. Therefore, COVID-19 patients will not only have respiratory diseases, but also have heart, kidney, and digestive system diseases. ACE2 expression in vascular intima can damage endothelial cells and lead to the risk of thrombotic events. In addition, the immune system releases a large number of cytokines to viral infection sites or secondary infection, which can induce cytokine storm and septicemia symptoms.

In addition, coronavirus is sensitive to ultraviolet rays and heat. For 30 minutes at 56 ℃, ether, 75% ethanol, chlorine-containing disinfectant, peracetic acid and chloroform can effectively inactivate the virus, but chlorhexidine cannot effectively inactivate the virus.

### 2.3. Vaccine background

CONFIDENTIAL

As of January 2022, there are 36 COVID-19 vaccines have been approved for use in at least one country/region around the world, including 3 mRNA vaccines, 6 viral vector vaccines, 11 inactivated vaccines, 15 recombinant protein vaccines, and 1 DNA vaccine.

According to the statistics from the World Health Organization (WHO) as of January 7th, 2022, there are 331 COVID-19 candidate vaccines active in the R&D stage, among which 137 are at the clinical development stage. There are 146 recombinant protein vaccines, which accounted for 44.1% of all types of vaccines, 47 of which are at the clinical stage. A total of 7 vaccines in China have obtained conditional approval or emergency use authorization, including 5 inactivated vaccines (Sinopharm CNBG BIBP, WIBP, Sinovac, Kangtai Bio and Institute of Medical Biology, Chinese Academy of Medical Sciences), Cansino's recombinant adenovirus vector vaccine (injection type) and Zhifei biological's recombinant protein vaccine (CHO cell). Among them, two inactivated vaccines produced by CNBG-BIBP and Sinovac have been approved by WHO in 2021 and included in the emergency use listing. The recombinant protein vaccine developed by Zhifei Bio has been authorized for emergency use in China, and others are in Phase I/II study. And four inactivated vaccines, four recombinant viral vector vaccines and four nucleic acid vaccines are in Phase III clinical trial.

### 2.3.1 Inactivated Vaccine

The inactivated vaccine is made from a virus that has been grown in cell culture and have been lose its pathogenicity by physical or chemical method but only retain its antigenicity. As a traditional vaccine preparation method, the process is mature. At present, there are 29 inactivated vaccines under research around the world, of which 18 are at the clinical stage. Five inactivated vaccines in China have been approved for conditional marketing or emergency use authorization, including Sinopharm CNBG BIBP, WIBP, Sinovac, Kangtai Bio and Institute of Medical Biology, Chinese Academy of Medical Sciences.

### 2.3.2 Recombinant Protein Vaccine

Compared with other types of vaccines, recombinant protein vaccines are one of the key directions of research and development in novel vaccines due to the significant advantages in safety and low cost. At present, 146 COVID-19 recombinant protein vaccine candidates are under development,

CONFIDENTIAL

and 47 of them are at the clinical stage.

### 2.3.3 Recombinant Viral Vector Vaccine

Viral vector vaccine uses replicative or non-replicative viruses as vectors, recombine specific antigen gene fragments into the vector genome, deliver them into host cells, and induce expression and induces a corresponding immune response. Currently, there are 97 recombinant viral vector vaccines in R&D, 27 of them are at the clinical stage, 6 vaccines have been approved for use in at least one country or region.

### 2.3. 4 Nucleic Acid Vaccine

Nucleic acid vaccines introduce DNA or mRNA encoding specific antigens into human cells, express corresponding antigen proteins, and induce an immune response. Currently, there are 78 nucleic acid vaccines in active research and development, and 38 vaccines at the clinical stage, including 23 mRNA vaccines and 15 DNA vaccines.

# 3. STUDY OBJECTIVE

To evaluate the safety and immunogenicity of booster vaccination of Recombinant COVID-19 Vaccine (NVSI-06-09) developed by Sinopharm CNBG National Vaccine & Serum Institute in a healthy population aged 18 years and above.

## 3.1. Primary Objectives

1. To compare the immunogenicity (prototype SARS-CoV-2 Omicron variant stain) of NVSI-06-09 vaccine with Vero Cell vaccine, 14 days after a single dose of the booster vaccine in adults ⩾18 years of age.
2. To compare the safety of NVSI-06-09 vaccine with Vero Cell vaccine, 28 days after a single dose of the booster vaccine in adults ⩾18 years of age.

## 3.2. Secondary Objectives

1. To compare the immunogenicity (prototype SARS-CoV-2 Omicron variant stain) of NVSI-06-09 vaccine with Vero Cell vaccine, 28 days after a single dose of the booster vaccine in

CONFIDENTIAL

adults ⩾18 years of age.

2. To compare the immune persistence of booster immunization using recombinant COVID-19 vaccine (NVSI-06-09) and inactivated COVID-19 vaccine (Vero cell) in populations⩾18 years of age.
3. To compare cellular immunity after immunization with NVSI-06-09 vaccine or Vero Cell vaccine in adults ⩾18 years of age.

### 2.3 Exploratory Objectives

1. To evaluate the efficacy of NVSI-06-09 vaccine against COVID-19, especially severe cases (including deaths), 14 days after booster vaccine administration.
2. To compare the cross-protection effects effects of NVSI-06-09 vaccine against COVID-19 Variants of Concern (VoC) such as Alpha, Beta, Delta, and Omicron), 14 and 28 days after a single dose of NVSI-06-09 administration.

## 4. STUDY DESIGN

### 4.1. Trial Description

This clinical trial adopts a randomized, double-blind and positive controlled design.

### 4.2. Procedures and Methodology

**Sample size:**

A total of 516 healthy participants aged 18 years and above who have been vaccinated with 2/3 doses of inactivated COVID-19 vaccine, will be enrolled, and randomly assigned to the experimental group and control group in a 1:1 ratio, with 258 participants in each booster group.

**Immunization Schedule:**

Among the participants who have been vaccinated with two/three doses of inactivated COVID-19 vaccine (Vero cell) for ≥6 months, one dose of recombinant COVID-19 vaccine (NVSI-06-09) (NVSI-06-09) or inactivated COVID-19 vaccine (VERO cell) will be immunized boosterly.

**Route of Administration**:

CONFIDENTIAL

Lateral deltoid muscle of the upper arm, intramuscular injection

**Safety Observation:**

The participants will be observed at the study site for 30 minutes after immunization, and local and systemic adverse events will be actively collected and recorded on the diary cards within 0-7 days. Perform one safety follow-up per week to collect AEs and record them in the follow-up system. Serious adverse events (SAEs) will be monitored within 12 months after immunization and follow-up, recorded and report according to requirements.

**Immunogenicity Observation:**

Blood samples of all the participants will be collected before the vaccination of booster dose and 14 days, 28 days, 3 months, 6 months, 9 months and 12 months after booster vaccination for detection of neutralizing antibodies. The neutralizing antibody will be tested by a micro-dose cytopathogenic effect (CPE) assay.

**Cellular Immunity Observation:**

A total of 20 participants from each study group will be sequentially invited to give 5 additional venous blood samples (12ml per participant per sample). PBMC was separated, and the activation of specific T cells were detected by ELISPOT and flow cytometry. T-cell receptor (TCR) characteristics of specific T cells and B-cell antigen receptor (BCR) characteristics of B cells are analyzed using high-throughput single-cell sequencing to analyze the type and proportion of immune cells in PBMC, as well as the expression profile of immune-related genes in each subgroup of cells. Specific time points of blood sample collection are described in Table 2 Sample Size and Procedures of Clinical Trial.

**Table 2. Sample size and Procedures of Clinical Trial**

| Previous Immunization (No. doses and timeline) | Intervention Group (Booster Vaccine) | Sample Size (*N*) | Safety | Immunogenicity Blood Collection Time Point | Cellular immunity Blood Collection Time Point |
|---|---|---|---|---|---|

CONFIDENTIAL

| | | | | | |
|---|---|---|---|---|---|
| Vero Cell Vaccine (2 or 3 doses, ≥6 months prior to study screening) | NVSI-06-09 Vaccine | 258 | Safety data is collected after booster vaccine administration.<br><br>Solicited AEs collected within 30 minutes via onsite observation and 0-7 days via diary cards.<br><br>Unsolicited AEs collected within 8-30 days via safety visits.<br><br>SAEs collected up to 12 months following the booster dose via safety visits. | Blood samples are collected during the following study visits:<br><br>Before booster vaccine administration:<br>• Visit 1 (D0)*<br><br>After booster vaccine administration:<br>• Visit 4 (D14) [a]<br>• Visit 6 (D28) [a]<br>• Visit 8 (3M after V1) [b]<br>• Visit 11 (6M after V1) [b]<br>• Visit 12 (9M after V1) [b]<br>• Visit 13 (9M after V1) [b] | Blood samples are collected during the following study visits:<br><br>Before booster vaccine administration:<br>• Visit 1 (D0) *<br><br>After booster vaccine administration:<br>• Visit 4 (D14) [a]<br>• Visit 6 (D28) [a]<br>Visit 11 (D28) (6M after V1) [b] |
| | Vero Cell Vaccine | 258 | | | |
| Total sample size (*N*) | | 516 | | | |

**Abbreviations: N: number, D: days, M, months.**

[a] Blood collection window at D14 and D28 is +10 days

[b] Blood collection window at 3M, 6M, 9M, 12M is +30 days.

### 4.3. Study Endpoint

CONFIDENTIAL

**Primary Endpoints:**

1. GMT and the 4-fold rise rate of anti-omicron neutralizing antibody 14 days after a single dose of the booster vaccine in adults ≥18 years of age.
2. The incidence and severity of any adverse reactions/events within 30 minutes after vaccination;
3. The incidence and severity of solicited adverse reactions/events within 0-7 days after vaccination;
4. The incidence and severity of unsolicited adverse reactions/events within 8-30 days after vaccination;
5. The incidence of SAE and AESI observed after vaccination and up to 12 months after full course of immunization.

**Secondary Endpoints:**

1. GMT and 4-fold rise rate of anti-omicron neutralizing antibody, 28 days after a single dose of the booster vaccine in adults ≥18 years of age.
2. GMT of anti-omicron IgG antibody, 4-fold rise rate, GMI, and proportions of neutralizing antibody tittered ≥ 1: 16, ≥ 1: 32 and ≥ 1: 64 before booster vaccination and 28 days after a single dose of the booster vaccine in adults ≥18 years of age.
3. GMTs of anti-omicron neutralizing antibody and IgG antibody and the proportions of neutralizing antibody titered ≥ 1: 16, ≥ 1: 32 and ≥ 1: 64 in 3 months, 6 months, 9 months and 12 months after full course immunization (i.e., 2 or 3 previous Vero Cell vaccine doses and 1 booster dose).

**Exploratory Endpoints**

1. The efficacy of NVSI-06-09 vaccine against omicron-caused COVID-19, especially severe cases (including deaths), 14 days after booster vaccination in adults ≥18 years of age.
2. Cross-protecting effect of neutralizing antibodies against different variants (Alpha, Beta, Delta, and Omicron), 14 days after a single dose of the booster vaccine in adults ≥18

CONFIDENTIAL

years of age.

3. Cross-protecting effect of neutralizing antibodies against different variants (Alpha, Beta, Delta, and Omicron ), 28 days after a single dose of the booster vaccine in adults ⩾18 years of age.

## 4.4 Trial Hypothesis

1. The 4-fold rise rate and GMT (Omicron) of neutralizing antibody of Recombinant COVID-19 Vaccine (NVSI-06-09) after booster vaccination with one dose of vaccine are superior to that of Inactivated COVID-19 Vaccine (Vero cell), H0: $\delta \leq 0.0212$ vs. H1: $\delta > 0.0212$ (both are a logarithmic scale, equivalent to GMT ratio 95%CI >1.05).
2. After booster vaccination of one dose of Recombinant COVID-19 Vaccine (NVSI-06-09), the 4-fold rise rate and GMT (Omicron) of neutralizing antibody is not inferior to COVID-19 inactivated vaccine (Vero cell), and the non-inferior threshold is 2/3 (GMT ratio; the difference on log10 scale is-0.176). If the lower bound of GMT ratio 95% CI is > 2/3, the hypothesis of non-inferiority in post-immunization GMT between experimental group and control group will be concluded.

## 4.5 Safety Observation Indicators and Grading Criteria

### 4.5.1 Safety Observation Indicators

**(1) Adverse events collected in 0-7 days**

***Local adverse events***: pain, induration, swelling, rash, redness and pruritis.

***Systemic adverse events***: fever, diarrhoea, constipation, dysphagia, anorexia, vomiting, nausea, myalgia (systemic), arthralgia, headache, cough, dyspnoea, systemic pruritis (no skin damage), abnormal skin mucosa, acute allergic reactions, tiredness/fatigue, and dizziness

***Other adverse events:*** any medical events other than the above mentioned during clinical studies, such as acute diseases, accidental injuries, etc.

**(2) Adverse events collected in 8-28 days**

CONFIDENTIAL

Any medical events, such as acute diseases and accidental injuries occurred during 8~28 days in clinical studies.

**(3) Pregnancy events**

Collect all pregnancy events and outcomes between vaccination and the end of the study (6 months after a full course of immunization). Follow-up of the newborns should last at least 1 month after birth.

### 4.5.2 Adverse Event Grading Criteria

Local adverse events, systemic adverse events, vital signs, and laboratory testing parameters after vaccination were determined based on the *Guiding Principles of Adverse Events Grading Criteria for Clinical Trials of Prophylactic Vaccines*.

CONFIDENTIAL

## Table 3 Local Adverse Events Grading Table

| Symptoms/signs | Grade 1 | Grade 2 | Grade 3 | Grade 4 |
|---|---|---|---|---|
| Pain | Do not or slightly affect physical activities | Affect physical activities | Affect daily life | Loss of basic self-care ability, or hospitalization |
| Induration *, swelling * * # | 2.5 ~ < 5 cm in diameter or 6.25 ~ < 25 $cm^2$ in area not or slightly affecting daily life | 5 ~ <10cm in diameter or 25 ~ < 100$cm^2$ in area or affecting daily life | Diameter ≥10 cm or area ≥100 $cm^2$ or ulceration or secondary infection or phlebitis or sterile abscess or wound drainage or seriously affect daily life | Abscess, exfoliative dermatitis, dermal or deep tissue necrosis |
| Rash *, flush * * # | 2.5 ~ < 5 cm in diameter or 6.25 ~ < 25 $cm^2$ in area not or slightly affecting daily life | 5 ~ < 10 cm in diameter or 25 ~ < 100 $cm^2$ in area or affecting daily life | Diameter ≥10 cm or area ≥100 $cm^2$ or ulceration or secondary infection or phlebitis or sterile abscess or wound drainage or seriously affect daily life | Abscess, exfoliative dermatitis, dermal or deep tissue necrosis |
| Pruritus | Itching at the vaccination site was relieved by itself or within 48 hours after treatment | Itching at the vaccination site did not relieve within 48 hours after treatment | Affect daily life | NA |

Note:

* In addition to measuring the diameter directly for grading evaluation, the progress and change of measurement results should also be recorded.

** Maximum measured diameter or area shall be used.

# The evaluation and grading of induration and swelling, rash and redness should be based on the functional grading and actual measurement, with higher grading, being selected.

When judging severity, it can be judged as a next grade adverse event only when it meets the grading criteria of the previous one.

CONFIDENTIAL

CONFIDENTIAL

**Table 4 Systemic Adverse Events Grading Table**

| Physical Sign | Grade 1 | Grade 2 | Grade 3 | Grade 4 |
|---|---|---|---|---|
| Fever * (axillary temperature ℃) | 37.3 ~ < 38.0 | 38.0 ~ < 38.5 | 38.5 ~ < 39.5 | ≥ 39.5 for more than 3 days |
| Diarrhoea | Mild or transient, 3 ~ 4 times/day, abnormal faecal characteristics, or mild diarrhoea lasting less than 1 week | Moderate or persistent, 5 ~ 7 times/day, abnormal faecal characteristics, or diarrhoea> 1 week | > 7 times/day, abnormal faecal characteristics, hemorrhagic diarrhoea, orthostatic hypotension, and electrolyte imbalance, requiring intravenous infusion > 2L | Hypotensive shock, requiring hospitalization |
| Constipation ** | Need faecal softener and dietary adjustment | Need laxative drugs | Stubborn constipation requires manual dredging or enema | Toxic megacolon or intestinal obstruction |
| Dysphagia | Mild discomfort when swallowing | Restricted diet | Diet and conversation are very limited; can't eat solid food | Can't eat liquid food; Need intravenous nutrition |
| Anorexia | Loss of appetite, but no reduction in food intake | Loss of appetite, food intake decreased, but body weight did not decrease significantly | Loss of appetite and obvious weight loss | Need measures to intervene (e.g., gastric tube feeding, parenteral nutrition) |
| Vomiting | 1 ~ 2 times/24 hours without affecting activities | 3 ~ 5 times/24 hours or limited activity | > 6 times within 24 hours or intravenous rehydration is required | hospitalization or other nutrition due to hypotensive shock |
| Nausea | Transient (< 24 hours) or intermittent and normal food intake | Persistent nausea leads to reduced food intake (24 ~ 48 hours) | Persistent nausea leads to almost no food intake (> 48 hours) or needs for intravenous rehydration | Life-Threatening |
| Myalgia (local) | not affect daily activities | Slightly affect daily activities | Severe myalgia seriously affects daily activities | Emergency or hospitalization |

CONFIDENTIAL

| | | | | |
|---|---|---|---|---|
| Arthralgia | Mild pain without hindering the function | Moderate pain, need analgesics and/or pain interferes with function but does not affect daily activities | Severe pain, need for painkillers and/or pain affects daily activities | Disabling pain |
| Headache | not affect daily activities and not need treatment | Transient, slightly affect daily activities and may require treatment or intervention | Seriously affect daily activities and need treatment or intervention | Intractable, requiring emergency or hospitalization |
| Coughing | Transient, without treatment | Persistent cough, effective treatment | Paroxysmal cough, treatment uncontrollable | Emergency or hospitalization |
| Dyspnea | Dyspnea during exercise | Dyspnea in normal activity | Difficulty breathing at rest | Difficulty breathing, needing oxygen therapy, hospitalization or assisted breathing |
| Pruritus at non-vaccination site (No skin damage) | Slight itching not or slightly affects daily life | affects daily life | Itching makes it impossible for daily life | NA |
| Abnormal Skin Mucosa | Erythema/pruritis/colour | Diffuse rash/maculopapular rash/dryness/desquamation | Blister/exudation/desquamation/ulcer | dermatitis involves mucosa, or erythema multiforme, or is suspected of Stevens-Johnson syndrome |
| Acute Allergic Reaction * * | Local urticaria (blister) without treatment | Local urticaria, requiring treatment or mild angioedema, requiring no treatment | Extensive urticaria or angioedema requiring treatment or mild bronchospasm | Anaphylactic shock or life-threatening bronchospasm or laryngeal oedema |
| Tiredness/Fatigue | Not affect daily activities | Affect daily activities | Seriously affect daily activities and cannot work | Emergency or hospitalization |

Note:

CONFIDENTIAL

* Axillary temperature is usually used in China, tympanic temperature/temporal artery temperature = axillary temperature +0.5 ℃. When persistent high fever occurs, the cause of high fever should be determined as soon as possible;
** For constipation, attention should be paid to the changes before and after vaccination;
When judging severity, it can be judged as a next grade adverse event only when it meets the grading criteria of the previous one.

For clinical abnormalities not covered in the above table, the severity grading evaluation of adverse events should be carried out according to the following criteria.

| Grade 1 | Grade 2 | Grade 3 | Grade 4 | Grade 5 |
|---|---|---|---|---|
| Mild: Short-term (< 48h) or slight discomfort, which does not affect activities and does not need treatment | Moderate: Mild or moderate activity restriction, which may require medical treatment, no or only mild treatment | Severe: obvious activity Restricted, need to see a doctor and receive treatment, and may need to be hospitalized | Critical: It may be life-threatening, severely limited in activities, and needs monitoring and treatment | Death |

#### 4.5.3 Relationship between Adverse Events and Investigational Vaccines

**Impossible:** Adverse events occur due to other factors, and there is sufficient evidence to prove that adverse reactions/events are caused by other reasons and unrelated to d vaccination.

**Unlikely:** The occurrence of adverse events may be caused by other factors, such as the clinical status of the participant, other treatments or accompanying medication, which are inconsistent with the known adverse reactions of vaccination.

**Possible:** Adverse events are consistent with known investigational vaccine information, have a reasonable temporal sequence with vaccination, and/or have occurred for vaccination. There is also a causal relationship with the investigational vaccine, but it may also be related to other factors.

**Likely:** Adverse events are consistent with the known investigational vaccine information and have a causal relationship with the investigational vaccine, and cannot be explained by other factors, such as the clinical status of the participant, other treatments or concomitant medication.

CONFIDENTIAL

**Definite:** Adverse events are consistent with the known investigational vaccine information and have a causal relationship with the investigational vaccine, and this relationship cannot be explained by other factors, such as the clinical status of the participant, other treatments or accompanying drugs. In addition, adverse events occurred repeatedly when the participants used the investigational vaccine again.

#### 4.5.4 Adverse Event of Special Interest

According to the CIOMS VII definition, AESI refers to a class of scientific and medical events (including serious or non-serious) related to investigational vaccines or projects, which require investigators to continuously monitor and quickly communicate with the Sponsor. These events need further study to understand their characteristics; Depending on the nature of the event, the Sponsor also needs to communicate quickly with other relevant parties (such as regulatory authorities). The follow-up period of AESI is 12 months after the full course of immunization. AESI related to this vaccine include:

- Nervous system diseases (generalized convulsions, Guillain-Barre syndrome, acute disseminated encephalomyelitis, aseptic meningitis, general convulsions, peripheral facial paralysis, and loss of smell/ taste)
- Immune system diseases (vaccine-related respiratory diseases increase, anaphylactic shock, and multisystem inflammatory syndrome in children)
- Respiratory diseases (acute respiratory distress syndrome)
- Cardiac system diseases (acute cardiovascular injury including myocarditis, pericarditis, arrhythmia, heart failure, myocardial infarction)
- Blood system damage (thrombocytopenia, coagulation dysfunction including coagulation diseases, thrombosis, thromboembolism, internal/external bleeding, and stroke)
- Kidney system diseases (acute kidney injury)
- Liver system diseases (acute liver injury)
- Skin system diseases (chilblain-like lesions, single organ skin vasculitis, erythema multiforme, and alopecia).

### 4.6 Randomization and Blinding

CONFIDENTIAL

### 4.6.1 Randomization

A stratified block randomization method will be employed in the study. Interactive Web Response System (IWRS) will be used in participant randomization and vaccine allocation.

The randomization statistician will produce a participant randomization list and vaccine randomization list by SAS 9.4 (or later version), which will be imported into IWRS by the system engineer. After one participant is screened successfully, the investigator at each centre will log onto IWRS to get the randomization number of the participant. Before vaccination, the investigator will receive the vaccine number from IWRS and inoculate vaccines accordingly. If the vaccine is damaged, the investigator could acquire a new vaccine number from IWRS and inoculate the vaccine with a new vaccine number.

### 4.6.2 Blinding

Before the start of the study, the staff from Sponsor who will not participate in the clinical trial will blind the vaccines to be used in the study together with the unblinded randomization statistician. The vaccine label will be stuck on the specified position of each vaccine according to the vaccine randomization list. The randomization statistician will supervise and guide the process of blinding. After blinding is completed, the vaccine randomization list will be sealed by the unblind randomization statistician. The whole blinding process will be recorded and written in a document named blinding records, which is one of the important documents of this trial. The blinding personnel cannot participate in other related works of the trial, and they are not allowed to disclose the blind code to any personnel who are participating in the trial.

The participant randomization list and vaccine randomization list will be sealed, including the blind code composed of parameters like the code corresponding group assignment, the seed number generating random codes, etc. The blind code will be well sealed and saved until the database is locked.

### 4.6.3 Emergency Unblinding

In an emergency, when the investigator thinks that the group assignment information of one participant is beneficial for the handling of adverse events, it will be broken through the

CONFIDENTIAL

Emergency Unblinding Module in IWRS. The investigators need to discuss with the Sponsor and both shall reach an agreement before performing emergency unblinding.

# 5. SCREENING AND WITHDRAWAL OF PARTICIPANTS

The participants must fulfil all eligibility criteria to be involved in this study. No exemptions from any in-/exclusion criteria will be allowed. If any protocol violations in enrollment are retrospectively detected for an already randomized participant, the investigator and Sponsor must determine immediately the safety of the participant in the subsequent study.

## 5.1 Participant Population

### 5.1.1 Inclusion Criteria

Individuals are eligible to participate in this study if all of the following criteria apply:

- Age: ≥18 years;
- Judged by the investigator that the health condition is well after inquiry and physical examination;
- Vaccinated with 2 or 3 doses of inactivated COVID-19 vaccine (Vero Cell vaccine) ≥6 months prior to study screening and according to product insert;
- Female participants who are not pregnant or nursing or at the time of enrolment (confirmed via negative urine pregnancy test), and do not have plans to become pregnant within the first 6 months after enrollment. Effective contraceptive measures have been taken within 2 weeks before study inclusion and initiation;
- Be able and willing to provide written informed consent to participate in the study and complete all study requirements according to the study protocol.

### 5.1.2 Exclusion Criteria

Individuals are not eligible to participate in this study and are therefore excluded from study participation if one or more of the following criteria apply:

- COVID-19 infection positive patients (including suspected or asymptomatic cases);
- Have a history of SARS and MERS infection;

CONFIDENTIAL

- Have been vaccinated by any COVID-19 Vaccines other than Vero Cell vaccine
- Have an axillary temperature ≥37.3℃ (forehead temperature ≥37.8℃);
- Have had previous allergic reactions to vaccination (such as acute allergic reactions, urticaria, eczema, dyspnea, angioneurotic oedema or abdominal pain) or allergy to known components of COVID-19 vaccine;
- History of thrombocytopenia or other coagulation disorders;
- Patients with known immunological impairment or immunocompromised.
- Received whole blood, blood products, plasma and/or immunoglobulin therapy within 3 month before study enrollment
- Have known or suspected severe illness such as respiratory illness, acute infection or active attacks of chronic illness, liver and kidney disease, severe diabetes mellitus, malignant tumour, infectious or allergic skin disease, human immunodeficiency virus (HIV) infection (test report available);
- Diagnosed with serious cardiovascular diseases such as cardiopulmonary failure, drug-uncontrolled hypertension (Systolic blood pressure ≥160 mmHg and/or diastolic blood pressure ≥ 95 mmHg).
- Received live attenuated vaccines within 1 month before study enrollment;
- Received inactivated vaccines within 14 days before study enrollment;
- Received other investigational drugs within 6 months before study enrollment;

Other vaccination-related contraindications considered by investigators.

### 5.1.3 Criteria for Early Withdrawal from the Trial

Early withdrawal means that the participant fails to complete the booster vaccination and blood collection procedures according to the clinical study protocol and permanently discontinues study participation.

When any of the following conditions occur, the participant will be withdrawn from the study.

- The participant requests to withdraw from the clinical trial;
- Intolerable adverse events, whether related to the investigational drug or not;
- The health status of the participant does not allow them to continue to participate in this trial;
- The participant is vaccinated with other clinical investigational vaccines during the study

CONFIDENTIAL

period;

- Any other reason that investigator considers.

### 5.1.4 Criteria for Clinical Trial Suspension and Termination

In case of any of the following circumstances, the clinical trial shall be suspended or terminated, and the investigator, the Sponsor and the Ethics Committee shall jointly hold a meeting to decide whether to terminate the clinical trial early:

- The number of participants with adverse reactions of severity rated Grade 3 or above exceeds 15% of the participants study vaccinated in this subgroup;
- One case of Grade 4 adverse reaction related to vaccination or a suspected unexpected serious adverse reaction (SUSAR) has occurred.

In case of any of the following circumstances, the clinical trial shall be terminated:

- The Sponsor found that the vaccine has a potential safety hazard, or the trial has quality-related problems and requires the complete termination of the study.
- The Ethics Committee requests to termination of the trial because of the ethical violation in the study.
- The administrative authority requests termination of the trial.

# 6. PRODUCT INTRODUCTION

## 6.1 Description and Characteristics of the Investigational Products

Recombinant COVID-19 Vaccine (NVSI-06-09) is a recombinant protein expressed by recombinant Chinese hamster ovary (CHO) cells and designed based on the receptor-binding domain (RBD) of S protein of SARS-CoV-2. Engineered cells were cultured, harvested and purified by a series of processes, and then added with aluminum hydroxide adjuvant.

This product is a universal recombinant COVID-19 vaccine against major variants such as Omicron. It is developed through in-depth research on virus structure, mutation patterns and mutation sites, especially based on the analysis of mutations at Omicron's receptor-binding domain. On the premise of ensuring safety, it is more universal, effective and durable than the existing

CONFIDENTIAL

recombinant vaccines, especially has significant protection potential against variants including Omicron and Delta, it is believed that this vaccine will play a key role in epidemic prevention and control.

This product is a sterile formulation, packed in a vial with a specification of 0.5 ml/dose/vial. After shaking, it is an opalescent suspension, which can be layered due to precipitation and easily shaken away without lumps.

The main active ingredient of this product is the recombinant protein formed by the receptor-binding region of SARS-CoV-2 spike protein. Excipients include sodium chloride, aluminum hydroxide adjuvant, histidine, etc. Each dose contains 20μg of recombinant protein, 4.38 mg of sodium chloride, 0.39 mg of histidine, 0.30 mg of aluminum and water for injection. Used to prevent diseases caused by SARS-CoV-2.

#### 6.1.1 Investigational Vaccine

**Investigational Vaccine**: Recombinant COVID-19 Vaccine (NVSI-06-09)
Manufacturers: Sinopharm CNBG National Vaccine and Serum Institute (NVSI),
Lanzhou Institute of Biological Products Co., Ltd. (LIBP),
Beijing Institute of Biological Products Co., Ltd. (BIBP),
Specifications: 0.5 ml/vial per human use, which contains 20ug of SARS-CoV-2 antigen.
Storage Condition: 2-8 ℃.
Batch Number:
Expiry Date:

**Control vaccine:** Inactivated COVID-19 Vaccine (Vero cell)
Manufacturer: Beijing Institute of Biological Products Co., Ltd.
Specifications: 0.5 ml/vial per human use, which contains 6.5U of inactivated SARS-CoV-2 antigen.
Storage conditions: 2-8 ℃
Batch Number:

CONFIDENTIAL

Expiry Date:

### 6.1.2 Production Process

The Recombinant COVID-19 Vaccine (NVSI-06-09) developed in this project takes CHO cells as the express system and SARS-CoV-2 spike protein as the target, applying genetic engineering technology to construct and screen stable cell lines with recombinant expression protein. After culture, harvesting and a series of purification processes and adding aluminum hydroxide adjuvant, the vaccine is finished after filling.

## 6.2 Vaccine Packaging and Labeling

All investigational vaccines are packaged in doses in boxes with the same appearance and only marked by the vaccine number. Each study ID will be matched with a unique vaccine number, the format of the vaccine number is AE+4-digit Arabic number. See the following label for detailed information.

### 6.2.1 Vaccine Labeling

1. Each dose of vaccine is packaged separately and the vaccine number is stuck onto.
2. After the vaccine is put into use, the initials of the participant and the vaccination date shall be filled on the outer package.

**Clinical Trial Labeling Illustration**

Outer Packaging Box Label

| |
|---|
| **Recombinant COIVD-19 Vaccine (NVSI-06-09)** |
| **For Clinical Research Use Only** |
| **Vaccine Number： AExxxx** |
| **Initials： Vaccination Date： YY MM DD** |
| **Storage at 2-8℃, 0.5mL/dose** |
| **Batch Number：** |

Vaccine Label:

CONFIDENTIAL

| |
|---|
| **Recombinant COVID-19 Vaccine (NVSI-06-09)**<br>**（For Clinical Research Use Only）**<br>Vaccine Number：AExxxx<br><br>Batch Number：XXXX |

## 6.3 Vaccine Storage and Transportation

Vaccines should be stored and transported at 2 ~ 8 ℃ away from light to prevent freezing. The temperature during vaccine transportation and storage shall be dynamically monitored and recorded. If the storage and transportation temperature conditions exceed the specified range, the on-site investigators should immediately contact the personnel of the responsible clinical institutions and the Sponsors to decide whether the vaccine can be used.

The management of receiving, keeping, formulation, recycling, returning/destroying of the investigational vaccine shall conform to the requirements of relevant laws and regulations.

### 6.3.1 Vaccine Transportation

The whole process of vaccine management should meet the cold chain requirements, and there should be vaccine transportation and storage conditions that meet the requirements of the protocol. During the vaccine transportation, there should be a delivery note and temperature monitoring. Upon arrival, the packaging condition and unpacking temperature should be recorded. After the recipient receives the vaccines, the delivery note should be signed, faxed, or copied to the sender. Both parties should keep the delivery note properly.

### 6.3.2 Vaccine Storage and Distribution

The investigational vaccines should be kept in a separate area, locked in a dedicated cabinet and managed by dedicated personnel. Vaccine recipients must verify and record the batch number, expiry date and delivery status of vaccines, create worksheets for vaccine handover, registration, use and recycle, fill in as required, and keep them in the working logs. The trial vaccine shall not be used in the non-clinical trial population.

CONFIDENTIAL

### 6.3.3 Vaccine Records

***Vaccine handover records****:* The Sponsor will provide investigational vaccines, control vaccines (placebo) and vaccine handover sheet, and the investigator will verify the name, batch number and quantity of the vaccine upon receiving.

***Vaccine registration and use records****:* the investigator shall establish vaccine registration and use records and distribute investigational vaccine and control vaccines according to the number of people in the observation groups.

***Vaccine recycle record****:* The abandoned, expired and remaining vaccines in this trial are returned to the Sponsor. Sponsor receives vaccines and verifies the batch number and quantity of vaccines, fills in the vaccine handover form, and makes relevant records, which are signed by the vaccine manager and Sponsor representatives.

### 6.4 Administration and Immunization Schedule

***Route of Administration***: lateral deltoid muscle of either upper arm, intramuscular injection.

***Vaccination Procedure***: Among the population who had completed two/three doses of inactivated COVID-19 vaccines according to the 0 and 21 days (+7 days) immunization schedule, one dose of Recombinant COVID-19 Vaccine (NVSI-06-09) or inactivated COVID-19 vaccine (Vero cell) will be inoculated at≥ 6 months for booster immunization.

## 7. STUDY WORKFLOW AND VISITING METHOD

### 7.1 Schedule of Visits

Table 5 lists the assessment items and procedures according to the phase of the clinical studies and visits.

### 7.2 Study Procedure

CONFIDENTIAL

### 7.2.1 Screening Period/Baseline Period and Vaccination Procedure (D0)

The first visit was conducted according to the following procedure and evaluation processes.

**Recruitment:**

The investigators will jointly publicize this clinical trial recruitment notice to qualified volunteers/guardians, examined, and approved by the Ethics Committee, with local medical staff, and recruit and register candidate participants based on the principle of voluntary participation. During the study, the recruitment should be adjusted in real-time progress, to ensure that the gender and age of the participants are relatively balanced.

**Informed Consent:**

Informed consent refers to the voluntary participation of participants in clinical studies. Before the commencement of any test-related procedures (any genetics-related sample collection, testing, etc.), written informed consent must be obtained from the participant.

After arriving at the research site, the participants first give informed consent. The investigators inform the participants of the informed consent form of this clinical trial orally and in writing, including study purpose, procedure, duration, risks and benefits, etc. Under the condition of voluntary participation, the participants and research doctors signed an informed consent form together. The informed consent form shall be made in duplicate, the guardian or principal of the volunteer shall keep a copy, and the original shall be kept at the study site.

Collect contact information (address, e-mail, landline, mobile phone numbers, etc., if applicable) of participants and their families.

**Physical Examination, Inclusion/Exclusion Criteria:**

Volunteers who signed the informed consent were given height, weight, body temperature, blood pressure (aged 18 and above), cardiopulmonary auscultation, skin examination. Within 10 minutes before the temperature test, the participants are forbidden to eat or drink anything hot or cold, or smoke.

According to the "inclusion criteria" and "exclusion criteria", the investigators inquire, understand,

CONFIDENTIAL

and check the vaccination records of the participants, that is, the past medical history, and judged whether the participants should be enrolled in this clinical trial according to the inquiry results.

**Study Number Assignment:**

Eligible participants are assigned unique study numbers in sequence. Once the study number is assigned, it cannot be reassigned to other participants.

**Blood Sample Collection before Vaccination:**

Women of childbearing age (from menarche to menopause) need to collect about 2ml urine samples for urine pregnancy tests before vaccination.

About 12.5 ml of blood samples are collected on an empty stomach for SARS-CoV-2 antibody detection after recruitment.

Nasopharyngeal swabs are collected for PCR detection to establish antibody baseline and PCR baseline for booster inoculation. After sample collection, the participants can enter the following process.

**Vaccination:**

Participants can be vaccinated after sample collection. Before vaccination, it is necessary to check the information of participants and the investigational vaccines, obtain vaccines with corresponding numbers and open the outer package. After the vaccine label and outer package label are checked, fill in the initials of the vaccinee's name and vaccination date on the label of the outer package.

Vaccination site is at the lateral deltoid muscle of the upper arm, in principle, the non-habitual arm of the participant.

During vaccination, the investigational vaccine and control vaccine should be kept at 2 ~ 8 ℃ (the temperature should be monitored and recorded every 1 hour), and it should take no more than 30 minutes from the time the vaccine is taken out of the heat preservation container to the

CONFIDENTIAL

completion of vaccination.

During vaccination, in case of abnormal colour, damage, insufficient loading or precipitation, it is necessary to stop vaccination immediately, report to the Sponsor, auditor and on-site person in charge, discard the original vaccine according to the procedure after confirming the damage of the vaccine, and obtain a new vaccine number in the system by the distribution principle of the central randomized system after approval. Record the injection site (right arm or left arm) and vaccination information.

**On-site Medical Observation:**

Observe the immediate adverse reactions 30 minutes after vaccination. Doctors explain on-site adverse reaction judgment, measurement methods, recording methods, precautions, reporting methods, etc. Diary cards, rulers and thermometers are distributed and train participants how to use thermometers correctly, observe adverse events and fill in diary cards within 0-7 days after vaccination, and inform them of the time to make an appointment to return the diary cards (return the completed diary cards on 28th day after vaccination).

Ask the participants to try to take temperature measurements at the same time every day within 7 days after vaccination. Before leaving the study site, the vaccination site and body temperature of the participants should be checked, and the observed adverse events should be evaluated and recorded in the electronic system.

Inform the participants that they should take the initiative to provide any adverse reactions and accompanying medication. If there is any reaction after vaccination, it is necessary to inform the study centre in time. PI or qualified SI will evaluate them, provide the participants with the correct response plan, and ask them to return to the clinic for immediate evaluation if necessary.

## 7.3 Safety Observation and Follow-up

### 7.3.1 Safety Observation

1. Observe the site for 30 minutes after vaccination to collect local and systemic adverse events.
2. Telephone follow-up once in 6-24 hours after vaccination, within 1-3 days and 4-7 days

CONFIDENTIAL



CONFIDENTIAL

respectively, the participants were followed-up by telephone and guided to observe the adverse events correctly and fill in the diary card.

3. On the 14th day after vaccination, the investigator reviews the filling of the diary card during this period and re-collect the diary card.
4. On the 8th -28th day after vaccination, the investigator uses telephone follow-up once a week, combined with an active report of the participants to monitor the safety.
5. When it is known that the participant has adverse reactions/events of Grade 3 or above, a face-to-face visit should take place within 24 hours.
6. SAEs and AESIs are observed from the first dose to 12 months after the full course of vaccination via monthly telephone follow-up calls and self-reports from the participants.
7. Participants should contact the doctors on informed consent form/diary card immediately if developed any COVID-19 like symptoms.

### 7.3.2 Safety Information Record

After vaccination, all participants should complete the diary card. Investigators will ask participants about adverse events during on-site visits and telephone follow-up after vaccination (except for scheduled/unscheduled visits to the research centre). If the participant has persistent local or systemic reaction within 0-28 days after vaccination, it should be continuously recorded and reexamined until the symptoms are relieved.

## 7.4 Laboratory Testing

Collect laboratory samples (including blood samples) after physical examination, and complete sample collection before vaccination. The sampling time and arrangement of laboratory testing samples during the study period are shown in the Schedule of Visits, and the samples are tested and analyzed by the central and/or local laboratories.

**Pregnancy Sample Collection:**

Pregnancy tests (via urine/blood samples) must be conducted for all female participants of childbearing age. Test results will be used for screening.

**Nucleic Acid Sample Collection:**

CONFIDENTIAL

All participants need to collect nasopharyngeal swabs for RT-PCR detection at the same time of recruitment, and the test results will be used for subsequent analysis.

**Blood Sample Collection before Immunization (D0):**

After enrolled, all participants will be collected about 12.5 ml of venous blood before vaccination, separate the serum within 24 hours, aliquot into multiple test tubes (each tube is not less than 0.5 ml), and store at -20℃ or below for antibody testing.

**Blood Sample Collection after Immunization**

About 12.5 ml of venous blood was collected from all participants at 0 day, 14 days, 28 days, 3 months, 6 months, 9 months and 12 months after immunization, and the serum was separated within 24 hours and aliquoted into 2 tubes (each tube is not less than 0.5 ml), which were stored at -20℃ or below for neutralizing antibody testing.

A total of 20 participants from each study group will be sequentially invited to give 5 additional venous blood samples (12ml per participant per sample). Peripheral blood mononuclear cells (PBMC) are separated, and the activation of specific T cells are detected by enzyme-linked immunospot (ELISPOT) and flow cytometry. T-cell receptor (TCR) characteristics of specific T cells and B-cell antigen receptor (BCR) characteristics of B cells are analyzed using high-throughput single-cell sequencing to analyze the type and proportion of immune cells in PBMC, as well as the expression profile of immune-related genes in each subgroup of cells.

**Options for cellular immune testing in clinical trials:**

| Method | Antigen stimulation | Primary targets | Additional targets |
|---|---|---|---|
| Intracellular cytokine stain (ICS) (flow cytometry) | Required | IFN-γ$^+$ CD4$^+$ T cells, IFN-γ$^+$ CD8$^+$ T cells | IL-2$^+$/TNF-α$^+$ CD4$^+$ T cells, IL-2$^+$/TNF-α$^+$ CD8$^+$ T cells, IL-4$^+$/IL-5$^+$ CD4$^+$ T cells |
| ELISPOT | Required | IFN-γ | IL-2, TNF-α |

CONFIDENTIAL

**Blood Sample Collection of Cases (Suspected or Confirmed)**

After the participants develop typical symptoms of SARS-CoV-2 infection, about 4 ml of venous blood is collected for antibody detection, and the serum is separated and aliquoted into 2 tubes (each tube is not less than 0.5 ml) within 1-3 days. In addition, about 10ml venous blood is collected, and cytokines, cellular immunity and other testing items are carried out according to local medical conditions, laboratory clinical level. Serum is stored at -20℃ or below (antibody detection is carried out for participants with confirmed and suspected cases).

**Blood Sample Numbering Rule**

Before the booster dose: study number – 0

14 days after vaccination (D14+10 days) study number – 1

28 days after vaccination (D28 + 10 days) study number -2

3 months after vaccination (D90 + 30 days) study number-3

6 months after vaccination (D180 + 30 days) study number -4

9 months after vaccination (D270 + 30 days) study number -5

12 months after vaccination (D360 + 30 days) study number-6

Suspected and confirmed cases are coded in P1, P2, P3…according to the order of blood collection.

CONFIDENTIAL

## Table 4 Schedule of Events

| Visit number | V1 | V2 | V3 | V4 | V5 | V6 | V7 | V8 | V9 | V10 | V11 | V12 | V13 |
|---|---|---|---|---|---|---|---|---|---|---|---|---|---|
| Date (Security Mode) | D0 👓 | D3 ☎ | D7 ☎ | D14 👓 | D21 ☎ | D28 👓 | 2M after V1 ☎ | 3M after V1 👓 | 4M after V1 ☎ | 5M after V1 ☎ | 6M after V1 👓 | 9M after V1 👓 | 12M after V1 👓 |
| Visit (window +/-days) The subsequent visit is postponed accordingly | 0d | -2d | -3d | + 10d | +7d | + 10d | +10d | + 30d | +10d | +10d | + 30d | + 30d | + 30d |
| **SCREENING/BASELINE** | | | | | | | | | | | | | |
| Study informed consent [1] | ✓ | | | | | | | | | | | | |
| Inclusion/Exclusion criteria evaluation | ✓ | | | | | | | | | | | | |
| Demographics | ✓ | | | | | | | | | | | | |
| Medication history | ✓ | | | | | | | | | | | | |
| Contraception [2] | ✓ | | | | | | | | | | | | |
| IWRS Registration | ✓ | | | | | | | | | | | | |
| **INTERVENTION** | | | | | | | | | | | | | |
| Vaccination | ✓ | | | | | | | | | | | | |
| **SAFETY ASSESSMENT** | | | | | | | | | | | | | |
| Vital signs (blood pressure, body temperature, heart rate) | ✓ | | | | | | | | | | | | |
| Height, Weight | ✓ | | | | | | | | | | | | |
| Physical Examination | ✓ | | | | | | | | | | | | |
| Onsite observation after vaccination [3] | ✓ | | | | | | | | | | | | |
| Telephone follow-up after vaccination | ✓ | ✓ | ✓ | | ✓ | | ✓ | ✓ | ✓ | ✓ | ✓ | | |
| Concomitant Medication | ✓ | ✓ | ✓ | ✓ | ✓ | ✓ | ✓ | ✓ | ✓ | ✓ | ✓ | | |
| Adverse events | ✓ | | | | | | | | | | | | |
| SAE | ✓ | | | | | | | | | | | | |
| Diary Card [4] | ✓ | | | ✓ | | | | | | | | | |
| **LABORATORY TESTING** | | | | | | | | | | | | | |
| COVID-19 PCR[5] | | ✓ | | | | | | | | | | | |
| Neutralizing Antibody/IgG Test | ✓ | | | ✓ | | ✓ | | ✓ | | | ✓ | ✓ | ✓ |
| Cellular Immunity Testing[6] | ✓ | | ✓ | ✓ | | ✓ | | ✓ | | | ✓ | | |

CONFIDENTIAL

1. Consent process completed, and form signed before any study-related procedures are conducted.
2. Urine/blood pregnancy test must be completed within 24 hours prior to vaccination for women of childbearing potential, and only negative test results are eligible for enrollment.
3. All participants should be observed onsite for at least 30 minutes after vaccination.
4. Paper diary cards will be used for safety information records and collected from the participant on Visit 4 (D14).
5. PCR testing could be performed any time during the study when the participant developed any suspected symptoms.
6. Collect blood samples from the first 20 participants in each study group for cellular immunity testing.

CONFIDENTIAL

CONFIDENTIAL

### 7.5 COVID-19 Case Monitoring

This clinical trial adopts a monitoring modality of positive COVID-19 cases with passive monitoring as the main approach and active monitoring as a supplement. After the participants receives the vaccination, the monitoring of positive COVID-19 cases will be initiated until the end of the study, and relevant information will be collected.

**A. Passive Monitoring**

Passive monitoring has two situations where participants spontaneously report and when the participants develop any symptoms, they actively go to a hospital to seek for medical care.

1. After the participants develop symptoms, they actively report to the investigator through the hotline or email;
2. When the participants visit a hospital after developing any symptoms, the participants are monitored through hospital system;
   - When the participant has fever and/or respiratory symptoms that meet the definition of a suspected case, the participant should inform the investigator and go to the designated place for PCR testing;
   - If the participant visits a hospital for treatment, the investigator should arrange to collect nasopharyngeal swabs for PCR testing every 2-3 days; determine the clinical classification based on the severity and duration of symptoms, and isolate the mild and moderate cases; arrange hospitalization for severe and critical cases;
   - For non-hospitalized patients, the participants will be followed up by remote teleconsultation and re-tested by PCR testing every 3 days to monitor and document the progress of the illness, medication status, symptom severity and other related information. If the participant's condition deteriorates, he/she needs to be transferred to the hospital for timely treatment;
   - For hospitalized patients, continue to pay attention to the development of the disease, and conduct necessary testing and examination such as blood routine, blood chemistry, imaging examinations as well as other medical treatment. Closely monitor and collect evidence related to the participant's diagnosis and treatment, and record the progress of the disease, medication, and the severity of symptoms

CONFIDENTIAL

until the outcome;

- Collect the PCR result, a confirmed case is determined based on the positive result;
- During the convalescent period (defined as 3-4 weeks after the first positive PCR result) for COVID-19 infection positive participant's only, a nasopharyngeal swab is collected for PCR re-test; if the PCR test result is still positive, repeated PCR testing needs to be performed once per week until the PCR result turns negative.
- Neutralizing antibody testing will be conducted according to Investigator clinical judgement.

**Endpoint assessment of the cases**

Establish an independent case Endpoint Assessment or Adjudication Committee (EAC) composed of 3 or more local medical experts to make a final diagnosis for each case. When a case meets the etiological indicators, the diagnosis and treatment progress document, case investigation document, serology, cytokines and other lab reports will be submitted to EAC, the case can be determined as a confirmed case of COVID-19 (clinical endpoint) after a blinded review of the EAC experts.

CONFIDENTIAL

**Table 6 Testing for PCR positive cases in acute and convalescent phase**

| | Clinical Classification | Acute Phase | Convalescent Phase |
|---|---|---|---|
| PCR-positive cases | Non-hospitalized-Asymptomatic / Mild | SARS-CoV-2 PCR testing | SARS-CoV-2 PCR testing |
| | | IgM, IgG, Neutralizing antibody | IgM, IgG, Neutralizing antibody |
| | | Pathogenic Differential diagnosis （if applicable） | |
| | Hospitalized-Moderate/Severe / Critical | SARS-CoV-2 PCR testing | SARS-CoV-2 PCR testing |
| | | IgM, IgG, Neutralizing antibody | IgM, IgG, Neutralizing antibody |
| | | CBC with differential (WBC, lymphocytes…) | |
| | | Blood chemistry (liver enzymes, eGFR, LDH, CRP) | |
| | | Chest CT scan/CXR | |
| | | Pathogenic Differential diagnosis （if applicable） | |
| | | Cytokines : IL-2, IL-6, IL-4, TNF-α, IFN-γ and other cytokines （if applicable） | |

CONFIDENTIAL

## 7.6 Concomitant Medication

Within 0-28 days, actively follow up and observe the local and systemic adverse events of the participants and record them on the vaccination diary card. At the same time, it is necessary to record the drug used of the participants on the vaccination diary card.

**Medication Allowed:** During the clinical trial, if the participant has adverse events, necessary medication and medical treatment should be allowed.

**Vaccines Allowed:** None COVID-19 vaccines are allowed during the trial, which should be carried out 15 days after the trial vaccination. Emergency vaccination, such as rabies vaccine and tetanus vaccine, is not restricted.

CONFIDENTIAL

**Medication Record:** To understand the influence of drug use during the trial on vaccine safety and immunogenicity, and to collect adverse events that may be related to vaccination without omission, investigators should instruct the guardians of the participants to record the medical treatment and medication in diary cards as much as possible and assist in collecting the hospitalization medication records of the participants during the observation period. When SAE occurs, copies of corresponding medical records and medication records should be collected and kept. The all study additional drugs need to be transcribed into the electronic case report form (eCRF), such as but not limited to:

1. Hormone/steroid drugs and other immunosuppressants.
2. Antiallergic Drugs
3. Antipyretics/analgesics/NSAIDs
4. Prophylactic Vaccines
5. Therapeutical Medical Products
6. Antibiotics
7. Antiviral Drugs
8. Chinese Patent Drugs

CONFIDENTIAL

## 7.7 Criteria for Suspension or Early Termination

- The number of participants with adverse reactions of severity Grade 3 or above exceeds 15% of the participants vaccinated in this subgroup;
- One case of Grade 4 adverse reaction related to vaccination or SUSAR;

**In case of any of the following circumstances, the clinical trial shall be terminated:**

- Sponsor has found that the vaccine has potential safety hazards or the trial has quality related problems, and requiring the complete termination of the study.
- The Ethics Committee requests the termination of the trial because of the violation of ethics in the trial.
- The administrative authority requests the terminatation of the trial

CONFIDENTIAL

## 7.8 Protocol Violation and Deviation

### 7.8.1 Protocol Violations

The list of protocol violations is as follows (including but not limited to):

- Fail to obtain participant's informed consent;
- Participants who did not meet the inclusion criteria or met the exclusion criteria were enrolled in the study;
- Participants received wrong research interventions (such as incorrect vaccinations);
- Fail to report serious adverse events (SAE) within specified time;
- Received other investigational vaccines during the study period.

### 7.8.2 Protocol Deviations

The list of protocol deviations is as follows (including but not limited to):

- Not receive the investigational vaccine within the window period;
- Not collect blood within the window period;
- The interval between vaccination and other vaccines is not long enough, except for emergency vaccination such as rabies.

CONFIDENTIAL

## 7.9 Study Duration

Early Participant Recruitment: 1 month;

Booster Vaccination + immune persistence observation: about 13 months

Sample Testing: approximately 1 month

Complete the Summary Report: about 1 month

## 7.10 Site Close

All participants completed long-term safety observation for 12 months after vaccination and blood collection for 12 months after immunization. Trial sites will be closed and immune persistence evaluation will be initiated only once the following activities have been completed:

1. Completed all study requirements according to the study protocol
2. Locked the database
3. Clarification of data by the statistician (blind audit)
4. Unblinded the necessary study staff to evaluate the safety, immunogenicity and

CONFIDENTIAL

vaccine efficacy.

# 8. SAFETY DEFINITION, REPORTING AND MONITORING

## 8.1 General Precautions

Before the start of the trial, the Sponsor shall strictly examine the trial site in accordance with GCP requirements, focusing on whether the environmental facilities of the trial site meet the requirements of "Vaccination Management Standards" and "Guiding Principles for Quality Management of Vaccine Clinical Trials". Ensure the first aid facilities and first aid equipment in the first aid room are complete and effective, and the first aid doctors have corresponding qualifications and capabilities. Emergency related personnel (emergency doctors, emergency nurses, ambulance drivers, etc.) are trained to be qualified and familiar with the transfer routes and procedures of the agreed hospital. They are on standby at the trial site during vaccination. The trial site shall be equipped with ambulances. The ambulance shall be parked in a fixed position to keep the vehicle in good condition and in an emergency and shall be under the command and transfer of the emergency response team at any time. During the vaccination period, the agreement hospital will make daily preparations for medical personnel, instruments and equipment, first aid drugs and first aid sites to ensure that the participants can receive timely treatment. The trial site shall formulate an emergency plan, stipulate personnel responsibilities, contact numbers, rescue routes and other measures to ensure timely handling of unexpected adverse events, and ensure effective contact between participants and investigators so that any adverse events can be reported and handled quickly.

CONFIDENTIAL

## 8.2 Risk Prevention Measures Related to COVID-19

**(1) Trial site consideration**

Investigators during recruitment should confirm whether there are COVID-19 infections or suspected cases in the same community by investigating or inquiring volunteers. Whether fever, dry cough, fatigue, nasal obstruction, runny nose, sore throat, myalgia, diarrhea, shortness of breath and dyspnea occurred recently.

**(2) Strengthen the management and personal protection of participants during the study period**

CONFIDENTIAL

Disinfection of each functional area of clinical research should be strictly done in the test site according to regulations, and windows should be opened and ventilated regularly. Strictly implement independent areas and special passages, and the recipients and their accompanying personnel wait in relative divisions to avoid contact with people other than research doctors and nurses; All functional areas and public places should be equipped with hand disinfectants and temperature measuring devices, and recipients and their accompanying personnel entering the clinical research site should be equipped with masks to disinfect their hands and measure their body temperature.

The trial site shall strictly disinfect each functional area of clinical research according to regulations, and regularly open windows for air-ventilation. Strict implementation of independent areas and special passages, recipients and their accompanying personnel waiting in relative zones, to avoid contact with research doctors, nurses and other personnel other than research; All functional areas and public places should be equipped with hand disinfectants and temperature measuring devices. Recipients entering the clinical research site and their accompanying personnel are required to be equipped with masks to disinfect their hands and measure their body temperature.

During the first dose of inoculation, the on-site investigators shall remind the participants to strengthen their own protection. If COVID-19 pandemic occurs locally, the participants shall be provided with necessary protective materials such as masks, alcohol, etc. in time, and close attention to the health status of the participants shall be drawn, especially the symptoms related to COVID-19.

**(3) Detection of COVID-19 RT-PCR**

Participants need to be tested for COVID-19 RT-PCR before first vaccination. Investigators need to regularly follow up and track the participants to identify whether they have developed fever, dry cough, fatigue and other symptoms recently. If the above symptoms exist, the participants will be collected samples for etiological detection of SARS-CoV-2 according to the prevention and protection policy for COVID-19 and based on the symptoms of the participants, or are advised to go to the hospital for blood testing and radiographic examination. If there are no symptoms such as fever, dry cough and fatigue, the participant

CONFIDENTIAL

should be home isolated according to the requirements of COVID-19 prevention and control protocol.

## 8.3 Handling and Reporting of Serious Adverse Events

Serious adverse events refer to adverse medical events such as disability or loss of function, the need for hospitalization or prolonged hospitalization of participants, and congenital abnormalities or birth defects.

Monitoring and reporting of adverse events in vaccine clinical trials are jointly completed by participants, serious adverse event investigators, trial sites and responsible institutions at different observation time points with different stages.

The Sponsor is the main body responsible for monitoring, evaluating and SAE reporting safety information of vaccine clinical trials. Designated person acts as the administrator for clinical trial safety information monitoring and SAE reporting, and work with investigators to establish SOPs for clinical trial safety information monitoring and SAE reporting, know well the latest status of the safety information of the whole clinical trial, and timely report updates to all clinical trial institutions/ investigators and regulatory authorities.

CONFIDENTIAL

If it is difficult to make a judgment on the correlation between SAE and vaccine or there is doubt about the judgment, when it is necessary to make a new judgment, the expert meeting shall make a judgment after argumentation.

**(1) On-site Treatment Measures**

Emergency plans for SAE treatment in clinical trials shall be established at the trial site, and all relevant personnel shall be trained. If the participants show serious adverse events, the investigators shall immediately take appropriate measures for the participants and record them. The investigators must take relevant approaches to know in time any clinically significant diseases/events related to vaccination. According to relevant national regulations, the participants should be received appropriate treatment in time in the designated hospitals.

Investigators at the trial site should follow up serious adverse events until the symptoms disappear or stabilize. The progress and outcome of all symptoms will be recorded in detail, and all drug treatments and medical treatments will be recorded at each follow-up.

CONFIDENTIAL

Investigators should truthfully record serious adverse events, which shall be evaluated and discussed in the final report after the test is completed or terminated.

During the whole observation process, if the participants suffer from physical injuries caused by serious adverse reactions related to vaccination, which is confirmed by the expert investigation team, the local insurance company will give corresponding compensation.

**(2) Reporting Procedures for Serious Adverse Events**

***Reporting procedures of investigators include***:

Any serious adverse event, whether related to the investigational vaccine, the investigator must submit the initial report of the "Serious Adverse Event Report Form" to the drug administration department, the Sponsor and the ethics committee by fax, e-mail or EDC system or personal delivery within 24 hours after learning about it. Subsequently, the follow-up report of the "Serious Adverse Event Report Form" shall be submitted regularly until the end of the event. All information is reported in the "Serious Adverse Event Report Form" in the form of written reports, including description of adverse reactions/events, onset time and type, duration, intensity, causal relationship with vaccination, results, treatment methods (symptomatic treatment) and other relevant clinical and laboratory data.When receiving the report of serious adverse events/reactions, the investigator shall, together with the Sponsor, comprehensively consider the duration, scope, intensity, outcome and the willingness of the participant to decide whether the participant should continue to participate in the study or withdraw from the study in advance.

CONFIDENTIAL

***Reporting procedure of Sponsors include:***

During the clinical trial of drugs, Sponsors need to quickly report unexpected and serious adverse reactions (SUSAR) that are definitely related to or suspicious of the tested drugs in the form of case-by-case safety reports according to the Standards and Procedures for Rapid Reporting of Safety Data during Clinical Trial of Drugs. If the researcher and the Sponsor cannot reach an agreement on the judgment of the causal relationship between adverse events and drugs, either party's judgment cannot exclude those related to the test drugs, and the Sponsor should also make a quick report.

CONFIDENTIAL

For SUSAR that is fatal or life-threatening, the Sponsor should report it as soon as possible after the first knowledge, but not more than 7 days, and report it within the following 8 days to improve the follow-up information (Note: the day when the Sponsor first learned it is the 0th day). For SUSAR that is not fatal or life-threatening, the Sponsor should report it as soon as possible after the first knowledge, but not more than 15 days. For other potential serious safety risk information, the Sponsor should also report to the national drug evaluation agency as soon as possible, and at the same time make medical and scientific judgment on each situation. After the first report, the Sponsor shall continue to track the serious adverse reactions and submit relevant new information or changes to the previous report in a timely manner in the form of a follow-up report. The reporting time limit shall be within 15 days from the date of obtaining the new information.

### 8.4. Outcome of Serious Adverse Events

CONFIDENTIAL

The outcomes of serious adverse events include:

1. Symptoms resolved (with sequelae);
2. Symptoms resolved (without sequelae);
3. Symptoms persist;
4. Require hospitalization;
5. Require medical intervention to prevent serious outcomes; and
6. Death.

## 9. COMPLETION OF CLINICAL TRIAL

After obtaining the immunogenicity test results 14 days after the full course of immunization, completing the safety follow-up for 28 days, ensuring the authenticity, integrity and accuracy of clinical trial data, the database is locked and unblinded to perform safety, immunogenicity and vaccine efficacy evaluation, and blood collections for 12 months immune persistence are completed, the study site will be closed.

CONFIDENTIAL

# 10. STATISTICAL CONSIDERATIONS

## 10.1 Sample Size Considerations

**Primary indicators:**

- The antibody GMT (Omicron) in the experimental group was superior to that in the control group 14 days after immunization.
- H0: δ ≤0.0212 vs. H1: δ >0.0212 (both are logarithmic scale, equivalent to GMT ratio 95%CI > 1.05)

**Calculating basis for sample size**: by referring to the relevant data of this product in ex-China clinical trials, 0.55 (logarithmic scale) is taken as the standard difference in antibody titer of recombinant COVID-19 vaccine (CHO cell). The probability of type I error α=0.025 (one-sided); power $1-\beta$ = 0.9; superiority margin Δ=0. 0212 (logarithmic scale, i.e. GMT test group/GMT control group ≥ 1.05); the test group and the control group are designed according to 1:1.

CONFIDENTIAL

| Test level (one sided) | Target | N of each group | Superiority margin | Actual level of difference between test group and control group | SD | Total N after considering 20% drop-out |
|---|---|---|---|---|---|---|
| **0.025** | **0.9** | **166** | **0.0212 (1.05 fold)** | **0.200( 1.6 fold)** | **0.55** | **398** |
| 0.025 | 0.8 | 139 | 0.1139 (1.3) | 0.30 （2 fold） | 0.55 | 348 |
| 0.025 | 0.85 | 158 | 0.1139 (1.3) | 0.30 | 0.55 | 396 |
| 0.025 | 0.9 | 185 | 0.1139 (1.3) | 0.30 | 0.55 | 462 |

CONFIDENTIAL

| | | | | | | |
|---|---|---|---|---|---|---|
| 0.025 | 0.8 | 309 | 0.176(1.5) | 0.30 | 0.55 | 774 |
| 0.025 | 0.85 | 354 | 0.176(1.5) | 0.30 | 0.55 | 886 |
| 0.025 | 0.9 | 414 | 0.176(1.5) | 0.30 | 0.55 | 1036 |
| 0.025 | 0.8 | 368 | 0.176(1.5) | 0.30 | 0.60 | 920 |
| 0.025 | 0.85 | 421 | 0.176(1.5) | 0.30 | 0.60 | 1054 |
| 0.025 | 0.9 | 493 | 0.176(1.5) | 0.30 | 0.60 | 1234 |
| 0.025 | 0.8 | 158 | 0.176(1.5) | 0.35 | 0.55 | 395 |
| 0.025 | 0.85 | 181 | 0.176(1.5) | 0.35 | 0.55 | 452 |
| 0.025 | 0.9 | 211 | 0.176(1.5) | 0.35 | 0.55 | 528 |
| 0.025 | 0.8 | 96 | 0.176(1.5) | 0.40 | 0.55 | 240 |
| 0.025 | 0.85 | 110 | 0.176(1.5) | 0.40 | 0.55 | 276 |
| 0.025 | 0.9 | 128 | 0.176(1.5) | 0.40 | 0.55 | 320 |

CONFIDENTIAL

**Secondary incidcators:**

The antibody GMT (prototype strain) of the experimental group was not inferior to that of the control group 14 days after immunization.

**Calculating basis for sample size:**

By referring to the relevant data of this product in ex-China clinical trials, 0.55 (logarithmic scale) is taken as the standard difference of the antibody titer of the recombinant COVID-19 vaccine (CHO cell). The probability of type I error α=0.025 (one-sided); power $1-\beta$ = 0.9; non-inferiority threshold Δ=-0.17609 (logarithmic scale, i.e., the lower bound of 95%

CONFIDENTIAL

confidence interval of GMT test group/GMT control group after immunization should be at least 0.67); The test group and the control group are designed according to 1:1.

| Test level (one sided) | Target | N of each group | Superiority margin | Actual level of difference between test group and control group | SD | Total N after considering 20% drop-out |
|---|---|---|---|---|---|---|
| **0.025** | **0.9** | **206** | **-0.17609 (2/3 fold)** | **0（1 fold）** | **0.55** | **516** |
| 0.025 | 0.9 | 262 | -0.17609 (2/3 fold) | -0.02（0.95 fold） | 0.55 | 656 |
| 0.025 | 0.9 | 344 | -0.17609 (2/3 fold) | -0.02（0.90 fold） | 0.55 | 860 |
| 0.025 | 0.9 | 566 | -0.17609 (2/3 fold) | -0.07（0.85 fold） | 0.55 | 1416 |

CONFIDENTIAL

Based on the above considerations and the drop-out rate, it is planned to encroll 516 participants.

## 10.2 Analysis Set

**Full Analysis Set (FAS)**: It includes all randomized participants who follow ITT principle, receive 1 dose of vaccine, and have valid immunogenicity data before the first dose.

**Per-Protocol Set (PPS):** It includs all participants who do not violate the inclusion criteria/exclusion criteria, receive 1 dose of vaccine, and have both valid pre- and post-vaccination immunogenicity data. PPS will be defined for immunogencity evaluations 14 and 28 days after booster vaccination, respectively.

**Immunogenicity Persistence Set (IPS):** It include the participants who are collected with blood samples at each time point for immunogencity persistence evaluation and with valid

CONFIDENTIAL

antibody data. Define the IPS for immunogenicity for 3, 6, 9 and 12 months after booster immunization respectively.

**Safety Set (SS):**

It includes all participants who receive one dose of booster vaccination. In immunogencity analysis, the participant who takes the vaccine erroneously will be included as randomized, and the safety data will be analyzed as treated.

## 10.3 Statistical Method

### 10.3.1 General Considerations

The measurement data is statistically described with mean, median, standard deviation, maximum and minimum values; Counting data or grade data are expressed in occurence and frequency. All statistical analysis will be performed by using SAS 9.4 or later version.

CONFIDENTIAL

### 10.3.2 Trial completion and demographic characteristics

The number of participants who are enrolled, completed and discontinued will be summarized in each group. The reason for discontinuation will be analyzed as well. The demographic characteristics will be summarized and described by treatment group.

### 10.3.3 Immunogenicity Analysis

Analysis of covariance (ANCOVA) model will be employed to analyze post-vacciantion GMT after logarithmic transformation. In the model, log-trandformed GMT after vacciantion will be included as dependent variable, pre-vaccination GMT after log-transformation is the covariate and group assignment and booster vaccination time are the fixed effect. The adjusted Least Squares Mean (LSMean) in each arm and difference between the two groups together with 95% confidence interval (CI) will be estimated from the model. After inverse log-transformation, caculate the adjusted GMT after vaccination and the LSMean of GMT ratio between the experimental group and control group together with 95% CI. The reverse cumulative distribution plot is to depict the pre- and post-vaccination antibody data in the experimental and control groups.

CONFIDENTIAL

### 10.3.4 Safety Evaluation

Adverse events and serious adverse events will be coded using the Medical Dictionary for Regulatory Activities (MedDRA), and summarized by System Organ Class (SOC) and Preferred Term (PT). AEs will be summarized by solicied and unsolicited AEs as well. The Treatment Emergent Adverse Events (TEAEs) will be analyzed here. AEs occurring before vaccination will be listed.

All TEAEs, vaccine-related TEAEs, vaccine-unrelated TEAEs, Grade ≥ 3 TEAEs and vaccine-related Grade ≥ 3 TEAEs will be summarized by group. The frequency, participants and percentage in each group will be reported. Fisher exact test will be used to compare the difference between the groups. The strat time and severity of TEAEs will be summaried. The vaccine-related TEAEs and vaccine-unrelated TEAEs will be listed.

All TESAE, vaccine-related TESAEs and vaccine-unrelated TESAEs will be summarized by group. The frequency, participants and percentage in each group will be reported. Fisher exact test will be used to compare the difference between the groups. All TESAEs will be listed.

CONFIDENTIAL

## 10.4 Subgroup Analysis

Not applicable.

## 10.5 Interim Analysis

No interim analysis is planned in this study.

## 10.6 Multiplicity

Not applicable.

## 10.7 Handling of Missing Data

In FAS immunogenicity analysis, LOCF (Last Observation Carried Forward) method will be used to impute the missing values in antibody data after vaccination. And other immunogencity endpoints will be derived from the imputed data. The missing data in safety and immunogencity persietence evaluation will be not imputed.

CONFIDENTIAL

# 11. ETHICAL AND REGULATORY MATTERS

This clinical trial will be conducted by the Sponsor, the Investigator, and delegated Investigator staff and Sub-investigator, in accordance with consensus ethics principles derived from international ethics guidelines, including the Declaration of Helsinki, and the ICH guidelines for Good Clinical Practice (GCP), all applicable laws, rules and regulations.

## 11.1. Ethics Committees and Regulatory Authorities

The ICH-GCP (E6 (R2)) guidelines require that approval must be obtained from an Independent Ethics Committee (IEC) prior to participation of human participants in research studies. Prior to the study onset, the protocol, informed consent, advertisements to be used for participant recruitment, and any other written information regarding this study to be provided to the participant or the participant's legal guardian must be approved by the IEC. Documentation of the relevant national IEC approval and of the IEC compliance with ICH Guideline E6 will be maintained by the site and will be available for review by the Sponsor or its designee or by the authorized members of regulatory agencies.

CONFIDENTIAL

The respective Ethics Committees approvals should be signed by the IEC Chairman or designee and must identify the IEC name and address, the clinical protocol by title and/or protocol number and the date approval and/or favorable opinion was granted. Also, a list of the EC members who attended the meeting when the Protocol/ Protocol amendment was discussed, including names and qualifications, needs to be provided by the EC to the investigator or the Sponsor/ his representative.

If any alterations, other than changes of administrative nature only, are made to the study protocol, a formal protocol amendment will be issued and submitted to relevant IEC for approval. The amendment will not be implemented until IEC approval, except in cases where immediate implementation is necessary to eliminate or prevent imminent hazard to the participants.

In the same way, approval from regulatory authorities (RA) should be granted before beginning the study. The investigator or the Sponsor representative must provide to the

CONFIDENTIAL

regulatory authorities the name and address of the EC along with a statement from the EC that it is organized according to GCP and the applicable laws and regulations. Amendments will be submitted to RA too for approval.

### 11.2. Responsibilities of the Sponsor

The Sponsor of this clinical trial is responsible to regulatory authorities for taking all reasonable steps to ensure the proper conduct of the clinical trial as regards ethics, clinical trial protocol compliance, and integrity and validity of the data recorded on the e-CRFs. Thus, the main duty of the monitoring team is to help the Investigator and the Sponsor maintain a high level of ethical, scientific, technical and regulatory quality in all aspects of the clinical trial.

At regular intervals during the clinical trial, the site will be contacted, through monitoring visits, letters or telephone calls, by a representative of the monitoring team to review study progress, Investigator and participant compliance with clinical trial protocol requirements and any emergent problems. These monitoring visits will include but not be limited to review of the following aspects: participant informed consent, participant recruitment and follow-up, SAE documentation and reporting, AE documentation, IP allocation, participant compliance with the instructions, IP accountability, concomitant therapy use, and quality of data.

### 11.3. Responsibilities of the Investigator

The Investigator is responsible for ensuring that the clinical study is performed in accordance with the protocol, the ethical principles that have their origin in the Declaration of Helsinki (version dated October 2013) as well as with the ICH Note for Guidance on Good Clinical Practice (ICH, E6, R2, 2016), relevant site SOPs and applicable regulatory requirements. These documents state that the informed consent of participants is an essential precondition for participation in the clinical study.

The Investigator is required to ensure compliance with all procedures required by the clinical trial protocol and with all study procedures provided by the Sponsor (including

CONFIDENTIAL

security rules). The Investigator agrees to provide reliable data and all information requested by the clinical trial protocol (with the help of the e-CRF, Discrepancy Resolution Form [DRF] or other appropriate instrument) in an accurate and legible manner according to the instructions provided and to ensure direct access to source documents by Sponsor representatives.

If any circuit includes transfer of data particular attention should be paid to the confidentiality of the participant's data to be transferred.

The Investigator may appoint such other individuals as he/she may deem appropriate as Sub-investigators to assist in the conduct of the clinical trial in accordance with the clinical trial protocol. All Sub-investigators shall be appointed and listed in a timely manner. The Sub-investigators will be supervised by and work under the responsibility of the Investigator. The Investigator will provide them with a copy of the clinical trial protocol and all necessary information.

CONFIDENTIAL

In 1998, the US Food and Drug Administration (FDA) introduced a regulation (21 CFR, Part 54) entitled “Financial Disclosure by Clinical Investigators.” For studies conducted in any country that could result in a product submission to the FDA for marketing approval and which contribute significantly to the demonstration of efficacy and safety of the drug (named “covered studies” by the FDA), the Investigator and all sub-Investigators are obliged to disclose their financial interest which they or their spouses and dependent children may have in the Sponsor. This information is required during the study and until 12 months after its completion.

### 11.4. Participant Information and Informed Consent

A prerequisite for a participant participating in the study is his/her or their legal representative or parent’s/guardian’s written informed consent. Adequate information must therefore be given to the participant by the Investigator/designated personnel before informed consent is obtained. One or more Informed Consent Forms in the local language

and prepared in accordance with the Note for Guidance on Good Clinical Practice (ICH, Topic E6 (R2)) will be provided by the Sponsor for the purpose of obtaining informed consent. In addition to this written information, the Investigator or his designate will inform the participant verbally. In doing so, the wording used will be chosen so that the information can be fully and readily understood by laypersons. The Informed Consent Form will be revised whenever important new information becomes available that may be relevant to the consent of participants.

The participant must be informed that his/her personal trial-related data will be used by CNBG and his subsidiaries (Sponsor) in accordance with the local data protection law. The level of disclosure must also be explained to the participant.

The participant must be informed that his/her medical records may be inspected by authorized monitors or Clinical Quality Control personnel appointed by CNBG and its subsidiaries (Sponsor), by appropriate IRB/IEC members, and by inspectors from regulatory authorities.

CONFIDENTIAL

The Informed Consent Form must also be signed and personally dated by the participant and legal representative or parents/guardian and by the Investigator/person designated by the Investigator to conduct the informed consent discussion. Provision of consent will be confirmed in the eCRF by the Investigator. The signed and dated declaration of informed consent will remain at the Investigator's site and must be safely archived by the Investigator so that the forms can be retrieved at any time for monitoring, auditing and inspection purposes. A copy of the signed and dated information and consent should be provided to the participant prior to participation.

### 11.5. Compensation to Participants

Appropriate insurance coverage is provided by China National Biotec Group (CNBG) in line with legal requirements and GCP guidance. Details can be consulted at the study site (certificates and conditions in the Investigator Site File). Insurance coverage will be

provided by a local insurance provider.

### 11.6. Participant Confidentiality

The investigator(s) will respect and protect the confidentiality of the participant in all possible ways. Participant identification, other than participant number, initials and date of birth, will not appear in any Case Report Form (eCRF) pages or other documents given to the Sponsor. Only the investigator and the persons authorized to verify the quality and integrity of the study will have access to participant records where the participant can be identified.

Ensure that the personal privacy of the participants will not be disclosed during the steps of testing, biological sample collection, reporting and publication, etc. Only the participant code, blood sample number, blood collection time and test index are recorded on the package of blood sample. It is strictly limited to the core testing personnel to obtain electronic or written copies.

CONFIDENTIAL

### 11.7. Amendment to Participant Related Information

Should a Protocol amendment become necessary, the participant information and consent form may need to be revised to reflect the changes to the Protocol. It is the responsibility of the investigator to ensure that an amended consent form is reviewed and has received approval/informed opinion from the IRB/IEC and CRA and/or the regulatory authority (depending on local laws and regulations), and that it is signed by all participants subsequently entered in the trial and those currently in the trial, if affected by the amendment.

### 11.8. Direct Access to Source Documentation

Source data are all the information in original records and certified copies of original records of clinical findings, observations, or other activities in the study, which are necessary for the reconstruction and evaluation of the study. Source data are contained in source documents (originals or certified copies).

Source Documents are original documents, data, and records (e.g., clinical diagnosis charts,

CONFIDENTIAL

laboratory notes, memoranda, participants' diaries, pharmacy dispensing records, recorded data from automated instruments, copies or transcriptions certified after verification as being accurate and complete, participant files, pharmacy records or prescriptions, laboratory reports or electronic results printouts from the laboratory websites, vaccine handover sheet and delivery notes). All of them are expected to be reviewed signed, dated and assessed by investigator. The source documents must contain study participation information.

It is the Investigator’s obligation to collect and present all relevant medical data in the participant’s medical file. Sponsor name and trial number, participant identification (name, date of birth, address, etc.), information that the informed consent was obtained prior any screening procedures, visit dates, participant number, vaccine information, efficacy data, safety data, concomitant medications and date and reason of completion of the study.

Source records should be preserved for the maximum period of time in accordance with the local requirements. All information recorded on the eCRFs for this study must be consistent with the participant’s source documentation.

CONFIDENTIAL

Besides the monitor of the Contract Research Organization (CRO), regulatory authorities, members of ethics committees and the Sponsor's clinical quality assurance group or any other Sponsor’s representative, may carry out source data checks and/or on-site audits or inspections. Direct access to source data will be required for these audits and inspections; the execution of those activities will take considerations of data protection and medical confidentiality. The investigator will always provide necessary support to the CRO and the Sponsor.

# 12. STUDY MANAGEMENT

## 12.1. Case Report Form (eCRF) Handling

The data recorded during the course of this study will be documented in the form of eCRF, and must be forwarded to the Sponsor or appointed designee. Then they should be processed, evaluated and stored in anonymous form in accordance with data protection regulations.

CONFIDENTIAL

The investigator must ensure that the eCRFs and any other associated documents contain no mention of any participant names and other privacy information. The eCRFs must be completely filled in. They are regulatory documents and must be suitable for submission to authorities.

All data in the eCRFs must be derived from source documentations. Clinical data will be captured via local medical system, the data will be transmitted to CTMS and generate eCRF. The investigator site staff will enter and edit the data via a secure network, with secure access features (username, password and secure identification or username and password – an electronic password system). A complete electronic audit trail will be maintained. The investigator will approve the data using an electronic signature (Ref: 21 CFR Part 11), and this approval is used to confirm the accuracy of the data recorded. The electronic CRFs (eCRFs) will be used, the investigator's data will be accessible from the investigator's site throughout the trial. The electronic CRFs must be kept current to reflect participant status at each phase during the trial. The participants must not be identified on the electronic CRF by name. Appropriate identifier (i.e. Participant Number) must be used. The investigator must make a separate confidential record of these details (participant identification code list) to permit identification of all participants enrolled in a clinical trial in case follow-up is required. While a trial is ongoing and before locking of the database, there will be no modifications in the form of Documentation of Changes (DOCs).

## 12.2. Source Data and Participant Files

Source documents provide evidence for the existence of the participant and substantiate the integrity of the data collected. Source documents are filed at the investigator's site.

The investigator has to keep a written or electronic participant file for every participant participating in the clinical study. In this participant file, the available demographic and medical information of a participant has to be documented, in particular the following: name, date of birth, sex, height, weight, records on inclusion and exclusion assessment, statement of entering the study, study identification, randomization number, the date of informed consent, all study visit dates, predefined examinations and clinical symptoms, monitored AEs, and reason for withdrawal from the study, if applicable.

CONFIDENTIAL

It should be possible to verify the inclusion and exclusion criteria defined in the protocol from the available data in this file. It must be possible to identify each participant by using this participant file. Additionally, any other documents with source data, especially original printouts of data that were generated by technical equipment have to be filed. This includes but not limited to ECG, X-ray films, CT, laboratory testing values and questionnaires, etc. All these documents must have at least the participant identifier and the printing date printed by the recording device to indicate to which participant and to which study visiting period the document belongs. The medical evaluation of such records should be documented as necessary and signed/dated by the investigator.

Electronic participant files will be printed whenever source data verification is performed by the monitor. Printouts must be signed and dated by the investigator, countersigned by the monitor and kept in a secured place.

Data transcribed from source documents to the eCRFs must be consistent with the source documents or the discrepancies must be explained. The investigator may need to request previous medical records or transfer records, depending on the case; also, current medical records must be provided.

Data on the participant dairies are considered source data and have to be stored along with the participant file. The data of the dairies will be forwarded to the Sponsor or the CRO appointed by the Sponsor for data entry into the database.

### 12.3. Investigator Site File and Archiving

The Investigator will be provided with an Investigator Site File at the start of the study. This file contains all relevant documents necessary for the conduct of the study. This file must be safely archived after termination of the study. It is the responsibility of the Investigator to ensure that the files are stored for at least 15 years beyond the end of the clinical study. All original participant files must be stored at the hospital or research institution for the longest possible time permitted by the regulations. If archiving can no longer be maintained at the site, the Investigator should notify the Sponsor.

CONFIDENTIAL

## 12.4. Monitoring, Quality Assurance and RA Inspection

This study is to be conducted in accordance with the ICH Note for Guidance on Good Clinical Practice (ICH, E6 (R2), 2016). The appointed clinical monitor will arrange regular visits to the study center(s) to check the progress of the study and the completion of eCRF. During monitoring visits, the monitors will:

- Help resolve any problems;
- Examine eCRF for omission of data, compliance and possible AEs;
- Discuss inconsistencies in the study data;
- Ensure that all study materials are correctly stored and allocated;
- Check adherence to the obligations of the investigator;
- Review consent forms, in particular the date of consent and signature;
- Perform source data verification as described below.

In line with International Conference on Harmonization (ICH)-Good Clinical Practice (GCP) guidelines, monitoring will include verification of data entered in the eCRF against original participant records. This verification will be performed by direct access to the original participant records, and the Sponsor guarantees that participant confidentiality will be respected at all times. Participation in this study will be taken as agreement to permit direct source data verification.

The investigator/institution will permit trial-related monitoring, audits, IRB/IEC review and regulatory inspection, providing direct access to all related source data/documents. eCRFs and all source documents, including progress notes and copies of laboratory and medical test results must be available at all times for review by the Sponsor's clinical trial auditor or a CRO appointed by the Sponsor, auditor and inspection by health regulatory authorities (e.g. FDA). The Clinical Research Associate (CRA) and auditor may review all CRFs/eCRFs, and signed informed consents.

## 12.5. Amendment to the Study Protocol

Changes to, or formal clarifications of, the study protocol must be documented in writing. Major changes to the protocol will be described in a "Protocol Amendment". It will be submitted to the relevant Ethics Committee(s)/Institutional Review Board(s) and to

CONFIDENTIAL

authorities, where required. Approval/favorable opinion from the relevant Ethics Committee(s)/Institutional Review Board(s) will be required prior to implementation of the amendment.

Any amendment affecting the participant requires the participant's informed consent prior to implementation. Changes of administrative or technical nature will be recorded in a document entitled "Administrative Change to Study Protocol". It will be sent for information to the relevant Ethics Committee(s)/Institutional Review Board(s) or to authorities, if so required. Amendments and administrative changes will be signed by all signatories of the protocol.

All Investigators will acknowledge the receipt and confirm by their signature on the Amendment or Administrative Change Signature Sheet that they will adhere to the Amendment/Administrative Change. This sheet will be issued in duplicate and after signing, one will be filed in the Investigator Site File and one in the Study Master File.

CONFIDENTIAL

### 12.6. Study Report and Publication Policy

After conclusion of the study, an integrated clinical and statistical study report shall be written by the Sponsor. The respective EC and competent authority need to be notified about the end of the trial (last participant/participant out) or early termination of the trial.

China National Biotec Group (CNBG) is as much as possible dedicated to support process of free exchange of relevant scientific information. Any publication of the result of this trial must be consistent with the CNBG publication policy. The rights of the investigator and of the Sponsor with regard to publication of the results of this trial are described in the investigator contract. As a general rule, no trial results should be published prior to finalization of the Clinical Trial Report (CTR).

The present trial will be published in a clinical trial registry indicating the trial dates and indication as well as the number of sites and location. The participant identity should be kept confidential.

# 13. REFERENCES

[1] Law of the People's Republic of China on Vaccine Administration, National People's Congress of the People's Republic of China, December 1, 2019

[2] Good Clinical Practice (GCP), NMPA, April 26, 2020

[3] Technical Guidelines for Vaccine Clinical Trials, NMPA, December 3, 2004

[4] "Technical Guidelines for Clinical Research of Vaccines for Novel Coronavirus Prevention (Trial)", NMPA, August 14, 2020

[5] "Guidelines for Writing the Basic Content of Vaccine Clinical Study Report," NMPA, October 12, 2005

[6] "Guidelines for Grading Adverse Events in Clinical Trials of Vaccines for Prophylactic Use," NMPA, December 31, 2019

[7] Measures for Administration of Drug Registration (Bureau Order No. 27) State Administration for Market Regulation, March 30, 2020

[8] "Guidelines for Ethical Review of Drug Clinical Trials", NMPA, November 2, 2010

[9] Laboratory Management Guidelines for Biological Sample Analysis in Drug Clinical Trials (Trial), NMPA, 2011

[10] "Guidelines for Quality Management of Vaccine Clinical Trials (Trial)", NMPA, October 31, 2013

[11] Regulations on the Administration of Drug Clinical Trial Institutions, NMPA, National Health Commission, December 1, 2019

[12] "Regulations on the Administration of Reporting Serious Adverse Events in Vaccine Clinical Trials (Trial)", NMPA, January 17, 2014

[13] "Guidelines for Biostatistics for Drug Clinical Trials," NMPA, June 1, 2016

[14] "Guidelines for Planning and Reporting for Drug Clinical Trial Data Management and Statistical Analysis," NMPA, January 4, 2022

[15] "Criteria and Procedures for Rapid Reporting of Safety Data During Drug Clinical Trials," NMPA, April 27, 2018

[16] ICH E6（R2）, Integrated Addendum to Good Clinical Practice, dated 9 November 2016

[17] ICH E3, Structure and Content of Clinical Summary Report, November 30, 1995

[18] ICH E9, Statistical Principles of Clinical Trials, February 5, 1998

CONFIDENTIAL

CONFIDENTIAL

[19] "Measures for the Identification of Abnormal Reactions to Vaccination", National Health Commission, December 1, 2008

[20] "Good Practice for Vaccination", National Health Commission, December 6, 2016

[21] "Good Practice for Vaccine Storage and Transportation" (2017 edition), National Health Commission, December 15, 2017

[22] Al Kaabi N, Zhang Y, Xia S. Effect of 2 Inactivated SARS-CoV-2 Vaccines on Symptomatic COVID-19 Infection in Adults: A Randomized Clinical Trial. JAMA. 2021 May 26. doi: 10.1001/jama.2021.8565. Epub ahead of print. PMID: 34037666.

[23] The Development Safety Update Report (DSUR): Harmonizing the Format and Content for Periodic Safety Reporting During Clinical Trials: Report of CIOMS Working Group VII, Geneva 2007. https://cioms.ch/shop/product/development-safety-update-report-dsur-harmonizing-format-contentperiodic-safety-report-clinical-trials-report-cioms-working-group-vii.

[24] Priority List of Adverse Events of Special Interest: COVID-19. SPEAC. 25 May 2020.
https://brightoncollaboration.us/wpcontent/uploads/2020/06/SPEAC_D2.3_V2.0_COVID-19_20200525_public.pdf.

CONFIDENTIAL

[25] Coronavirus disease (COVID-2019) situation reports. WHO.
https://www.who.int/emergencies/diseases/novel-coronavirus-2019/situation-reports/

[26] Draft landscape and tracker of COVID-19 candidate vaccines. https://www.who.int/publications/m/item/draft-landscape-of-covid-19-candidate-vaccines

[27] Update on the covid-19 outbreak as of 24:00 on January 07 http://www.nhc.gov.cn/xcs/yqtb/202109/110e53f7872c4d6b96cf0be8f9d4faa2.shtml

[28] Guan WJ, Ni ZY, Hu Y, et al. Clinical characteristics of 2019 novel coronavirus infection in China. medRxiv. Published February 9, 2020. Accessed February 23,2020. https://www.medrxiv.org/content/10.1101/2020.02.06.20020974v1

[29] Zhou, P. et al. A pneumonia outbreak associated with a new coronavirus of probable bat origin. Nature https:// doi.org/10.1038/s41586-020-2012-7 (2020).

[30] Wrapp D, Wang NW, Corbett KS, et al. Cryo-EM Structure of the 2019-nCoV Spike in the Prefusion Conformation. bioRxiv. /b11>Published February 15, 2020. Accessed February 23, 2020.

[31] Zhang Y, Zeng G, Pan H, et al. Safety, tolerability, and immunogenicity of an

CONFIDENTIAL

inactivated SARS-CoV-2 vaccine in healthy adults aged 18-59 years: a randomised, double-blind, placebo-controlled, phase 1/2 clinical trial. Lancet Infect Dis. 2021 Feb; 21(2):181-192. doi: 10.1016/S1473-3099(20)30843-4.

[32] Novavax COVID-19 Vaccine Demonstrates 89.3% Efficacy in UK Phase 3 Trial. https://ir.novavax.com/news-releases/news-release-details/novavax-covid-19-vaccine-demonstrates-893-efficacy-uk-phase-3

[33] Shilong Yang, Yan Li, Lianpan Dai, et al. Safety and immunogenicity of a recombinant tandem-repeat dimeric RBD protein vaccine against COVID-19 in adults: pooled analysis of two randomized, double-blind, placebo-controlled, phase 1 and 2 trials. medRxiv 2020.12.20.20248602; doi: https://doi.org/10.1101/2020.12.20.20248602

[34] EMA recommends COVID-19 Vaccine AstraZeneca for authorisation in the EU. https://www.ema.europa.eu/en/news/ema-recommends-covid-19-vaccine-astrazeneca-authorisation-eu

[35] Voysey M, Clemens SAC, Madhi SA, et al. Safety and efficacy of the ChAdOx1 nCoV-19 vaccine (AZD1222) against SARS-CoV-2: an interim analysis of four randomised controlled trials in Brazil, South Africa, and the UK. Lancet. 2021, 397(10269):99-111. doi: 10.1016/S0140-6736(20)32661-1.

[36] Zhu FC, et al. Immunogenicity and safety of a recombinant adenovirus type-5-vectored COVID-19 vaccine in healthy adults aged 18 years or older: a randomised, double-blind, placebo-controlled, phase 2 trial. Lancet. 2020, 396(10249):479-488. doi: 10.1016/S0140-6736(20)31605-6.

[37] Baden LR, El Sahly HM, Essink B, et al. Efficacy and Safety of the mRNA-1273 SARS-CoV-2 Vaccine. N Engl J Med.2020 Dec 30: NEJMoa2035389. doi: 10.1056/NEJMoa2035389. Epub ahead of print. PMID: 33378609

[38] Polack FP, Thomas SJ, Kitchin N, et al. Safety and Efficacy of the BNT162b2 mRNA Covid-19 Vaccine. N Engl J Med. 2020,383(27):2603-2615. doi: 10.1056/NEJMoa2034577.

[39] Technical Guidelines for COVID-19 Vaccination (1st Edition), National Health Commission.

CONFIDENTIAL

# Appendix

## Case Definition and Determination Criteria

### Case Definition

**1.Suspected cases:**

Comprehensive judgment based on epidemiological history and clinical symptoms:

Have any of the epidemiological history, and have two or more A symptoms, or have one or more B symptoms; or

with imaging features of COVID-19

If there is no clear epidemiological history, they should have two or more A symptoms or one or more B symptoms and detectable SARS-CoV-2 specific IgM; or have two or more A symptoms and One or more B symptoms; with imaging features of COVID-19

**① Epidemiological history**

A. Long-term residence or stay in the affected area for more than 7 days is deemed to have an epidemiological history; or

CONFIDENTIAL

B. History of travel or residence in the community where the case was reported within 14 days before the onset of illness; or

C. In contact with SARS-CoV-2 infected or asymptomatic infected persons within 14 days before the onset; or

D. Cluster cases (2 or more cases of fever and/or respiratory symptoms occurred in a small area such as home, office, school, etc. within 2 weeks).

**② Clinical symptoms**

Symptoms A (last for at least 2 days): fever (axillary temperature ≥37.5°C); chills; sore throat; fatigue; nasal congestion or runny nose; body pain, muscle pain; headache; nausea or vomiting; diarrhea.

Symptoms B: Cough (last for at least 2 days); newly developed taste or smell disorders (last for at least 2 days); shortness of breath or difficulty breathing;

③ Imaging features of COVID-19.

**2. Confirmed cases**:

CONFIDENTIAL

On the basis of the determination of the suspected case, there's also a COVID-19 PCR test result.

**3. Differential diagnosis**:

Encourage any possible pathogenic differential diagnosis.

**Clinical Classifications of Confirmed COVID-19 Cases:**

**Mild**

The clinical symptoms are mild, and there is no imaging characteristics of pneumonia.

**Moderate**

Showing fever, respiratory symptoms, and imaging characteristics of pneumonia.

**Severe**

CONFIDENTIAL

Meet any of the following criteria:

1. Respiratory distress (RR≥30 breaths/min);
2. Oxygen saturation≤93% at rest;
3. Arterial partial pressure of oxygen (PaO2)/ fraction of inspired oxygen (FiO2)≦300mmHg (1mmHg=0.133kPa);
4. The clinical symptoms progressively worsened, and the chest imaging showed >50% obvious lesion progression within 24-48 hours.

**Critical**

Meet one of the following criteria:

1. Respiratory failure and requiring mechanical ventilation;
2. Shock;
3. With other organ failure that requires ICU care;
4. Death

CONFIDENTIAL
